# Green and Facile Synthesis of Spirocyclopentanes Through NaOH-Promoted Chemo- and Diastereo-Selective (3 + 2) Cycloaddition Reactions of Activated Cyclopropanes and Enamides

**DOI:** 10.3389/fchem.2020.00542

**Published:** 2020-06-26

**Authors:** Xun Zhu, Dingwu Pan, Chengli Mou, Bo Zhou, Lutai Pan, Zhichao Jin

**Affiliations:** ^1^Laboratory Breeding Base of Green Pesticide and Agricultural Bioengineering, Key Laboratory of Green Pesticide and Agricultural Bioengineering Ministry of Education, Guizhou University, Guiyang, China; ^2^School of Pharmacy, Guizhou University of Traditional Chinese Medicine, Guiyang, China; ^3^R&D Center, Shenzhen AmTech Bioengineering Ltd., Inc., Shenzhen, China

**Keywords:** green, NaOH, donor-acceptor cyclopropane, (3 + 2) cycloaddition, spirocyclopentane, indole derivative

## Abstract

A chemo- and diastereo-selective (3 + 2) cycloaddition reacition between Donor-Acceptor (D-A) cyclopropanes and α,β-unsaturated enamides is developed for efficient access to spiro(cyclopentane-1,3'-indoline) derivatives. Simple, inexpensive and readily available NaOH is used as the sole catalyst for this process. A broad range of D-A cyclopropanes could be used as the C-3 synthons to react with oxindole-derived α,β-unsaturated enamides. The structurally sophisticated spiro(cyclopentane-1,3'-indoline) derivatives bearing up to 3 adjacent chiral centers are afforded in excellent yields as single diastereomers.

## Introduction

**Figure 1:**
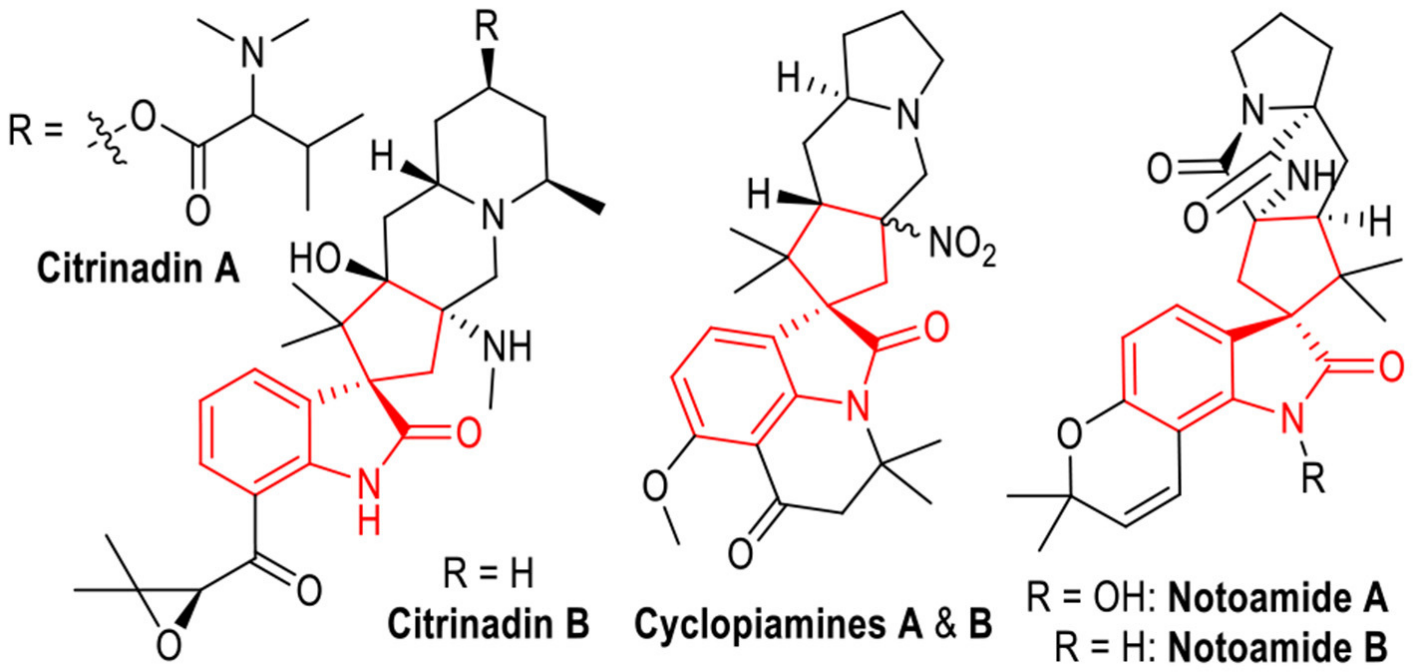
Bioactive Natural Products Containing Spiro(cyclopentane-1,3'-indoline) Units.

Spirocyclopentanes are interesting structural units with broad applications in organic synthesis and medicinal chemistry. They have existed as core structures in various bioactive molecules (Boeyens et al., 1979; Tsuda et al., 2004; Mugishima et al., 2005; Zhang et al., 2019). Specifically, spiro(cyclopentane-1,3'-indoline) derivatives are frequently found in natural products with proven biological activities (Figure 1). For example, Citrinadin A and B are active molecules against murine leukemia L1210 and human epidermoid carcinoma KB cells, which have been isolated from a culture broth of Penicillium citrinum. Cyclopiamines A and B are extracts from a toxinogenic strain of Penicillium cyclopium. The Notoamides A and B are key members of paraherquamide family which belongs to prenylated indole alkaloids that exhibit various bioactivities including antitumor, antibacterial, and insecticidal properties. Therefore, the development of efficient methods for the preparation of spiro(cyclopentane-1,3'-indoline) derivatives has attracted much interest. Success within this field has been achieved through both organocatalysis (Chen et al., 2009; Antonchick et al., 2010; Tan et al., 2011; Tian and Melchiorre, 2013; Zhang et al., 2016; Chaudhari et al., 2017) and transition metal catalysis (Trost et al., 2007; Brazeau et al., 2012; Ball-Jones et al., 2014; Deiana et al., 2014; Afewerki et al., 2015; Frost et al., 2015; Qiu et al., 2019). Despite of the great achievement obtained in the synthesis of spiro(cyclopentane-1,3'-indoline) molecules, the development of green and economical methods for efficient and stereoselective synthesis of them is still of great interest.

**Figure 2:**
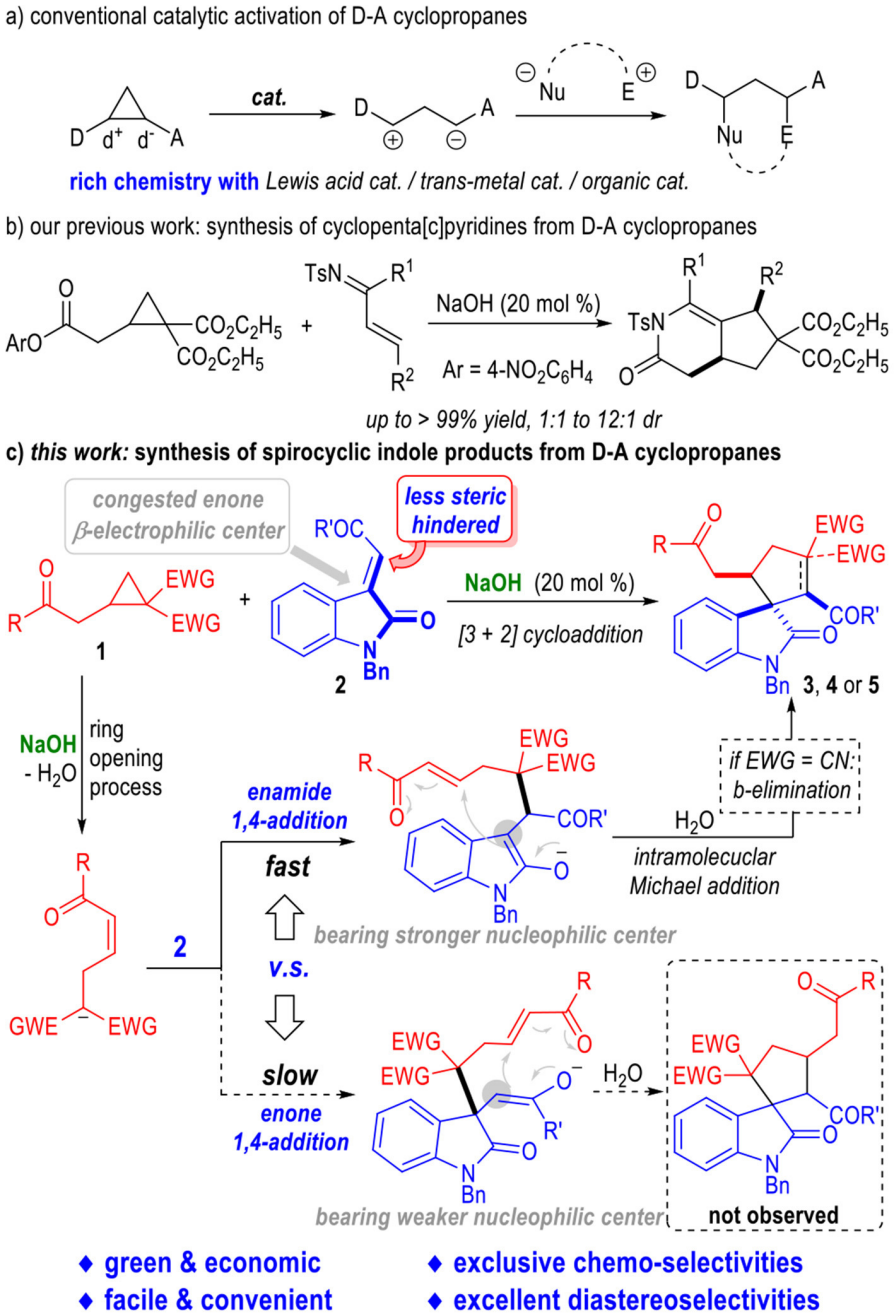
Catalytic Activation of D-A Cyclopropanes.

Cyclopropanes are important building blocks in organic synthesis (Sohn and Bode, 2006; Bode and Sohn, 2007; Li et al., 2009; Lv et al., 2011; Sparr and Gilmour, 2011; Halskov et al., 2015; Sanchez-Diez et al., 2016; Blom et al., 2017; Apel et al., 2019). Especially, the cyclopropanes bearing both an electron-donating and an electron-withdrawing group on their cyclic structures, which are commonly named as Donor-Acceptor (D-A) cyclopropanes (Danishefsky, 1979; Wenkert, 1980; Reissig and Zimmer, 2003; Carson and Kerr, 2009; Cavitt et al., 2014; Nanteuil et al., 2014; Schneider et al., 2014; Grover et al., 2015; Talukdar et al., 2016; Wang and Tang, 2016; Werz and Biju, 2019), have been extensively studied in the construction of various functional molecules. D-A Cyclopropanes are conventionally activated by transition metal catalysts (Nanteuil et al., 2014), Lewis acids (Reissig and Zimmer, 2003; Carson and Kerr, 2009; Cavitt et al., 2014; Schneider et al., 2014; Grover et al., 2015; Talukdar et al., 2016; Wang and Tang, 2016; Werz and Biju, 2019) or amine-based organic catalysts (Halskov et al., 2015; Sanchez-Diez et al., 2016; Blom et al., 2017) (Figure 2a). Efficient activation of D-A cyclopropanes by simple, inexpensive and readily available bases has been much less developed.

Very recently, we have disclosed that the D-A cyclopropanes could be activated by simple NaOH and reacted with α,β-unsaturated imines to give a variety of bioactive cyclopenta(c)pyridine derivatives in generally excellent yields and moderate diastereoselectivities (Pan et al., 2019) (Figure 2b). This approach has provided us with a green and facile method for the construction of structurally complex molecules from D-A cyclopropanes with simple and inexpensive reaction catalysts. Therefore, it is interesting and important to extend the border of this strategy to a wide range of substrates in order to get access to a broad scope of complex functional molecules in a green, facile, and economic fashion.

Herein, we disclose that the D-A cyclopropanes **1** can react with α,β-unsaturated enamide substrates **2** under basic conditions in chemoselective fashion (Figure 2c). Heavily substituted spiro(cyclopentane-1,3'-indoline) derivatives could be afforded in good to excellent yields. NaOH was used as the sole reaction catalyst. All the spirocyclic products bearing up to 3 adjacent chiral centers were afforded as single diastereomers. It is worth noting that both an enone and an enamide motif exist in the electrophilic substrate **2**. After deprotonation of the D-A cyclopropane substrate **1**, the afforded ring-opening intermediate **I** could selectively react with the electrophile **2** through an enamide 1,4-addition process and gave intermediate **II** bearing a highly reactive nucleophilic carbon center. The enamide 1,4-addition reaction was believed to go faster than the enone 1,4-addition reaction because that there were less steric hindrance around the enamide β-carbon. After an intramolecular Michael addition process, the spiral cyclopentane products **3** or **4** were afforded in excellent diastereoselectivities. Interestingly, an additional β-elemination could happen during this catalytic transformation when using the D-A cyclopropyl ketone bearing *gem*-dicyano groups as the reaction substrate. Spiral cyclopentenes **5** were afforded as the final products in this case. Product **6** that might be formed from the enone 1,4-addition intermediate **III** were not observed. The less nucleophilicity of the enol moiety of the intermediate **III** might be another reason for the difficult formation of the enone 1,4-addition products.

## Results and Discussion

### Reaction Condition Optimization and Large-Scale Reactions

The 2-cyclopropyl acetate **1a** and the oxindole-derived α,β-unsaturated enamide **2a** were selected as the model substrates to test the catalytic conditions for this (3 + 2) cycloaddition reaction (Table 1). A variety of inorganic bases were found efficient for this 1,3-dipolar cycloaddition reaction between 2-cyclopropyl acetate **1a** and the α,β-unsaturated enamide **2a** (Table 1, entries 1 to 3). The organic bases we tested were not suitable for this reaction (entries 4 to 5). The catalytic cyclization process could also be carried out in several organic solvents with relatively high polarities, although the yields were generally decreased (entries 6 to 7). Solvents with low polarities could not be used for this transformation (entries 8 to 9). The reaction temperature could be slightly decreased to 30°C without erosion of the product yield (entry 10). Finally, the yield of the spiro(cyclopentane-1,3'-indoline) product **3a** could be increased to 90% with a larger excess amount of **1a** used under the catalysis of NaOH in THF at 30°C (entry 11). Note that, all the products afforded in these reactions were obtained as single diastereomers.

**Table 1:** Optimization of Reaction Conditions^a^.

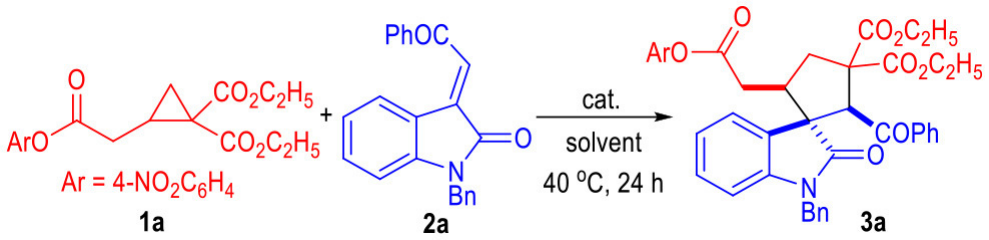				
**Entry**	**Cat**.	**Solvent**	**Yield (%)***^**b**^*	***dr**^**c**^*
1	NaOH	THF	82	>20:1
2	NaOCH_3_	THF	80	>20:1
3	K_2_CO_3_	THF	74	>20:1
4	DBU	THF	<5	
5	Et_3_N	THF	<5	
6	NaOH	EtOAc	72	>20:1
7	NaOH	CH_3_CN	25	>20:1
8	NaOH	CH_2_Cl_2_	<5	
9	NaOH	PhCH_3_	<5	
10*^*d*^*	NaOH	THF	83	>20:1
11*^*e*^*	NaOH	THF	90	>20:1
12*^*e*^*	NaOH	2-Me THF	72	>20:1
13*^*e*^*	NaOH	Anisole	<5	
14*^*e*^*	NaOH	H_2_O	<5	
15*^*e*^*	NaOH	EtOH	75	>20:1
16*^*f*^*	NaOH	EtOH	74	>20:1

a *General conditions (unless otherwise specified): **1a** (0.075 mmol), **2a** (0.05 mmol), cat. (0.01 mmol), THF (1.0 mL), 40°C, 24 h*.

b *Isolated yield of **3a***.

c *Dr was determined via ^1^H NMR on the crude product*.

d *The reaction was carried out at 30°C for 24 h*.

e ***1a** (0.1 mmol), **2a** (0.05 mmol), NaOH. (0.01 mmol), solvent (1.0 mL), 30°C, 24 h*.

f ***1a** (1.0 mmol), **2a** (0.5 mmol), NaOH. (0.1 mmol), EtOH (10.0 mL), 30°C, 24 h*.

We were also very interested in developing a green and efficient method for the construction of the spiro(cyclopentane-1,3'-indoline) product **3a**. Therefore, several green solvents were further examined after obtaining the optimized reaction condition (Table 1, entries 12 to 15). 2-Methyl-substituted THF could give the desired product in a good yield (entry 12). Anisole or water could not be used as the solvents for this transformation (entries 13 to 14). To our delight, the inexpensive and non-toxic ethanol could be used as a suitable medium for the construction of the spiro(cyclopentane-1,3'-indoline) products through this protocol (entry 15). Therefore, we carried out a large-scale reaction of the substrate **1a** and **2a** in ethanol, with the desired product **3a** afforded in a 74% yield as a single diastereomer (entry 16).

### Reaction Scope Investigation and Synthetic Application

With the optimized reaction conditions at hand (Table 1, entry 11), we then examined the substrate scope of this (3 + 2) cycloaddition reaction with respect to both D-A cyclopropyl acetates **1** and α,β-unsaturated enamides **2** (Tables 2–**4**).

**Table 2:** Scope of α,β Unsaturated Enamides **2**^a^.

** 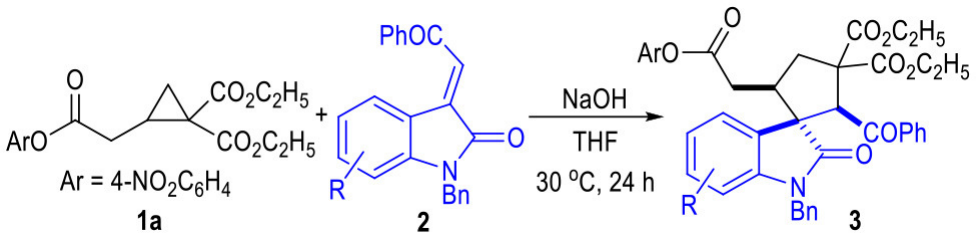 **
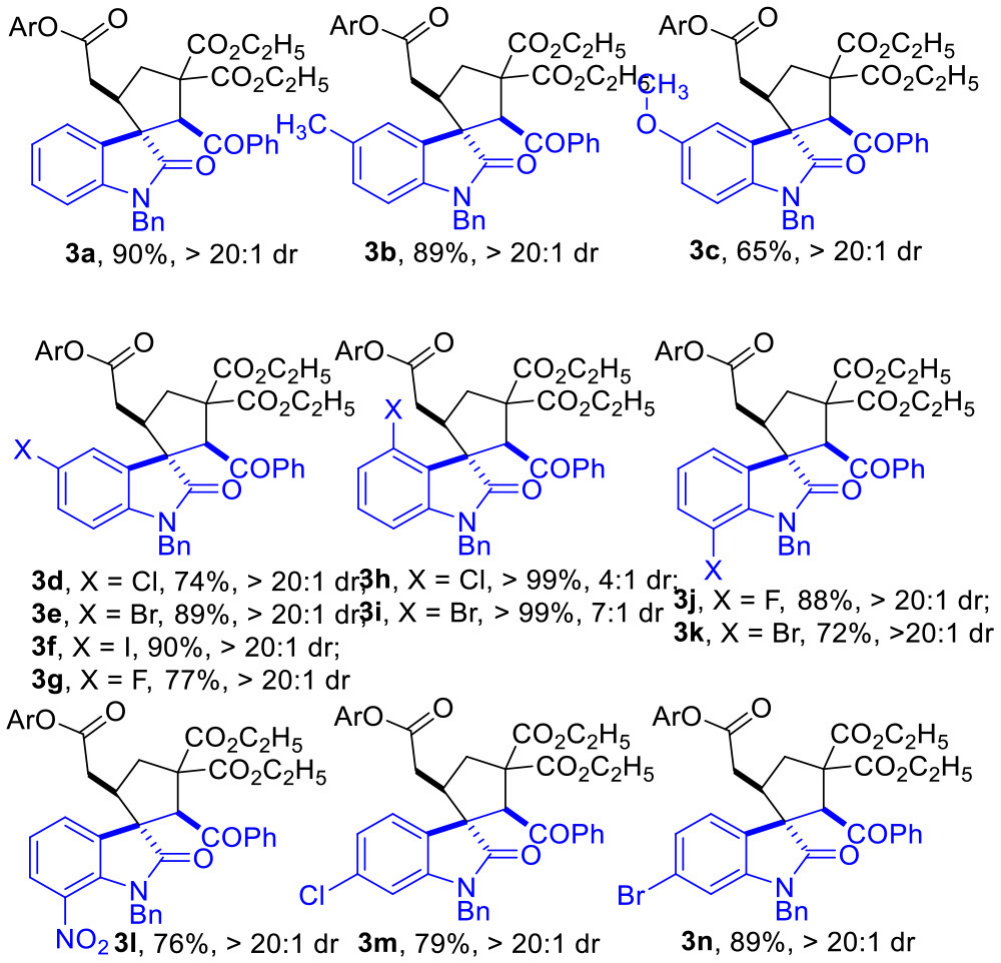

a *Reaction conditions as stated in Table 1, entry 11. Isolated yields are reported after purification via SiO_2_ column chromatography. Dr was determined via ^1^H NMR on the crude product*.

The R substituent on the phenyl group of the indoline motif of the α,β-unsaturated enamides **2** could be either electron-donating groups (Table 2, **3b and 3c**) or electron-withdrawing groups (**3d** to **3n**), with most of the spirocyclic products being afforded in good to excellent yields and diastereo-selectivities.

The R^1^ group of ketone moieties could be phenyl rings of different substitution patterns, with the corresponding products being afforded in excellent yields as single diastereomers (Table 3, **4a** to **4i**). Moreover, the R^1^ group could also be switched to a heteroaromatic group or a vinylogous phenyl group without erosion on the product yields or diastereoselectivities (**4j** to **4k**). Interestingly, the R^1^ group of the ketones **2** could even be replaced with a simple methyl or ethyoxyl group, and the corresponding products **4l** and **4m** could be afforded in good yields as single diastereomers. The *N-*protecting benzyl group of indoline motif could be replaced with an *N*-methyl group, and the desired product **4n** could also be afforded in a good yield as a single diastereomer. Unprotected isatin-derived enamide substrates were not effective in this transformation.

**Table 3:** Scope of α,β-Unsaturated Enamides **2**^a^.

** 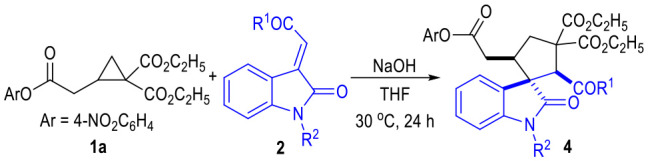 **
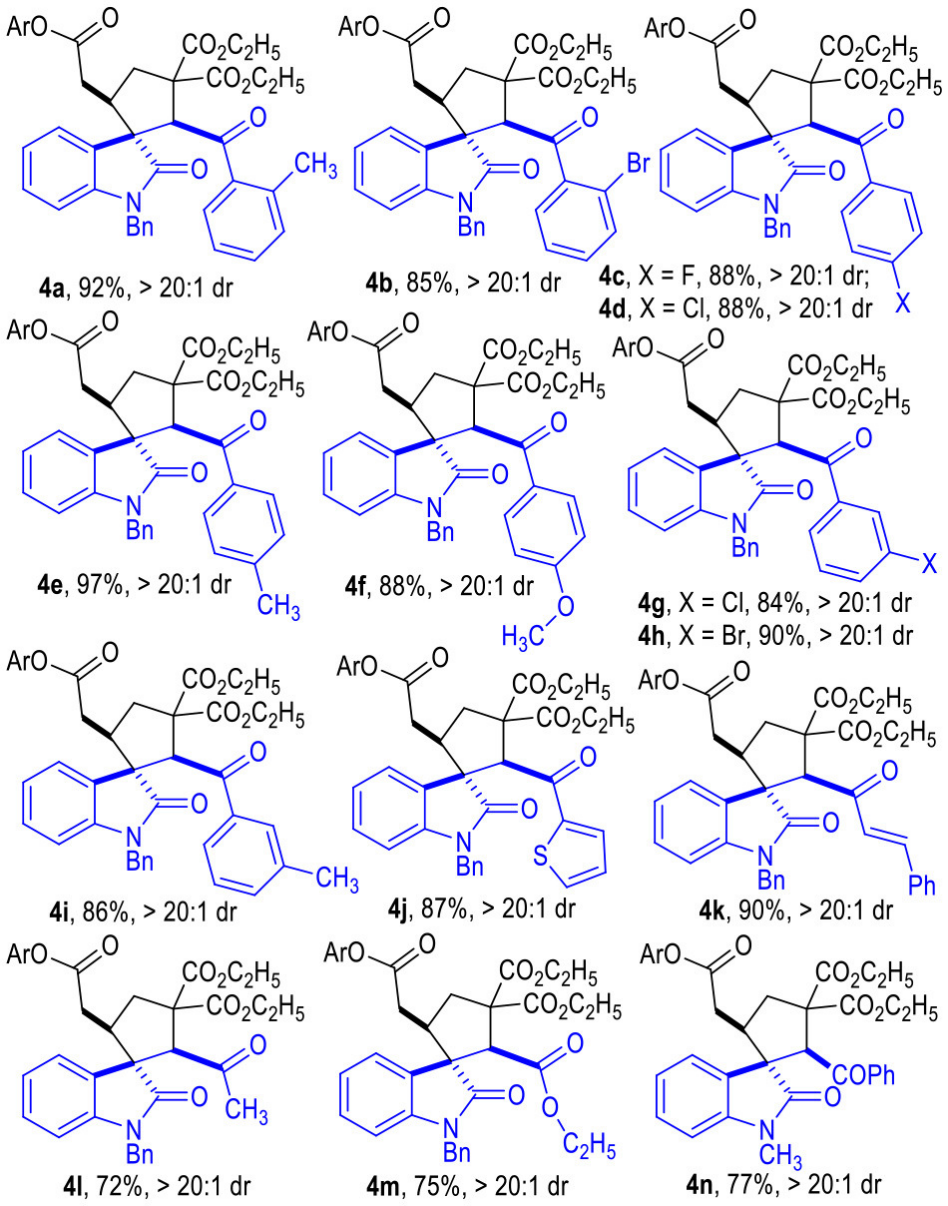

a *Reaction conditions as stated in Table 1, entry 11. Isolated yields are reported after purification via SiO_2_ column chromatography. Dr was determined via ^1^H NMR on the crude product*.

The scope of the D-A cyclopropyl acetates **1** was also examined (Table 4). The electron deficient 4-nitrophenol group on **1a** could be switched to an electron rich aromatic group (**1b**) without erosion on the reaction diastereoselectivity, although the yield of the product was slightly decreased to 86%. Replacing the R group on ester substrate **1** with a simple methyl group (**1c**) led to only trace formation of the target product. The sterically bulkier isopropyl ester (**1d**) also worked well in this transformation and afforded the desired product in a good yield as a single diastereomer. It is worth noting that the cyclopropyl aldehyde **1e** was not a suitable substrate for this (3 + 2) cycloaddition reaction.

**Table 4:** Scope of the D-A Cyclopropanes **1**^a^.

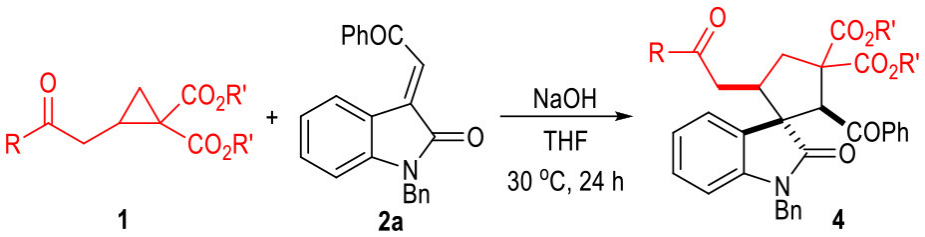					
**Entry**	**R**	**R'**	**4**	**Yield (%)**	***dr***
1	4-CH_3_OC_6_H_4_	CH_3_CH_2_	**4o**	86	> 20:1
2	4-NO_2_C_6_H_4_	(CH_3_)_2_CH	**4p**	79	> 20:1
3	CH_3_	CH_3_CH_2_	-	0	-
4	H	CH_3_CH_2_	-	0	-

a *Reaction conditions as stated in Table 1, entry 11. Isolated yields are reported after purification via SiO_2_ column chromatography. Dr was determined via ^1^H NMR on the crude product*.

To our great delight, the D-A cyclopropyl acetone **1f** bearing two cyano groups also worked well in the (3 + 2) cylcoaddition reaction with the oxindole-derived α,β-unsaturated enamide **2** under the current catalytic conditions (Table 5). The spirocyclopentenes **5** were afforded as the final products with the elimination of one equiv. of HCN. Both electron-donating and electron-withdrawing groups could be installed on the indoline moieties of the α,β-unsaturated enamides **2**, with the corresponding products being afforded in moderate yields as single diastereomers.

**Table 5:** Scope of α,β-Unsaturated Enamides **2**^a^.

** 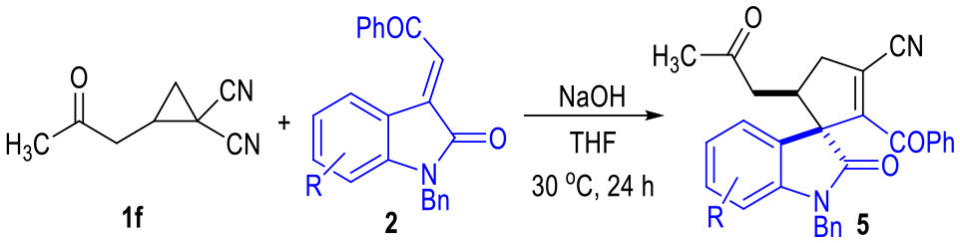 **
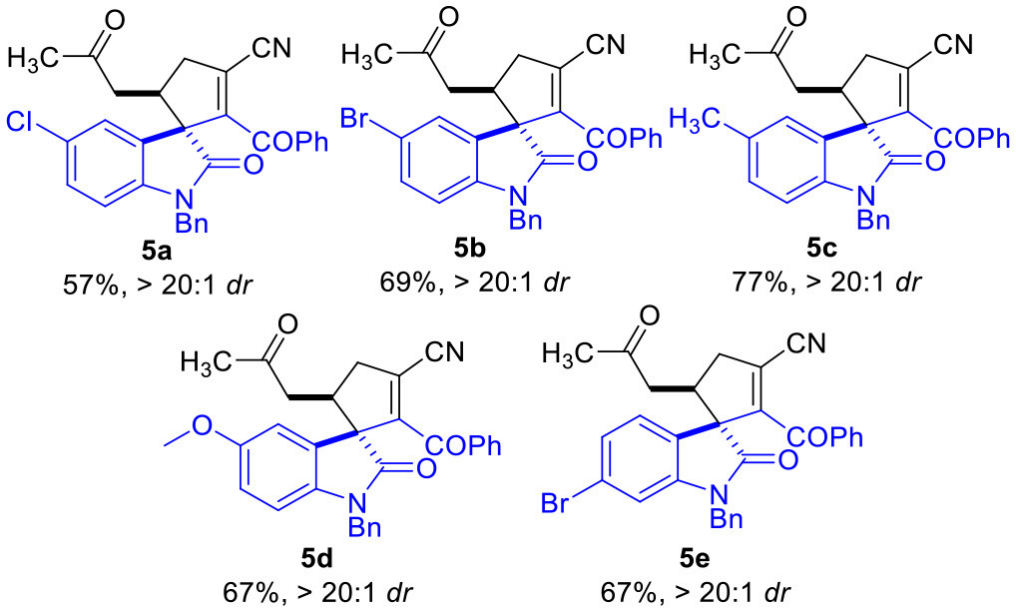

a *Reaction conditions as stated in Table 1, entry 11. Isolated yields are reported after purification via SiO_2_ column chromatography. Dr was determined via ^1^H NMR on the crude product*.

**Figure 3:**
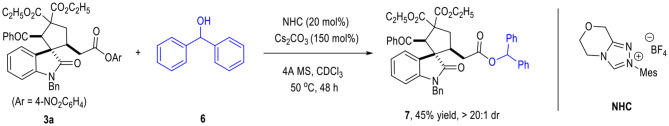
Synthetic transformations of **3a**.

The afforded spiro(cyclopentane-1,3'-indoline) product **3a** could be used as the reaction material for further transformations (Figure 3). For example, a trans-esterification reaction of **3a** could give other ester products (e.g., **7**) in moderate yields without erosion of the diastereomeric ratio.

## Conclusion

In conclusion, we have developed a chemo- and diastereo-selective (3 + 2) cycloaddition between D-A cyclopropanes and α,β-unsaturated enones. Green, inexpensive, and readily available NaOH was used as the sole catalyst to promote this transformation. Structurally sophisticated spiro(cyclopentane-1,3'-indoline) derivatives bearing up to 3 adjacent chiral centers were afforded as the final products in generally good to excellent yields as single diastereomers. This study could provide us with a green, facile and economic approach in preparing complex functional molecules through simple operations. Further investigations on the development of efficient methods for the construction of complex molecules are in progress in our laboratory.

## Data Availability Statement

The raw data supporting the conclusions of this article will be made available by the authors, without undue reservation.

## Author Contributions

XZ and DP conducted most of the experiments. CM, LP, and ZJ conceptualized and directed the whole project. ZJ drafted the manuscript. BZ participated in some experimental work and manuscript writing. All of the authors contributed in scientific discussions. All authors contributed to the article and approved the submitted version.

## Conflict of Interest

BZ was employed by the company Shenzhen AmTech Bioengineering Ltd. The remaining authors declare that the research was conducted in the absence of any commercial or financial relationships that could be construed as a potential conflict of interest.
