# Supplementary material for: Green and Facile Synthesis of Spirocyclopentanes Through NaOH-Promoted Chemo- and Diastereo-Selective (3 + 2) Cycloaddition Reactions of Activated Cyclopropanes and Enamides
Source: Front Chem. 2020 Jun 26;8:542. doi: 10.3389/fchem.2020.00542 (PMC7333539; doi:10.3389/fchem.2020.00542)

checkCIF/PLATON report

You have not supplied any structure factors. As a result the full set of tests cannot be run.

THIS REPORT IS FOR GUIDANCE ONLY. IF USED AS PART OF A REVIEW PROCEDURE FOR PUBLICATION, IT SHOULD NOT REPLACE THE EXPERTISE OF AN EXPERIENCED CRYSTALLOGRAPHIC REFEREE.

No syntax errors found. CIF dictionary Interpreting this report

Datablock: 181229a

```
Bond precision:  C-C = 0.0165 A          Wavelength=0.71073

Cell:          a=10.2560(9)      b=14.1850(12)    c=14.4350(13)
               alpha=62.399(1)   beta=79.724(2)   gamma=85.725(4)
Temperature:   298 K

                Calculated                 Reported
Volume          1831.1(3)                  1831.1(3)
Space group     P -1                       P-1
Hall group      -P 1                       ?
Moiety formula  C40 H35 Br N2 O10          ?
Sum formula     C40 H35 Br N2 O10          C40 H35 Br N2 O10
Mr              783.60                     783.61
Dx,g cm-3       1.421                      1.421
Z               2                          2
Mu (mm-1)       1.185                      1.185
F000            808.0                      808.0
F000'           807.82
h,k,lmax        12,16,17                   12,16,17
Nref            6469                       6362
Tmin,Tmax       0.892,0.942                0.789,0.943
Tmin'           0.780

Correction method= # Reported T Limits: Tmin=0.789 Tmax=0.943
AbsCorr = MULTI-SCAN

Data completeness= 0.983          Theta(max)= 25.020

R(reflections)= 0.0931( 1941)     wR2(reflections)= 0.2259( 6362)

S = 1.062                  Npar= 520
```

The following ALERTS were generated. Each ALERT has the format
test-name_ALERT_alert-type_alert-level.
Click on the hyperlinks for more details of the test.

Alert level B

```
PLAT026_ALERT_3_B Ratio Observed / Unique Reflections (too) Low ..        31% Check
PLAT234_ALERT_4_B Large Hirshfeld Difference C26      --C27      .       0.28 Ang.
PLAT331_ALERT_2_B Small Aver Phenyl C-C Dist C10      --C15      .       1.36 Ang.
PLAT341_ALERT_3_B Low Bond Precision on  C-C Bonds ...............     0.0165 Ang.
```

Alert level C

```
PLAT094_ALERT_2_C Ratio of Maximum / Minimum Residual Density ....       2.81 Report
PLAT234_ALERT_4_C Large Hirshfeld Difference O9       --N2       .       0.16 Ang.
PLAT234_ALERT_4_C Large Hirshfeld Difference N2       --C38      .       0.19 Ang.
PLAT234_ALERT_4_C Large Hirshfeld Difference C9       --C10      .       0.17 Ang.
PLAT234_ALERT_4_C Large Hirshfeld Difference C13      --C14      .       0.19 Ang.
PLAT234_ALERT_4_C Large Hirshfeld Difference C14      --C15      .       0.17 Ang.
PLAT234_ALERT_4_C Large Hirshfeld Difference C36      --C37      .       0.16 Ang.
PLAT241_ALERT_2_C High   MainMol Ueq as Compared to Neighbors of         O7 Check
PLAT241_ALERT_2_C High   MainMol Ueq as Compared to Neighbors of        C12 Check
PLAT241_ALERT_2_C High   MainMol Ueq as Compared to Neighbors of        C27 Check
PLAT241_ALERT_2_C High   MainMol Ueq as Compared to Neighbors of        C39 Check
PLAT241_ALERT_2_C High   MainMol Ueq as Compared to Neighbors of        C40 Check
PLAT242_ALERT_2_C Low    MainMol Ueq as Compared to Neighbors of         N2 Check
PLAT242_ALERT_2_C Low    MainMol Ueq as Compared to Neighbors of        C10 Check
PLAT242_ALERT_2_C Low    MainMol Ueq as Compared to Neighbors of        C32 Check
PLAT242_ALERT_2_C Low    MainMol Ueq as Compared to Neighbors of        C35 Check
PLAT242_ALERT_2_C Low    MainMol Ueq as Compared to Neighbors of        C38 Check
PLAT334_ALERT_2_C Small Aver. Benzene C-C Dist C35      -C40             1.36 Ang.
PLAT410_ALERT_2_C Short Intra H...H Contact  H21      ..H24      .       1.98 Ang.
                                                      x,y,z  =      1_555 Check
```

Alert level G

```
PLAT005_ALERT_5_G No Embedded Refinement Details Found  in the CIF     Please Do !
PLAT066_ALERT_1_G Predicted and Reported Tmin&Tmax Range Identical          ? Check
PLAT093_ALERT_1_G No s.u.'s on H-positions, Refinement Reported as      mixed Check
PLAT180_ALERT_4_G Check Cell Rounding: # of Values Ending with 0 =          3 Note
PLAT301_ALERT_3_G Main Residue  Disorder ..............(Resd  1  )         8% Note
PLAT398_ALERT_2_G Deviating  C-O-C    Angle From 120 for O5             105.6 Degree
PLAT413_ALERT_2_G Short Inter XH3 .. XHn     H17B     ..H34E     .       1.60 Ang.
                                                    1+x,y,z  =      1_655 Check
PLAT432_ALERT_2_G Short Inter X...Y Contact  O2       ..C34'             2.94 Ang.
                                                    1+x,y,z  =      1_655 Check
PLAT432_ALERT_2_G Short Inter X...Y Contact  C17      ..C34'             2.98 Ang.
                                                    1+x,y,z  =      1_655 Check
PLAT721_ALERT_1_G Bond    Calc     0.96000, Rep     0.97000 Dev...       0.01 Ang.
              C30      -H30B          1.555   1.555 ........     #   76 Check
PLAT721_ALERT_1_G Bond    Calc     0.97000, Rep     0.96000 Dev...       0.01 Ang.
              C31'     -H31F          1.555   1.555 ........     #   85 Check
PLAT779_ALERT_4_G Suspect or Irrelevant (Bond) Angle(s) in CIF . #         10 Check
              C30  -O5   -C30'    1.555   1.555   1.555             20.00 Deg.
PLAT779_ALERT_4_G Suspect or Irrelevant (Bond) Angle(s) in CIF . #         13 Check
              C33  -O7   -C33'    1.555   1.555   1.555             29.00 Deg.
PLAT793_ALERT_4_G Model has Chirality at C2          (Centro SPGR)          S Verify
PLAT793_ALERT_4_G Model has Chirality at C18         (Centro SPGR)          S Verify
PLAT793_ALERT_4_G Model has Chirality at C21         (Centro SPGR)          R Verify
PLAT899_ALERT_4_G SHELXL97   is Deprecated and Succeeded by SHELXL       2018 Note
```

0 **ALERT level A** = Most likely a serious problem - resolve or explain
4 **ALERT level B** = A potentially serious problem, consider carefully
19 **ALERT level C** = Check. Ensure it is not caused by an omission or oversight
17 **ALERT level G** = General information/check it is not something unexpected

```
   4 ALERT type 1 CIF construction/syntax error, inconsistent or missing data
  18 ALERT type 2 Indicator that the structure model may be wrong or deficient
   3 ALERT type 3 Indicator that the structure quality may be low
  14 ALERT type 4 Improvement, methodology, query or suggestion
   1 ALERT type 5 Informative message, check
```

Validation response form

Please find below a validation response form (VRF) that can be filled in and pasted into your CIF.

```
# start Validation Reply Form
_vrf_PLAT094_181229a
;
PROBLEM: Ratio of Maximum / Minimum Residual Density ....       2.81 Report
RESPONSE: ...
;
_vrf_PLAT234_181229a
;
PROBLEM: Large Hirshfeld Difference O9       --N2       .       0.16 Ang.
RESPONSE: ...
;
_vrf_PLAT241_181229a
;
PROBLEM: High   MainMol Ueq as Compared to Neighbors of         O7 Check
RESPONSE: ...
;
_vrf_PLAT242_181229a
;
PROBLEM: Low    MainMol Ueq as Compared to Neighbors of         N2 Check
RESPONSE: ...
;
_vrf_PLAT334_181229a
;
PROBLEM: Small Aver. Benzene C-C Dist C35      -C40             1.36 Ang.
RESPONSE: ...
;
_vrf_PLAT410_181229a
;
PROBLEM: Short Intra H...H Contact  H21      ..H24      .       1.98 Ang.
RESPONSE: ...
;
# end Validation Reply Form
```

It is advisable to attempt to resolve as many as possible of the alerts in all categories. Often the minor alerts point to easily fixed oversights, errors and omissions in your CIF or refinement strategy, so attention to these fine details can be worthwhile. In order to resolve some of the more serious problems it may be necessary to carry out additional measurements or structure refinements. However, the purpose of your study may justify the reported deviations and the more serious of these should normally be commented upon in the discussion or experimental section of a paper or in the "special_details" fields of the CIF. checkCIF was carefully designed to identify outliers and unusual parameters, but every test has its limitations and alerts that are not important in a particular case may appear. Conversely, the absence of alerts does not guarantee there are no aspects of the results needing attention. It is up to the individual to critically assess their own results and, if necessary, seek expert advice.

Publication of your CIF in IUCr journals

A basic structural check has been run on your CIF. These basic checks will be run on all CIFs submitted for publication in IUCr journals (*Acta Crystallographica*, *Journal of Applied Crystallography*, *Journal of Synchrotron Radiation*); however, if you intend to submit to *Acta Crystallographica Section C* or *E* or *IUCrData*, you should make sure that full publication checks are run on the final version of your CIF prior to submission.

Publication of your CIF in other journals

Please refer to the *Notes for Authors* of the relevant journal for any special instructions relating to CIF submission.

PLATON version of 22/12/2019; check.def file version of 13/12/2019

Datablock 181229a - ellipsoid plot

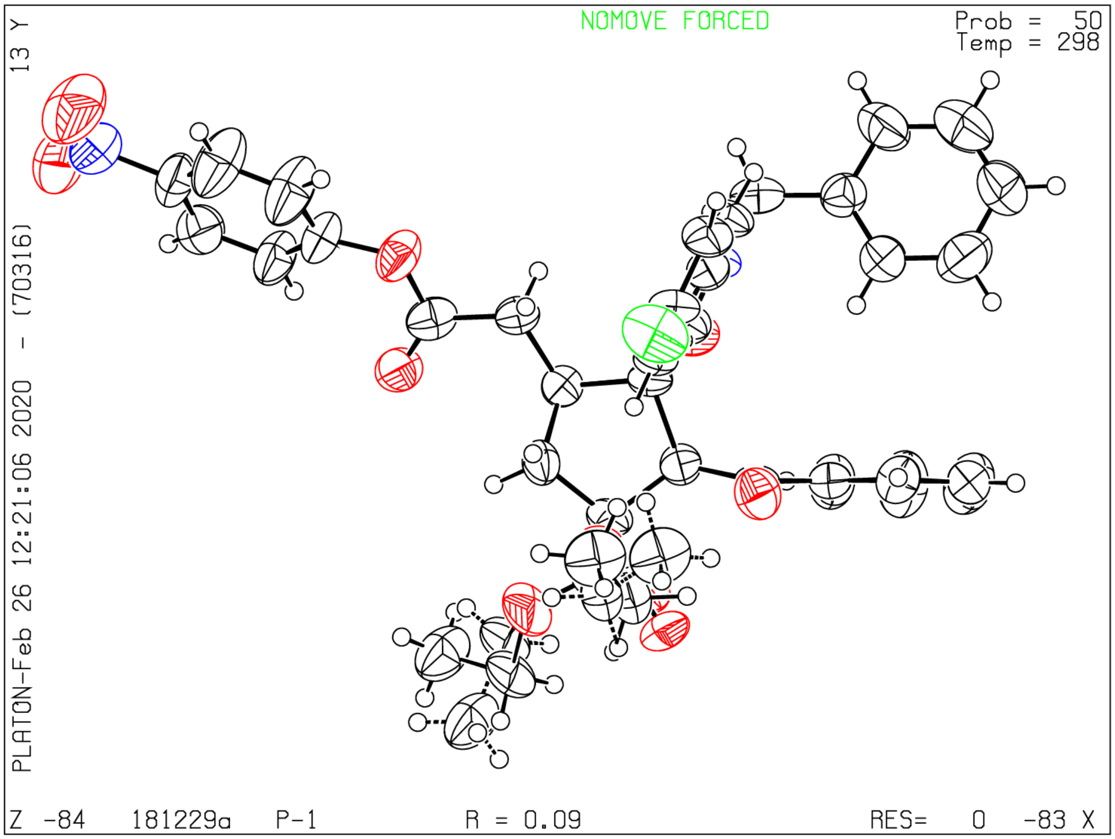

Supplement: Supplementary file 1 [file Data_Sheet_1.PDF]
