# Supplementary material for: Green and Facile Synthesis of Spirocyclopentanes Through NaOH-Promoted Chemo- and Diastereo-Selective (3 + 2) Cycloaddition Reactions of Activated Cyclopropanes and Enamides
Source: Front Chem. 2020 Jun 26;8:542. doi: 10.3389/fchem.2020.00542 (PMC7333539; doi:10.3389/fchem.2020.00542)

checkCIF/PLATON report

You have not supplied any structure factors. As a result the full set of tests cannot be run.

THIS REPORT IS FOR GUIDANCE ONLY. IF USED AS PART OF A REVIEW PROCEDURE FOR PUBLICATION, IT SHOULD NOT REPLACE THE EXPERTISE OF AN EXPERIENCED CRYSTALLOGRAPHIC REFEREE.

No syntax errors found. CIF dictionary Interpreting this report

Datablock: JinZhichaoPDW20200101_0m

```
Bond precision:  C-C = 0.0037 A         Wavelength=1.54178

Cell:           a=10.7298(6)    b=22.5657(13)      c=11.6473(7)
                alpha=90        beta=115.765(2)    gamma=90
Temperature:    273 K

                Calculated                 Reported
Volume          2539.8(3)                  2539.7(3)
Space group     P 21/n                     P 21/n
Hall group      -P 2yn                     -P 2yn
Moiety formula  C30 H23 Cl N2 O3           ?
Sum formula     C30 H23 Cl N2 O3           C30 H23 Cl N2 O3
Mr              494.95                     494.95
Dx,g cm-3       1.294                      1.294
Z               4                          4
Mu (mm-1)       1.608                      1.608
F000            1032.0                     1032.0
F000'           1036.33
h,k,lmax        11,24,12                   11,24,12
Nref            3523                       3512
Tmin,Tmax
Tmin'

Correction method= Not given

Data completeness= 0.997          Theta(max)= 57.893

R(reflections)= 0.0461( 3008)     wR2(reflections)= 0.1304( 3512)

S = 1.052                  Npar= 326
```

The following ALERTS were generated. Each ALERT has the format
test-name_ALERT_alert-type_alert-level.
Click on the hyperlinks for more details of the test.

Alert level A

```
THETM01_ALERT_3_A  The value of sine(theta_max)/wavelength is less than 0.550
            Calculated sin(theta_max)/wavelength =    0.5494
```

Alert level C

```
PLAT052_ALERT_1_C Info on Absorption Correction Method   Not Given     Please Do !
PLAT053_ALERT_1_C Minimum Crystal Dimension Missing (or Error) ...     Please Check
PLAT054_ALERT_1_C Medium  Crystal Dimension Missing (or Error) ...     Please Check
PLAT055_ALERT_1_C Maximum Crystal Dimension Missing (or Error) ...     Please Check
PLAT220_ALERT_2_C NonSolvent Resd 1  C   Ueq(max) / Ueq(min) Range        3.1 Ratio
PLAT242_ALERT_2_C Low    MainMol Ueq as Compared to Neighbors of        C27 Check
```

Alert level G

```
PLAT199_ALERT_1_G Reported _cell_measurement_temperature ..... (K)        273 Check
PLAT200_ALERT_1_G Reported   _diffrn_ambient_temperature ..... (K)        273 Check
PLAT793_ALERT_4_G Model has Chirality at C7          (Centro SPGR)          S Verify
PLAT793_ALERT_4_G Model has Chirality at C20         (Centro SPGR)          R Verify
PLAT883_ALERT_1_G No Info/Value for _atom_sites_solution_primary .     Please Do !
```

---

1 **ALERT level A** = Most likely a serious problem - resolve or explain
0 **ALERT level B** = A potentially serious problem, consider carefully
6 **ALERT level C** = Check. Ensure it is not caused by an omission or oversight
5 **ALERT level G** = General information/check it is not something unexpected

7 ALERT type 1 CIF construction/syntax error, inconsistent or missing data
2 ALERT type 2 Indicator that the structure model may be wrong or deficient
1 ALERT type 3 Indicator that the structure quality may be low
2 ALERT type 4 Improvement, methodology, query or suggestion
0 ALERT type 5 Informative message, check

It is advisable to attempt to resolve as many as possible of the alerts in all categories. Often the minor alerts point to easily fixed oversights, errors and omissions in your CIF or refinement strategy, so attention to these fine details can be worthwhile. In order to resolve some of the more serious problems it may be necessary to carry out additional measurements or structure refinements. However, the purpose of your study may justify the reported deviations and the more serious of these should normally be commented upon in the discussion or experimental section of a paper or in the "special_details" fields of the CIF. checkCIF was carefully designed to identify outliers and unusual parameters, but every test has its limitations and alerts that are not important in a particular case may appear. Conversely, the absence of alerts does not guarantee there are no aspects of the results needing attention. It is up to the individual to critically assess their own results and, if necessary, seek expert advice.

Publication of your CIF in IUCr journals

A basic structural check has been run on your CIF. These basic checks will be run on all CIFs submitted for publication in IUCr journals (*Acta Crystallographica*, *Journal of Applied Crystallography*, *Journal of Synchrotron Radiation*); however, if you intend to submit to *Acta Crystallographica Section C* or *E* or *IUCrData*, you should make sure that full publication checks are run on the final version of your CIF prior to submission.

Publication of your CIF in other journals

Please refer to the *Notes for Authors* of the relevant journal for any special instructions relating to CIF submission.

Validation response form

Please find below a validation response form (VRF) that can be filled in and pasted into your CIF.

```
# start Validation Reply Form
_vrf_THETM01_JinZhichaoPDW20200101_0m
;
PROBLEM: The value of sine(theta_max)/wavelength is less than 0.550
RESPONSE: ...
;
_vrf_PLAT052_JinZhichaoPDW20200101_0m
;
PROBLEM: Info on Absorption Correction Method   Not Given     Please Do !
RESPONSE: ...
;
_vrf_PLAT053_JinZhichaoPDW20200101_0m
;
PROBLEM: Minimum Crystal Dimension Missing (or Error) ...     Please Check
RESPONSE: ...
;
_vrf_PLAT054_JinZhichaoPDW20200101_0m
;
PROBLEM: Medium  Crystal Dimension Missing (or Error) ...     Please Check
RESPONSE: ...
;
_vrf_PLAT055_JinZhichaoPDW20200101_0m
;
PROBLEM: Maximum Crystal Dimension Missing (or Error) ...     Please Check
RESPONSE: ...
;
_vrf_PLAT220_JinZhichaoPDW20200101_0m
```

;
PROBLEM: NonSolvent Resd 1 C   Ueq(max) / Ueq(min) Range   3.1 Ratio
RESPONSE: ...
;
_vrf_PLAT242_JinZhichaoPDW20200101_0m
;
PROBLEM: Low   MainMol Ueq as Compared to Neighbors of   C27 Check
RESPONSE: ...
;
end Validation Reply Form

PLATON version of 22/12/2019; check.def file version of 13/12/2019

Datablock JinZhichaoPDW20200101_0m - ellipsoid plot

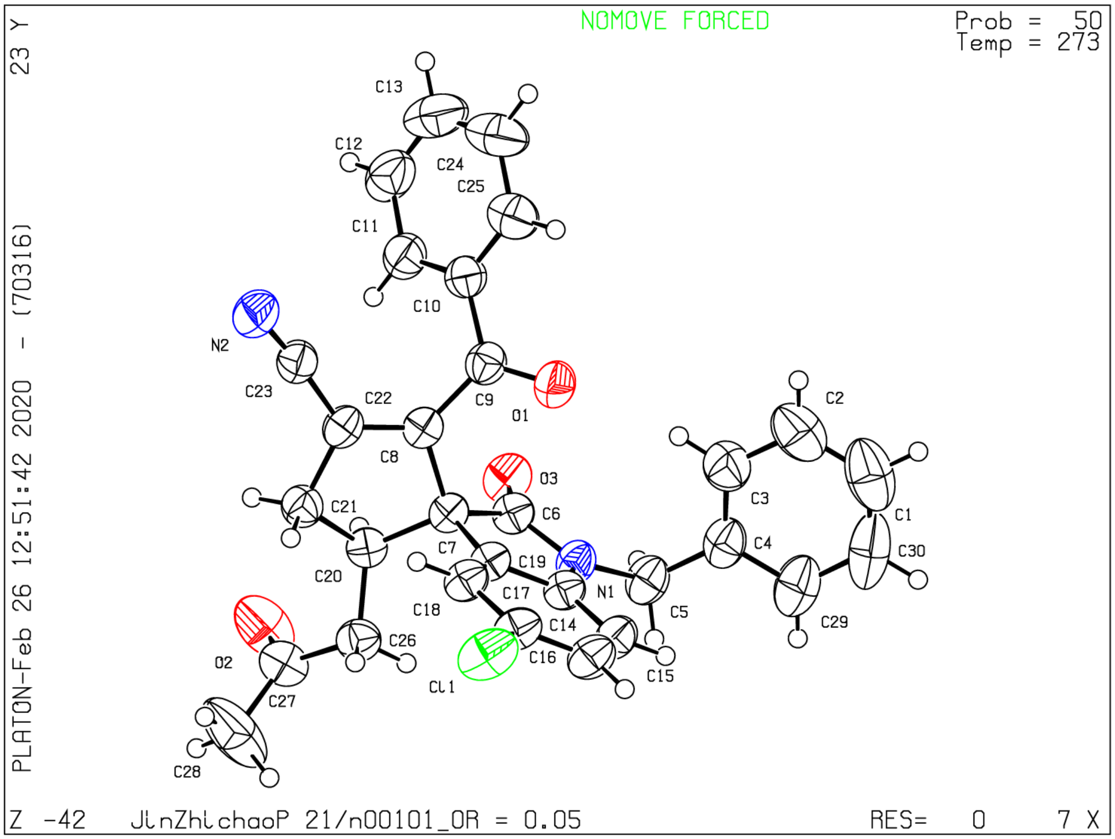

Supplement: Supplementary file 2 [file Data_Sheet_2.PDF]
