# Supplementary material for: Green and Facile Synthesis of Spirocyclopentanes Through NaOH-Promoted Chemo- and Diastereo-Selective (3 + 2) Cycloaddition Reactions of Activated Cyclopropanes and Enamides
Source: Front Chem. 2020 Jun 26;8:542. doi: 10.3389/fchem.2020.00542 (PMC7333539; doi:10.3389/fchem.2020.00542)
Supplement: Supplementary file 3 [file Data_Sheet_3.PDF]

Supplementary Material

1 Experiment Section

I: General information:

Commercially available materials purchased from J&K or Aladdin were used as received. THF was distilled over sodium and CH_2Cl_2 was distilled over CaH_2. Unless otherwise specified, all reactions were carried out under an atmosphere of air in 4 mL vial. Proton nuclear magnetic resonance (^1H NMR) spectra were recorded on a Bruker ASCEND (600 MHz) spectrometer. Chemical shifts were recorded in parts per million (ppm, δ) relative to tetramethylsilane (δ 0.00) or chloroform (δ = 7.26, singlet). ^1H NMR splitting patterns are designated as singlet (s), doublet (d), triplet (t), quartet (q), dd (doublet of doublets); m (multiplets), etc. All first-order splitting patterns were assigned on the basis of the appearance of the multiplet. Splitting patterns that could not be easily interpreted are designated as multiplet (m) or broad (br). Carbon nuclear magnetic resonance (^{13}C NMR) spectra were recorded on a Bruker ASCEND (151 MHz) spectrometer. Fluorine (^{19}F) nuclear magnetic resonance (^{19}F NMR) spectra were recorded on a Bruker (377 or 565 MHz) spectrometer. The melting points (m.p.) of the title compounds were determined when left untouched on an XT-4-MP apparatus from Beijing Tech. Instrument Co. (Beijing, China). High resolution mass spectral analysis (HR-MS) was performed on a quadrupole/electrostatic field orbitrap mass spectrometer. Relative configuration of the products was determined by X-ray crystallography. Analytical thin-layer chromatography (TLC) was carried out on Merck 60 F254 pre-coated silica gel plate (0.2 mm thickness). Visualization was performed using a UV lamp. Substrates **1f** [1] was prepared according to the reported literature procedure.

Ⅱ: Characterization of products:

Diethyl(1R,2S,5R)-2-benzoyl-1'-benzyl-5-(2-(4-nitrophenoxy)-2-oxoethyl)-2'-oxospiro[cyclopentane-1,3'-indoline]-3,3-dicarboxylate (**3a**)

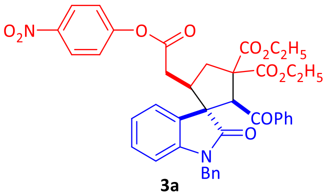

3a

White solid, m.p. 137-139 ℃, 31.7 mg, isolate yield 90%, > 20:1 dr (crude products mixture). ^1H NMR (600 MHz, $CDCl_3$) δ = 8.19 (d, *J* = 9.1 Hz, 2H), 7.54 (d, *J* = 7.3 Hz, 2H), 7.48 – 7.38 (m, 2H), 7.24 (d, *J* = 7.7 Hz, 2H), 7.16 (t, *J* = 7.3 Hz, 1H), 7.12 – 7.01 (m, 6H), 6.89 (d, *J* = 7.4 Hz, 2H), 6.41 (d, *J* = 7.4 Hz, 1H), 5.92 (s, 1H), 4.53 (dd, *J* = 51.6, 15.7 Hz, 2H), 4.42 – 4.30 (m, 2H), 4.27 (dq, *J* = 10.8 Hz, 7.2 Hz, 1H), 4.12 (dq, *J* = 10.7Hz, 7.2 Hz, 1H), 3.11 (dd, *J* = 6.0 Hz, 4.0 Hz, 2H), 2.96 (dd, *J* = 12.3, 8.9 Hz, 1H), 2.22 (ddd, *J* = 21.9 Hz, 16.7 Hz, 6.2 Hz, 2H), 1.31 (t, *J* = 7.1 Hz, 3H), 1.13 (t, *J* = 7.1 Hz, 3H). ^{13}C NMR (151 MHz, $CDCl_3$) δ = 196.4, 177.3, 171.1, 169.7, 168.7, 154.9, 145.4, 142.6, 136.8, 135.2, 133.1, 128.8, 128.6, 128.3, 127.49, 127.1, 125.18, 122.8, 122.2, 109.1, 62.6, 61.8, 61.6, 60.9, 59.0, 44.3, 43.7, 40.3, 34.4, 14.0, 13.8. HRMS (ESI, m/z):

Mass calcd. for $C_{40}H_{36}N_2O_{10}$ $[M+H]^+$ 705.2443, found 705.2443.

Diethyl(1R,2S,5R)-2-benzoyl-1'-benzyl-5'-methyl-5-(2-(4-nitrophenoxy)-2-oxoethyl)-2'-oxospiro[cyclopentane-1,3'-indoline]-3,3-dicarboxylate (3b)

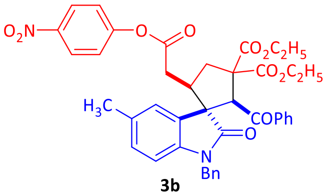

3b

White solid, m.p. 174-176 ℃, 32.0 mg, isolate yield 89%, > 20:1 dr (crude products mixture). ^{1}H NMR (600 MHz, $CDCl_3$) δ = 8.21 (d, J = 9.1 Hz, 2H), 7.54 (d, J = 7.5 Hz, 2H), 7.45 (t, J = 7.4 Hz, 1H), 7.25 (t, J = 7.8 Hz, 2H), 7.22 (s, 1H), 7.17 (d, J = 7.3 Hz, 1H), 7.15 – 7.07 (m, 4H), 6.93 (d, J = 7.4 Hz, 2H), 6.88 (d, J = 7.9 Hz, 1H), 6.31 (d, J = 8.0 Hz, 1H), 5.91 (s, 1H), 4.53 (dd, J = 59.3 Hz, 15.6 Hz, 2H), 4.45 – 4.33 (m, 2H), 4.32 – 4.25 (m, 1H), 4.19 – 4.08 (m, 1H), 3.15 – 3.05 (m, 2H), 2.98 (d, J = 7.5 Hz, 1H), 2.35 (s, 3H), 2.32 – 2.25 (m, 1H), 2.18 (dd, J = 16.7 Hz, 4.9 Hz, 1H), 1.34 (t, J = 7.1 Hz, 3H), 1.16 (t, J = 7.1 Hz, 3H). ^{13}C NMR (151 MHz, $CDCl_3$) δ = 196.4, 177.2, 171.0, 169.5, 168.7, 154.9, 145.3, 140.2, 136.9, 135.4, 133.0, 132.3, 129.1, 128.6, 128.3, 128.0, 127.4, 127.1, 125.2, 122.2, 108.7, 62.6, 61.7, 60.8, 59.1, 44.3, 43.6, 40.3, 34.4, 21.3, 14.0, 13.8. HRMS (ESI, m/z): Mass calcd. for $C_{41}H_{38}N_2O_{10}$ $[M+H]^+$ 719.2599, found 719.2582.

Diethyl(1R,2S,5R)-2-benzoyl-1'-benzyl-5'-methoxy-5-(2-(4-nitrophenoxy)-2-oxoethyl)-2'-oxospiro[cyclopentane-1,3'-indoline]-3,3-dicarboxylate (3c)

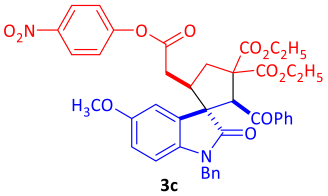

3c

White solid, m.p. 135-137 ℃, 23.9 mg, isolate yield 65%, > 20:1 dr (crude products mixture). ^{1}H NMR (600 MHz, $CDCl_3$) δ = 8.20 (d, J = 9.1 Hz, 2H), 7.61 (d, J = 7.5 Hz, 2H), 7.47 (t, J = 7.4 Hz, 1H), 7.29 (t, J = 7.8 Hz, 2H), 7.17- 7.07 (m, 6H), 6.85 (d, J = 7.6 Hz, 2H), 6.62 (dd, J = 8.6 Hz, 8.6 Hz, 1H), 6.32 (d, J = 8.6 Hz, 1H), 5.92 (s, 1H), 4.51 (s, 2H), 4.40-4.24 (m, 3H), 4.16-4.10 (m, 1H), 3.81 (s, 3H), 3.16-3.09 (m, 2H), 2.96 (dd, J = 13.5 Hz, 13.5 Hz, 1H), 2.32 - 2.25 (m, 1H), 2.19 (dd, J = 17 Hz, 17 Hz, 1H), 1.31 (t, J = 7.1 Hz, 3H), 1.14 (t, J = 7.1 Hz, 3H). ^{13}C NMR (151 MHz, $CDCl_3$) δ = 196.1, 176.7, 171.2, 169.7, 168.7, 155.8, 154.9, 145.3, 136.8, 135.8, 135.3, 133.2, 128.7, 128.4, 128.4, 127.4, 127.0, 126.3, 125.1, 122.3, 114.1, 113.8, 109.4, 62.7, 61.7, 61.2, 61.9, 58.7, 55.8, 44.3, 43.9, 40.1, 34.3, 14.0, 13.8. HRMS (ESI, m/z): calcd. for $C_{41}H_{39}N_2O_{11}$ $[M+H]^+$ 735.2548, found 735.2537.

Diethyl(1R,2S,5R)-2-benzoyl-1'-benzyl-5'-chloro-5-(2-(4-nitrophenoxy)-2-oxoethyl)-2'-oxospiro[cyclopentane-1,3'-indoline]-3,3-dicarboxylate (3d)

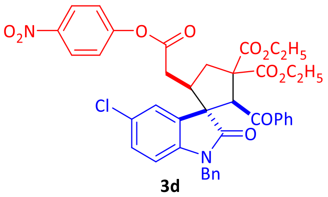

3d

White solid, m.p. 178-180 ℃, 27.4 mg, isolate yield 74%, > 20:1 dr (crude products mixture). ^{1}H NMR (600 MHz, $CDCl_3$) δ = 8.22- 8.18 (m, 2H), 7.62 (dd, *J* = 8.2 Hz, 8.5 Hz, 2H), 7.50-7.46 (m, 2H), 7.29 (t, *J* = 15.7 Hz, 2H), 7.18 - 7.04 (m, 3H), 6.84 (d, *J* = 7.1 Hz, 1H), 6.34 (d, *J* = 8.5 Hz, 1H), 5.89 (s, 1H), 4.54 (q, *J* = 15.7 Hz, 2H), 4.40-4.22 (m, 3H), 4.17-4.09 (m, 1H), 3.16-3.04 (m, 2H), 2.98-2.94 (m, 1H), 2.34 (dd, *J* = 16.6 Hz, 16.6 Hz, 1H), 2.19 (dd, *J* = 16.6 Hz, 5.8 Hz, 1H), 2.19 (dd, *J* = 16.5 Hz, 16.5 Hz, 1H), 1.31 (t, *J* = 7.1 Hz, 3H), 1.14 (t, *J* = 7.1 Hz, 3H). ^{13}C NMR (151 MHz, $CDCl_3$) δ = 195.7, 177.3, 171.2, 169.7, 168.8, 155.0, 145.4, 144.1, 142.5, 135.3, 134.3, 129.2, 128.8, 128.6, 128.6, 127.5, 127.5, 127.1, 125.3, 125.2, 122.8, 122.3, 109.1, 62.6, 61.8, 61.7, 61.0, 58.6, 44.3, 43.9, 40.4, 34.4, 21.2, 14.0, 13.8. HRMS (ESI, m/z): calcd. for $C_{40}H_{35}ClN_2O_{10}$ $[M+H]^+$ 739.2053, found 739.2048.

Diethyl(1R,2S,5R)-2-benzoyl-1'-benzyl-5'-bromo-5-(2-(4-nitrophenoxy)-2-oxoethyl)-2'-oxospiro[cyclopentane-1,3'-indoline]-3,3-dicarboxylate (3e)

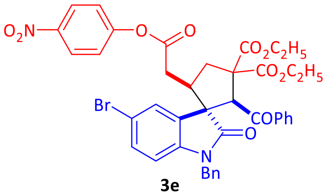

3e

White solid, m.p. 188-190 ℃, 34.9 mg, isolate yield 89%, > 20:1 dr (crude products mixture) ^{1}H NMR (600 MHz, $CDCl_3$) δ = 8.25 – 8.17 (m, 2H), 7.60 (dd, *J* = 16.1, 4.6 Hz, 3H), 7.47 (t, *J* = 7.4 Hz, 1H), 7.28 (t, *J* = 7.8 Hz, 2H), 7.20 (dd, *J* = 8.3 Hz, 1.9 Hz, 1H), 7.16 (t, *J* = 7.4 Hz, 1H), 7.13 – 7.05 (m, 4H), 6.85 (d, *J* = 7.4 Hz, 2H), 6.29 (d, *J* = 8.4 Hz, 1H), 5.87 (s, 1H), 4.54 (dd, *J* = 41.6, 15.7 Hz, 2H), 4.41 – 4.29 (m, 2H), 4.26 (ddd, *J* = 14.3 Hz, 9.0 Hz, 5.4 Hz, 1H), 4.16 – 4.10 (m, 1H), 3.09 (dt, *J* = 27.1, 9.8 Hz, 2H), 2.96 (dd, *J* = 13.4, 5.9 Hz, 1H), 2.35 (dd, *J* = 16.7, 8.0 Hz, 1H), 2.19 (dd, *J* = 16.7, 6.1 Hz, 1H), 1.31 (t, *J* = 7.1 Hz, 3H), 1.14 (t, *J* = 7.1 Hz, 3H). ^{13}C NMR (151 MHz, $CDCl_3$) δ = 196.0, 176.8, 170.9, 169.3, 168.5, 154.8, 145.4, 141.6, 134.7, 133.3, 131.7, 130.3, 128.7, 128.4, 127.6, 127.4, 127.0, 125.1, 122.2, 115.5, 110.4, 62.7, 62.0, 61.8, 60.9, 58.9, 44.3, 43.8, 40.2, 34.3, 14.0, 13.7. HRMS (ESI, m/z): calcd. for $C_{40}H_{35}BrN_2O_{10}$ $[M+H]^+$ 783.1548, found 783.1516.

Diethyl(1R,2S,5R)-2-benzoyl-1'-benzyl-5'-iodo-5-(2-(4-nitrophenoxy)-2-oxoethyl)-2'-oxospiro[cyclopentane-1,3'-indoline]-3,3-dicarboxylate (3f)

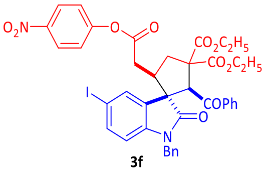

3f

White solid, m.p. 165-167 ℃, 37.4 mg, isolate yield 90%, > 20:1 dr (crude products mixture). ^{1}H NMR (600 MHz, $CDCl_3$) δ = 8.27 – 8.12 (m, 2H), 7.71 (d, *J* = 1.6 Hz, 1H),

7.63 – 7.54 (m, 2H), 7.46 (t, J = 7.4 Hz, 1H), 7.39 (dd, J = 8.3 Hz, 1.6 Hz, 1H), 7.29 – 7.24 (m, 4H), 7.16 (d, J = 7.3 Hz, 1H), 7.12 – 7.05 (m, 4H), 6.86 (d, J = 7.1 Hz, 2H), 6.18 (d, J = 8.3 Hz, 1H), 5.84 (s, 1H), 4.53 (q, J = 15.7 Hz, 2H), 4.42 – 4.19 (m, 3H), 4.19 – 4.06 (m, 1H), 3.04 (ddq, J = 23.1, 13.2, 6.5 Hz, 3H), 2.36 (dd, J = 16.7, 7.7 Hz, 1H), 2.20 (dd, J = 16.7, 6.1 Hz, 1H), 1.31 (t, J = 7.1 Hz, 3H), 1.15 (t, J = 7.1 Hz, 3H). ^{13}C NMR (151 MHz, $CDCl_3$) δ = 196.2, 176.8, 170.9, 169.3, 168.6, 154.9, 145.4, 142.4, 137.7, 136.8, 135.9, 134.8, 133.3, 128.8, 128.5, 127.8, 127.1, 125.2, 122.3, 111.0, 85.4, 62.8, 62.0, 60.6, 59.0, 44.3, 43.8, 40.3, 34.5, 14.0. HRMS (ESI, m/z): calcd. for $C_{40}H_{35}IN_2O_{10}$ $[M+H]^+$ 831.1409, found 831.1405.

Diethyl(1R,2S,5R)-2-benzoyl-1'-benzyl-5'-fluoro-5-(2-(4-nitrophenoxy)-2-oxoethyl)-2'-oxospiro[cyclopentane-1,3'-indoline]-3,3-dicarboxylate (3g)

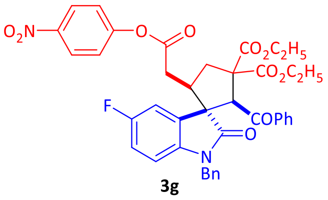

3g

White solid, m.p. 178-180 ℃, 27.8 mg, isolate yield 77%, > 20:1 dr (crude products mixture). ^{1}H NMR (600 MHz, $CDCl_3$) δ = 8.23 (d, J = 9.1 Hz, 2H), 7.67 (d, J = 7.4 Hz, 2H), 7.51 (s, 1H), 7.34 (d, J = 8.1 Hz, 3H), 7.14 (ddd, J = 26.0 Hz, 15.1 Hz, 7.4 Hz, 5H), 6.89 – 6.78 (m, 3H), 6.37 (dd, J = 8.6 Hz, 4.2 Hz, 1H), 5.95 (s, 1H), 4.56 (dd, J = 42.4 Hz, 15.7 Hz, 2H), 4.43 – 4.36 (m, 1H), 4.35 – 4.25 (m, 2H), 4.21 – 4.08 (m, 1H), 3.13 (ddd, J = 31.0, 19.3, 9.8 Hz, 2H), 2.97 (dd, J = 13.1 Hz, 5.5 Hz, 1H), 2.32 (dd, J = 16.6, 8.0 Hz, 1H), 2.20 (dd, J = 16.6 Hz, 5.7 Hz, 1H), 1.33 (t, J = 7.1 Hz, 3H), 1.16 (t, J = 7.1 Hz, 3H). ^{13}C NMR (151 MHz, $CDCl_3$) δ = 195.9, 176.8, 171.0, 169.5, 168.5, 159.6, 154.9, 145.4, 138.5, 136.7, 134.8, 133.4, 128.7, 128.5, 127.6, 127.0, 125.1, 122.2, 115.6, 115.4, 115.2, 109.6, 62.7, 62.0, 61.7 – 61.7, 61.6, 58.6, 44.4, 43.9, 40.2, 34.3, 14.0, 13.7. ^{19}F NMR (565 MHz, $CDCl_3$) δ = -118.77 (s). HRMS (ESI, m/z): calcd. for $C_{40}H_{35}FN_2O_{10}$ $[M+H]^+$ 723.2349, found 723.2349.

Diethyl(1R,2S,5R)-2-benzoyl-1'-benzyl-4'-chloro-5-(2-(4-nitrophenoxy)-2-oxoethyl)-2'-oxospiro[cyclopentane-1,3'-indoline]-3,3-dicarboxylate (3h)

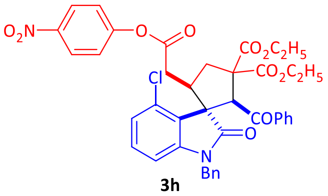

3h

White solid, m.p. 52-54 ℃, 36.7 mg, isolate yield > 99%, 4:1 dr (crude products mixture). ^{1}H NMR (600 MHz, $CDCl_3$) δ = 8.15 – 8.07 (m, 2H), 7.64 – 7.56 (m, 2H), 7.46 – 7.39 (m, 3H), 7.34 (t, J = 7.6 Hz, 2H), 7.21 (dd, J = 5.1 Hz, 2.0 Hz, 3H), 7.07 (t, J = 8.0 Hz, 1H), 6.99 (dd, J = 8.2 Hz, 0.8 Hz, 1H), 6.86 – 6.77 (m, 2H), 6.49 (dd, J = 7.8 Hz, 0.8 Hz, 1H), 5.80 (s, 1H), 4.87 (q, J = 15.9 Hz, 2H), 4.44 – 4.26 (m, 2H), 3.92 (dq, J = 10.7, 7.1 Hz, 1H), 3.79 (dq, J = 10.7, 7.1 Hz, 1H), 3.51 – 3.38 (m, 1H), 3.24 (dd, J = 12.6, 7.3 Hz, 1H), 2.98 – 2.82 (m, 2H), 2.77 – 2.63 (m, 1H), 1.27 (t, J = 7.2 Hz, 3H), 0.95 (t, J = 7.1 Hz, 3H). ^{13}C NMR (151 MHz, $CDCl_3$) δ = 197.6, 179.4, 171.4, 168.9, 168.4, 154.9, 146.9, 145.2, 138.8, 135.5, 131.9, 130.4, 128.6, 128.2, 127.9, 127.6, 127.4,

126.1, 124.9, 123.5, 122.2, 115.7, 108.3, 63.6, 62.4, 62.1, 61.6, 58.3, 45.0, 43.2, 42.6, 36.3, 13.8, 13.4. HRMS (ESI, m/z): calcd. for $C_{40}H_{36}ClN_2O_{10}$ $[M+H]^+$ 739.2053, found 739.2030.

Diethyl(1R,2S,5R)-2-benzoyl-1'-benzyl-4'-bromo-5-(2-(4-nitrophenoxy)-2-oxoethyl) -2'-oxospiro[cyclopentane-1,3'-indoline]-3,3-dicarboxylate (**3i**)

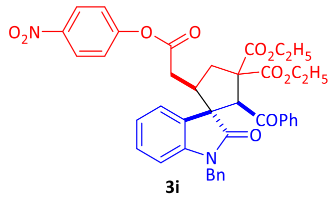

3i

Light yellow solid, m.p. 178-180 ℃, 39.0 mg, isolate yield > 99%, 7:1 dr (crude products mixture). ^{1}H NMR (600 MHz, $CDCl_3$) δ = 8.13 – 8.09 (m, 2H), 7.65 – 7.58 (m, 2H), 7.45 – 7.42 (m, 2H), 7.35 (t, *J* = 7.5 Hz, 2H), 7.21 (dd, *J* = 5.1 Hz, 1.9 Hz, 3H), 7.17 (dd, *J* = 8.2 Hz, 0.7 Hz, 1H), 6.99 (t, *J* = 8.0 Hz, 1H), 6.90 (d, *J* = 9.1 Hz, 1H), 6.85 – 6.76 (m, 2H), 6.53 (dd, *J* = 7.8 Hz, 0.7 Hz, 1H), 5.96 (s, 1H), 4.88 (s, 2H), 4.45 – 4.24 (m, 2H), 3.91 (dq, *J* = 10.7 Hz, 7.1 Hz, 1H), 3.78 (dq, *J* = 10.7 Hz, 7.1 Hz, 1H), 3.54 – 3.39 (m, 1H), 3.23 (dd, *J* = 12.5 Hz, 7.3 Hz, 1H), 3.01 (dd, *J* = 17.4 Hz, 9.7 Hz, 1H), 2.92 – 2.76 (m, 2H), 1.27 (t, *J* = 7.2 Hz, 4H), 0.94 (t, *J* = 7.1 Hz, 3H). ^{13}C NMR (151 MHz, $CDCl_3$) δ = 197.8, 179.6, 171.4, 168.9, 168.4, 161.9, 154.9, 147.2, 145.2, 138.8, 135.5, 131.9, 130.5, 128.6, 128.2, 127.9, 127.5, 126.8, 126.3, 126.1, 124.9, 122.2, 119.5, 115.7, 108.9, 63.6, 62.4, 62.1, 61.7, 58.6, 45.0, 43.3, 42.7, 36.2, 13.8, 13.1. HRMS (ESI, m/z): calcd. for $C_{40}H_{35}BrN_2O_{10}$ $[M+H]^+$ 783.1548, found 783.1555.

Diethyl(1R,2S,5R)-2-benzoyl-1'-benzyl-7'-fluoro-5-(2-(4-nitrophenoxy)-2-oxoethyl)-2'-oxospiro[cyclopentane-1,3'-indoline]-3,3-dicarboxylate (**3j**)

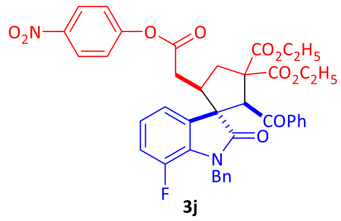

3j

White solid, m.p. 152-154 ℃, 31.8 mg, isolate yield 88%, > 20:1 dr (crude products mixture). ^{1}H NMR (600 MHz, $CDCl_3$) δ = 8.27- 8.17 (dt, *J* = 10.2 Hz, 10.1 Hz, 9.1 Hz, 2H), 7.54 (d, *J* = 7.2 Hz, 2H), 7.45 (t, *J* = 7.2 Hz, 1H), 7.25 – 7.23 (m, 3H), 7.17 – 7.08 (m, 5H), 7.04 – 6.99 (m, 3H), 6.89 (dd, *J* = 11.5 Hz, 11.5 Hz, 1H), 5.91 (s, 1H), 4.73 (d, *J* = 15.3 Hz, 1H), 4.64 (d, *J* = 15.3 Hz, 1H), 4.40 – 4.23 (m, 3H), 4.14 – 4.09 (m, 1H), 3.11 – 3.05 (m, 2H), 2.97 – 2.91 (m, 1H), 2.20 (dd, *J* = 16.4 Hz, 16.4 Hz, 1H), 2.09 (dd, *J* = 16.4 Hz, 16.4 Hz, 1H), 1.31 (t, *J* = 7.1 Hz, 3H), 1.12 (t, *J* = 7.1 Hz, 3H). ^{13}C NMR (151 MHz, $CDCl_3$) δ = 196.2, 177.0, 171.0, 169.5, 168.5, 154.8, 148.1, 146.4, 145.3, 136.6, 136.5, 133.3, 129.4, 129.3, 128.4, 128.4, 128.3, 127.5, 125.1, 123.4, 123.4, 123.3, 122.2, 117.0, 116.9, 62.7, 61.9, 61.6, 61.1, 59.0, 45.8, 45.8, 44.1, 40.2, 34.1, 29.7, 14.0, 13.7. ^{19}F NMR (377 MHz, $CDCl_3$) δ = -133.22 (s). HRMS (ESI, m/z): calcd. for $C_{40}H_{35}FN_2O_{10}$ $[M+H]^+$ 723.2349, found 723.2349.

Diethyl(1R,2S,5R)-2-benzoyl-1'-benzyl-7'-bromo-5-(2-(4-nitrophenoxy)-2-oxoethyl)

-2'-oxospiro[cyclopentane-1,3'-indoline]-3,3-dicarboxylate (3k)

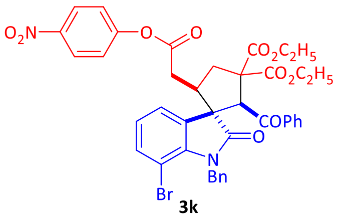

3k

White solid, m.p. 157-159 ℃, 28.2 mg, isolate yield 72%, > 20:1 dr (crude products mixture). ^{1}H NMR (600 MHz, $CDCl_3$) δ = 8.20 (d, *J* = 9.1 Hz, 2H), 7.56 (d, *J* = 7.6 Hz, 2H), 7.50 (t, *J* = 7.4 Hz, 1H), 7.46 (d, *J* = 7.5 Hz, 1H), 7.31 (t, *J* = 7.2 Hz, 3H), 7.12 (t, *J* = 7.6 Hz, 3H), 7.07 (t, *J* = 7.6 Hz, 2H), 6.97 (t, *J* = 7.6 Hz, 2H), 6.86 (d, *J* = 7.5 Hz, 1H), 5.94 (s, 1H), 5.06 – 5.0 (m, 2H), 4.39 – 4.24 (m, 3H), 4.15 – 4.09 (m, 1H), 3.14- 3.07 (m, 2H), 2.97-2.91 (m, 1H), 2.28-2.24 (m, 1H), 2.16 (dd, *J* = 16.6 Hz, 16.6 Hz, 1H), 1.29 (t, *J* = 7.1 Hz, 3H), 1.13 (t, *J* = 7.1 Hz, 3H). ^{13}C NMR (151 MHz, $CDCl_3$) δ = 196.3, 178.2, 171.0, 169.5, 168.5, 154.8, 145.4, 140.1, 137.0, 136.7, 134.9, 133.4, 128.6, 128.5, 128.4, 126.9, 126.6, 126.3, 125.2, 124.0, 122.2, 102.5, 62.7, 62.0, 61.6, 60.5, 59.1, 45.1, 44.3, 40.3, 34.2, 14.0, 13.7. HRMS (ESI, m/z): calcd. for $C_{40}H_{35}BrN_2O_{10}$ $[M+H]^+$ 783.1458, found 783.1422.

Diethyl(1R,2S,5R)-2-benzoyl-1'-benzyl-7'-nitro-5-(2-(4-nitrophenoxy)-2-oxoethyl)-2'-oxospiro[cyclopentane-1,3'-indoline]-3,3-dicarboxylate (3l)

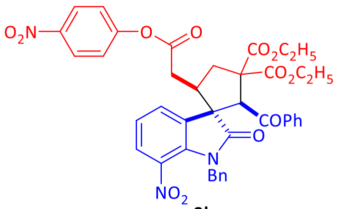

3l

Yellow solid, m.p. 170-172 ℃, 28.5 mg, isolate yield 76%, > 20:1 dr (crude products mixture). ^{1}H NMR (600 MHz, $CDCl_3$) δ = 8.28 – 8.22 (dt, *J* = 10 Hz, 9.9 Hz, 9.2 Hz, 2H), 7.79 – 7.78 (m, 3H), 7.56 (t, *J* = 7.4 Hz, 1H), 7.45 – 7.39 (m, 3H), 7.18 – 7.14 (m, 3H), 7.07 (t, *J* = 7.4 Hz, 1H), 6.93 (t, *J* = 7.7 Hz, 2H), 6.43 (d, *J* = 8.1 Hz, 2H), 6.11 (s, 1H), 5.10 (d, *J* = 15.3 Hz, 1H), 4.71 (d, *J* = 15.6 Hz, 1H), 4.42 – 4.22 (m, 3H), 4.14 – 4.08 (m, 1H), 3.23 – 3.14 (m, 2H), 2.95 (dd, *J* = 12 Hz, 12 Hz, 1H), 2.22 (d, *J* = 6.5 Hz, 2H), 1.31 (t, *J* = 7.1 Hz, 3H), 1.11 (t, *J* = 7.2 Hz, 3H). ^{13}C NMR (151 MHz, $CDCl_3$) δ = 195.8, 171.0, 169.5, 136.3, 134.9, 134.2, 133.6, 131.3, 129.0, 128.5, 128.7, 128.7, 127.6, 126.9, 125.3, 124.8, 122.6, 122.4, 62.3, 62.2, 61.7, 60.4, 58.0, 46.0, 44.8, 40.3, 34.0, 14.0, 13.7. HRMS (ESI, m/z): calcd. for $C_{40}H_{35}N_3O_{12}$ $[M+H]^+$ 750.2294, found 750.2272.

Diethyl(1R,2S,5R)-2-benzoyl-1'-benzyl-6'-chloro-5-(2-(4-nitrophenoxy)-2-oxoethyl)-2'-oxospiro[cyclopentane-1,3'-indoline]-3,3-dicarboxylate (3m)

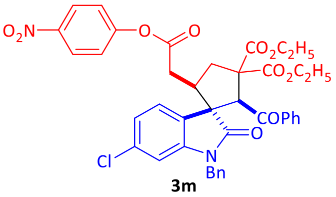

3m

White solid, m.p. 74-76 ℃, 29.2 mg, isolate yield 79%, > 20:1 dr (crude products mixture). ^{1}H NMR (600 MHz, $CDCl_3$) δ = 8.20 (d, *J* = 8.9 Hz, 2H), 7.62 (d, *J* = 7.8 Hz, 2H), 7.50 (t, *J* = 7.4 Hz, 1H), 7.41 (d, *J* = 8.8 Hz, 2H), 7.30 (t, *J* = 7.8 Hz, 1H), 7.17 (t, *J* = 7.4 Hz, 1H), 7.11 – 7.09 (m, 4H), 7.04 (dd, *J* = 8.1 Hz, 8.1 Hz, 1H), 6.85 (d, *J* = 7.5 Hz, 2H), 6.42 (d, *J* = 1.7 Hz, 1H), 5.92 (s, 1H), 4.51 (q, *J* = 15.7 Hz, 2H), 4.40 – 4.22 (m, 3H), 4.14 – 4.08 (m, 1H), 3.14 – 3.06 (m, 2H), 2.96 – 2.90 (m, 1H), 2.30 – 2.26 (m, 1H), 2.18 – 2.14 (m, 1H), 1.31 (t, *J* = 7.1 Hz, 3H), 1.12 (t, *J* = 7.1 Hz, 3H). ^{13}C NMR (151 MHz, $CDCl_3$) δ = 196.1, 177.2, 171.0, 169.5, 168.5, 154.8, 143.8, 136.6, 134.8, 134.4, 133.4, 128.8, 128.5, 128.4, 127.7, 127.0, 125.1, 123.6, 122.7, 122.2, 109.0, 62.7, 62.0, 61.6, 60.8, 58.1, 44.3, 43.5, 40.6, 34.3, 14.0, 13.7. HRMS (ESI, m/z): calcd. for $C_{40}H_{35}ClN_2O_{10}$ $[M+H]^+$ 739.2053, found 739.2048.

Diethyl(1R,2S,5R)-2-benzoyl-1'-benzyl-6'-bromo-5-(2-(4-nitrophenoxy)-2-oxoethyl)-2'-oxospiro[cyclopentane-1,3'-indoline]-3,3-dicarboxylate (3n)

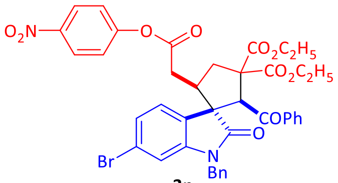

3n

White solid, m.p. 82-84 ℃, 34.9 mg, isolate yield 89%, > 20:1 dr (crude products mixture). ^{1}H NMR (600 MHz, $CDCl_3$) δ = 8.20 (dt, *J* = 3.1 Hz, 3.1 Hz, 3.1 Hz, 2H), 7.62 (d, *J* = 7.2 Hz, 2H), 7.49 – 7.47 (m, 2H), 7.29 (t, *J* = 7.9 Hz, 2H), 7.16 (t, *J* = 7.3 Hz, 1H), 7.12 – 7.08 (m, 4H), 7.06 (dd, *J* = 8.4 Hz, 8.4 Hz, 1H), 6.85 (d, *J* = 7.4 Hz, 2H), 6.34 (d, *J* = 8.4 Hz, 1H), 5.89 (s, 1H), 4.51 (dd, *J* = 41.4 Hz, 41.4 Hz, 2H), 4.40 – 4.24 (m, 3H), 4.16 – 4.10 (m, 1H), 3.16 – 3.10 (m, 1H), 3.07(t, *J* = 13.2 Hz，1H)，2.97 (dd, *J* = 13.2 Hz, 13.2 Hz, 1H), 2.34 (dd, *J* = 13.1 Hz, *J* = 13.1 Hz, 1H), 2.18 – 2.14 (m, 1H), 1.31 (t, *J* = 7.1 Hz, 3H), 1.14 (t, *J* = 7.3 Hz, 3H). ^{13}C NMR (151 MHz, $CDCl_3$) δ = 196.6, 176.7, 170.9, 169.7, 168.5, 154.9, 145.4, 141.1, 136.7, 134.7, 133.3, 128.8, 128.7, 128.5, 128.4, 128.2, 127.6, 127.0, 125.1, 122.2, 110.0, 62.7, 62.0, 61.8, 61.0, 58.8, 44.3, 43.8, 40.3, 34.3, 14.0, 13.9. HRMS (ESI, m/z): calcd. for $C_{40}H_{35}BrN_2O_{10}$ $[M+H]^+$ 783.1548, found 783.1537.

Diethyl(1R,2S,5R)-1'-benzyl-2-(2-methylbenzoyl)-5-(2-(4-nitrophenoxy)-2-oxoethyl)-2'-oxospiro[cyclopentane-1,3'-indoline]-3,3-dicarboxylate (4a)

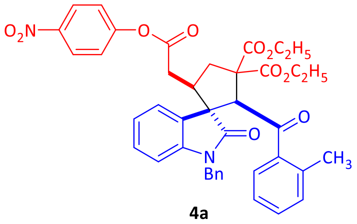

4a

White solid, m.p. 135-137 ℃, 33.1 mg, isolate yield 92%, > 20:1 dr (crude products mixture). ^{1} H NMR (600 MHz, $CDCl_3$) δ = 8.19 (d, *J* = 9.1 Hz, 2H), 7.63 (dd, *J* = 7.4 Hz, 0.9 Hz, 1H), 7.52 (d, *J* = 7.0 Hz, 1H), 7.29 (dd, *J* = 7.5 Hz, 1.1 Hz, 1H), 7.23 – 7.07 (m, 8H), 7.00 (d, *J* = 7.5 Hz, 1H), 6.86 (dd, *J* = 7.6 Hz, 1.7 Hz, 2H), 6.52 – 6.45 (m, 1H), 5.93 (s, 1H), 4.38 (dddd, *J* = 19.8 Hz, 14.3 Hz, 10.7 Hz, 9.7 Hz, 4H), 4.26 (d, *J* = 15.6 Hz, 1H), 4.22 – 4.08 (m, 1H), 3.19 – 2.91 (m, 3H), 2.13 (ddd, *J* = 21.5 Hz, 16.5 Hz, 6.6

Hz, 2H), 1.64 (s, 3H), 1.34 (t, J = 7.1 Hz, 3H), 1.20 (t, J = 7.2 Hz, 3H). ^{13}C NMR (151 MHz, $CDCl_3$) δ = 198.3, 176.9, 171.6, 170.2, 168.6, 154.9, 145.3, 142.7, 138.9, 136.6, 135.3, 131.6, 131.3, 129.4, 129.1, 128.7, 127.5, 127.0, 125.6– 124.9, 123.0, 122.3, 109.3, 62.6, 61.9, 61.5, 61.0, 44.3, 40.5, 34.06 (s), 19.6, 14.0, 13.8. HRMS (ESI, m/z): calcd. for $C_{41}H_{38}N_2O_{10}$ $[M+H]^+$ 719.2599, found 719.2582.

Diethyl(1R,2S,5R)-1'-benzyl-2-(2-bromobenzoyl)-5-(2-(4-nitrophenoxy)-2-oxoethyl) -2'-oxospiro[cyclopentane-1,3'-indoline]-3,3-dicarboxylate (**4b**)

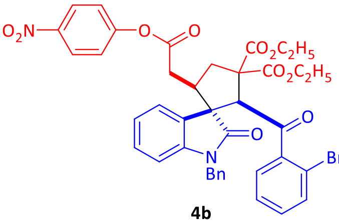

4b

White solid, m.p. 125-127 ℃, 33.3 mg, isolate yield 85%, > 20:1 dr (crude products mixture). ^{1}H NMR (600 MHz, $CDCl_3$) δ = 8.20 (J = 10.2 Hz, 10 Hz, 9.2 Hz, 2H), 7.65 (d, J = 6.9 Hz, 1H), 7.42 (dd, J = 7.9 Hz, 7.9 Hz, 1H), 7.25 – 7.10 (m, 9H), 7.03 – 7.00 (m, 2H), 6.96 (dd, J = 7.6 Hz, 7.6 Hz, 1H), 6.63 (d, J = 7.8 Hz, 1H), 6.01 (s, 1H), 4.57 (d, J = 15.6 Hz, 1H), 4.41 – 4.27 (m, 4H), 4.23 – 4.09 (m, 1H), 3.15 – 2.93 (m, 3H), 2.16 – 2.04 (m, 2H), 1.33 (t, J = 7.1 Hz, 3H), 1.22 (t, J = 7.1 Hz, 3H). ^{13}C NMR (151 MHz, $CDCl_3$) δ = 196.5, 176.4, 171.4, 169.9, 168.6, 154.9, 145.3, 142.9, 138.6, 135.4, 133.3, 132.2, 130.3, 129.2, 128.7, 127.6, 127.4, 127.3, 126.8, 125.3, 125.1, 123.5, 122.2, 120.0, 109.4, 62.6, 62.1, 61.5, 60.9, 60.9, 44.5, 44.4, 40.4, 33.9, 29.1, 14.0, 13.8. HRMS (ESI, m/z): calcd. for $C_{40}H_{35}BrN_2O_{10}$ $[M+H]^+$ 783.1548, found 783.1550.

Diethyl(1R,2S,5R)-1'-benzyl-2-(4-fluorobenzoyl)-5-(2-(4-nitrophenoxy)-2-oxoethyl)-2'-oxospiro[cyclopentane-1,3'-indoline]-3,3-dicarboxylate (**4c**)

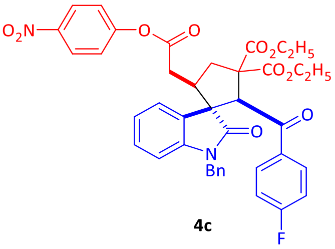

4c

White solid, m.p. 163-164 ℃, 31.8 mg, isolate yield 88%, > 20:1 dr (crude products mixture). ^{1}H NMR (600 MHz, $CDCl_3$) δ = 8.23 – 8.14 (dt, J = 10.2 Hz, 10.0 Hz, 9.2 Hz, 2H), 7.60 –7.58 (m, 2H), 7.43(d, J = 7.7 Hz, 1H), 7.20 – 7.04 (m, 7H), 6.93 – 6.87 (m, 4H), 6.50 (d, J = 7.7 Hz, 1H), 5.87 (s, 1H), 4.64 (d, J = 15.6 Hz, 1H), 4.54 (d, J = 15.6 Hz, 1H), 4.41 – 4.23 (m, 3H), 4.14 – 4.09 (m, 1H), 3.14 – 3.07 (m, 2H), 2.97 – 2.92 (m, 1H), 2.30 – 2.26 (m, 1H), 2.19 – 2.16 (m, 1H), 1.31 (t, J = 7.1 Hz, 3H), 1.13 (t, J = 7.1 Hz, 3H). ^{13}C NMR (151 MHz, $CDCl_3$) δ = 194.7, 177.2, 171.0, 169.5, 168.7, 166.2, 164.9, 154.4, 145.3, 142.5, 135.2, 133.1, 131.1, 131.0, 128.9, 128.8, 127.6, 127.4, 127.5, 125.1, 125.1, 122.8, 122.8, 115.3, 115.4, 109.0, 62.4, 61.9, 61.4, 60.9, 58.1, 44.2, 43.8, 40.9, 34.6, 14.3, 13.9. ^{19}F NMR (377 MHz, $CDCl_3$) δ = -104.34 (s). HRMS (ESI, m/z): calcd. for $C_{40}H_{35}FN_2O_{10}$ $[M]^+$ 722.2270, found 722.2296.

Diethyl(1R,2S,5R)-1'-benzyl-2-(4-chlorobenzoyl)-5-(2-(4-nitrophenoxy)-2-oxoethyl)

-2'-oxospiro[cyclopentane-1,3'-indoline]-3,3-dicarboxylate (4d)

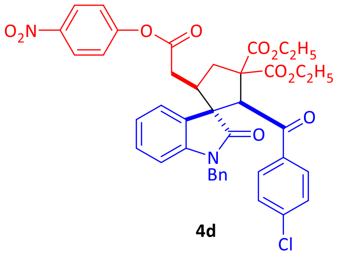

4d

White solid, m.p. 105-107 ℃, 32.5 mg, isolate yield 88%, > 20:1 dr (crude products mixture). ^{1}H NMR (600 MHz, $CDCl_3$) δ = 8.21 – 8.18 (m, 2H), 7.51 – 7.49 (m, 2H), 7.42 (d, J = 6.9 Hz, 1H), 7.21 – 7.04 (m, 9H), 6.91 (d, J = 7.3 Hz, 2H), 6.50 (d, J = 7.5 Hz, 1H), 5.86 (s, 1H), 4.65 (d, J = 15.6 Hz, 1H), 4.52 (d, J = 15.6 Hz, 1H), 4.41 – 4.22 (m, 3H), 4.14 – 4.08 (m, 1H), 3.13 – 3.08 (m, 2H), 2.97 – 2.91 (m, 1H), 2.29 – 2.25 (m, 1H), 2.19 – 2.15 (m, 1H), 1.31 (t, J = 7.1 Hz, 3H), 1.12 (t, J = 7.1 Hz, 3H). ^{13}C NMR (151 MHz, $CDCl_3$) δ = 195.1, 177.4, 171.0, 169.4, 168.8, 154.9, 145.8, 142.4, 139.8, 135.6, 135.6, 129.3, 128.9, 128.7, 128.1, 127.7, 127.0, 127.2, 125.6, 125.0, 122.7, 122.7, 109.7, 62.6, 61.3, 61.4, 60.1, 58.8, 44.3, 43.9, 40.8, 34.3, 14.0, 13.7. HRMS (ESI, m/z): calcd. for $C_{40}H_{35}ClN_2O_{10}$ $[M+Na]^+$ 7761.1872, found 761.1855.

Diethyl(1R,2S,5R)-1'-benzyl-2-(4-methylbenzoyl)-5-(2-(4-nitrophenoxy)-2-oxoethyl) -2'-oxospiro[cyclopentane-1,3'-indoline]-3,3-dicarboxylate (4e)

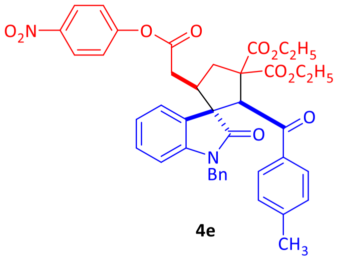

4e

White solid, m.p. 96-98 ℃, 34.9 mg, isolate yield 97%, > 20:1 dr (crude products mixture). ^{1}H NMR (600 MHz, $CDCl_3$) δ = 8.21 – 8.18 (m, 2H), 7.50 (d, J = 8.2 Hz, 2H), 7.47 – 7.40 (d, J = 7.4 Hz , 1H), 7.16 (t, J = 7.4 Hz, 1H), 7.10 – 7.03 (m, 8H), 6.87 (d, J = 7.4 Hz, 2H), 6.43 (d, J = 7.4 Hz, 1H), 5.91 (s, 1H), 4.62 (d, J = 15.7 Hz, 1H), 4.52 (d, J = 15.7 Hz, 1H), 4.40 – 4.22 (m, 3H), 4.14 – 4.08 (m, 1H), 3.15 – 3.08 (m, 2H), 2.97 – 2.90 (m, 1H), 2.34 (s, 3H), 2.29 – 2.25 (m, 1H), 2.16 (dd, J = 16.8 Hz, 16.8 Hz, 1H), 1.31 (t, J = 7.1 Hz, 3H), 1.11 (t, J = 7.1 Hz, 3H). ^{13}C NMR (151 MHz, $CDCl_3$) δ = 195.6, 177.7, 171.4, 169.6, 168.0, 154.7, 145.3, 144.0, 142.5, 135.6, 134.0, 129.1, 128.6, 128.1, 128.5, 127.5, 127.4, 127.1, 125.2, 125.1, 122.6, 122.9, 109.0, 62.6, 61.2, 61.7, 61.2, 58.2, 44.8, 43.5, 40.4, 34.4, 21.6, 14.3, 13.7. HRMS (ESI, m/z): calcd. for $C_{41}H_{38}N_2O_{10}$ $[M+H]^+$ 719.2599, found 719.2593.

Diethyl(1R,2S,5R)-1'-benzyl-2-(4-methoxybenzoyl)-5-(2-(4-nitrophenoxy)-2-oxoethyl)-2'-oxospiro[cyclopentane-1,3'-indoline]-3,3-dicarboxylate (4f)

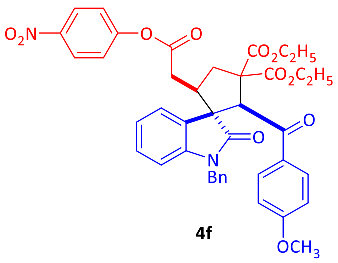

4f

White solid, m.p. 155-157 ℃, 32.3 mg, isolate yield 88%, > 20:1 dr (crude products mixture). ^{1}H NMR (600 MHz, $CDCl_3$) δ = 8.19 (dt, *J* = 10.2 Hz, 10.0 Hz, 9.2 Hz, 2H), 7.62 (d, *J* = 8.8 Hz, 2H), 7.46 (d, *J* = 7.2 Hz, 1H), 7.19 – 7.03 (m, 7H), 6.85 (d, *J* = 7.5 Hz, 2H), 6.73 (d, *J* = 8.9 Hz, 2H), 6.45 (d, *J* = 7.7 Hz, 1H), 5.90 (s, 1H), 4.74 (d, *J* = 15.7 Hz, 1H), 4.50 (d, *J* = 15.7 Hz, 1H), 4.40 – 4.21 (m, 3H), 4.14 – 4.08 (m, 1H), 3.79 (s, 3H), 3.16 – 3.08 (m, 2H), 2.93 (dd, *J* = 11.1 Hz, 11.1 Hz, 1H), 2.30 – 2.26 (m, 1H), 2.19 – 2.15 (m, 1H), 1.31 (t, *J* = 7.1 Hz, 3H), 1.11 (t, *J* = 7.1 Hz, 3H). ^{13}C NMR (151 MHz, $CDCl_3$) δ = 194.3, 177.3, 171.2, 169.6, 168.8, 163.7, 155.00, 145.3, 142.8, 135.2, 130.9, 129.9, 128.7, 128.6, 127.5, 127.4, 127.0, 125.2, 125.5, 122.6, 122.1, 113.6, 109.9, 62.1, 61.0, 61.6, 61.7, 58.6, 55.6, 44.6, 43.9, 40.3, 34.4, 14.4, 13.0. HRMS (ESI, m/z): calcd. for $C_{41}H_{38}N_2O_{11}$ $[M+H]^+$ 735.2548, found 735.2528.

Diethyl(1R,2S,5R)-1'-benzyl-2-(3-chlorobenzoyl)-5-(2-(4-nitrophenoxy)-2-oxoethyl) -2'-oxospiro[cyclopentane-1,3'-indoline]-3,3-dicarboxylate (**4g**)

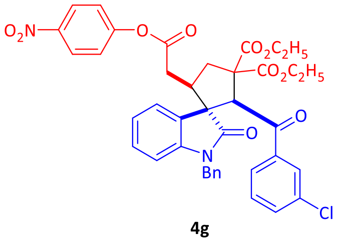

4g

White solid, m.p. 134-136 ℃, 31.0 mg, isolate yield 84%, > 20:1 dr (crude products mixture). ^{1}H NMR (600 MHz, $CDCl_3$) δ = 8.18 (dt, *J* = 10.2 Hz, 9.9 Hz, 9.2 Hz, 2H), 7.52 (d, *J* = 7.8 Hz, 1H), 7.42 – 7.39 (m, 3H), 7.19 – 7.05 (m, 8H), 6.95 (d, *J* = 7.2 Hz, 2H), 6.48 (d, *J* = 7.6 Hz, 1H), 5.83 (s, 1H), 4.64 (d, *J* = 15.6 Hz, 1H), 4.52 (d, *J* = 15.6 Hz, 1H), 4.41 – 4.24 (m, 3H), 4.15 – 4.10 (m, 1H), 3.14 – 3.08 (m, 2H), 2.99 – 2.93 (m, 1H), 2.30 – 2.26 (m, 1H), 2.20 – 2.16 (m, 1H), 1.32 (t, *J* = 7.1 Hz, 3H), 1.14 (t, *J* = 7.1 Hz, 3H). ^{13}C NMR (151 MHz, $CDCl_3$) δ = 195.4, 177.3, 170.6, 169.44, 168.6, 154.3, 145.3, 142.6, 138.3, 135.3, 134.7, 133.0, 129.3, 129.0, 128.6, 128.1, 127.6, 127.9, 127.0, 126.5, 125.1, 124.9, 122.9, 122.2, 109.1, 62.7, 62.0, 61.1, 60.4, 59.1, 44.4, 43.2, 40.2, 34.3, 14.3, 13.8. HRMS (ESI, m/z): calcd. for $C_{40}H_{35}ClN_2O_{10}$ $[M+H]^+$ 739.2053, found 739.2029.

Diethyl(1R,2S,5R)-1'-benzyl-2-(3-bromobenzoyl)-5-(2-(4-nitrophenoxy)-2-oxoethyl) -2'-oxospiro[cyclopentane-1,3'-indoline]-3,3-dicarboxylate (**4h**)

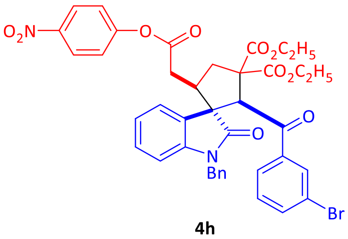

4h

White solid, m.p. 158-160 ℃, 35.3 mg, isolate yield 90%, > 20:1 dr (crude products mixture). ^{1}H NMR (600 MHz, $CDCl_3$) δ = 8.18 (J = 10.3 Hz, 10.0 Hz, 9.2 Hz, 2H), 7.57 – 7.52 (m, 3H), 7.41 (d, J = 7.4 Hz, 1H), 7.19 – 7.05 (m, 8H), 6.95 (d, J = 7.2 Hz, 2H), 6.48 (d, J = 7.8 Hz, 1H), 5.82 (s, 1H), 4.66 (d, J = 15.6 Hz, 1H), 4.49 (d, J = 15.6 Hz, 1H), 4.40 – 4.24 (m, 3H), 4.15 – 4.10 (m, 1H), 3.14 – 3.07 (m, 2H), 2.98 – 2.93 (m, 1H), 2.30 – 2.26 (m, 1H), 2.20 – 2.16 (m, 1H), 1.32 (t, J = 7.1 Hz, 3H), 1.15 (t, J = 7.1 Hz, 3H). ^{13}C NMR (151 MHz, $CDCl_3$) δ = 195.8, 177.2, 170.6, 169.4, 168.6, 154.2, 145.3, 142.6, 138.5, 135.9, 135.2, 131.0, 129.8, 129.0, 128.7, 127.9, 127.7, 127.2, 126.6, 125.5, 124.8, 122.9, 122.4, 122.6, 109.0, 62.79, 62.1, 61.7, 60.8, 59.4, 44.3, 43.6, 40.3, 34.5, 29.7, 14.3, 13.8. HRMS (ESI, m/z): calcd. for $C_{40}H_{35}BrN_2O_{10}$ $[M+H]^+$ 783.1548, found 783.1552.

Diethyl(1R,2S,5R)-1'-benzyl-2-(3-methylbenzoyl)-5-(2-(4-nitrophenoxy)-2-oxoethyl)-2'-oxospiro[cyclopentane-1,3'-indoline]-3,3-dicarboxylate (4i)

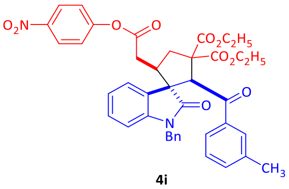

4i

White solid, m.p. 131-133 ℃, 30.9 mg, isolate yield 86%, > 20:1 dr (crude products mixture). ^{1}H NMR (600 MHz, $CDCl_3$) δ = 8.20 – 8.17 (m, 2H), 7.43 (d, J = 7.4 Hz, 1H), 7.37 (d, J = 7.8 Hz, 1H), 7.27 – 7.26 (m, 2H), 7.16 – 7.03 (m, 8H), 6.88 (d, J = 7.5 Hz, 2H), 6.40 (d, J = 7.9 Hz, 1H), 5.91 (s, 1H), 4.60 (d, J = 15.8 Hz, 1H), 4.46 (d, J = 15.8 Hz, 1H), 4.41 – 4.25 (m, 3H), 4.15 – 4.10 (m, 1H), 3.14 – 3.07 (m, 2H), 2.95 (q, J = 13.9 Hz, 1H), 2.29 – 2.25 (m, 1H), 2.26 (s, 3H), 2.18 – 2.15 (m, 1H), 1.31 (t, J = 7.1 Hz, 3H), 1.14 (t, J = 7.1 Hz, 3H). ^{13}C NMR (151 MHz, $CDCl_3$) δ = 196.5, 177.2, 171.1, 169.7, 168.7, 154.9, 145.3, 142.6, 138.2, 136.9, 135.3, 133.9, 128.8, 128.7, 128.3, 128.2, 127.5, 127.3, 127.0, 125.6, 125.2, 125.1, 122.8, 122.9, 109.5, 62.6, 61.8, 61.6, 60.9, 59.2, 44.7, 43.6, 40.1, 34.9, 21.1, 14.4, 13.1. HRMS (ESI, m/z): calcd. for $C_{41}H_{38}N_2O_{10}$ $[M+H]^+$ 719.2599, found 719.2587.

Diethyl(1R,2S,5R)-1'-benzyl-5-(2-(4-nitrophenoxy)-2-oxoethyl)-2'-oxo-2-(thiophene-2-carbonyl)spiro[cyclopentane-1,3'-indoline]-3,3-dicarboxylate (4j)

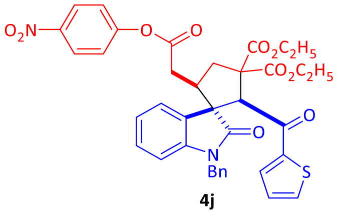

4j

White solid, m.p. 167-169 ℃, 30.9 mg, isolate yield 87%, > 20:1 dr (crude products

mixture). ^{1}H NMR (600 MHz, $CDCl_3$) δ = 8.23 (d, J = 9.1 Hz, 2H), 7.80 (d, J = 3.8 Hz, 1H), 7.56 (dd, J = 10.8, 6.2 Hz, 2H), 7.21 – 7.04 (m, 7H), 7.01 – 6.94 (m, 1H), 6.92 (d, J = 7.4 Hz, 2H), 6.56 (d, J = 7.7 Hz, 1H), 5.75 (s, 1H), 4.92 (d, J = 15.7 Hz, 1H), 4.55 (d, J = 15.7 Hz, 1H), 4.43 – 4.26 (m, 2H), 4.25 – 4.08 (m, 2H), 3.24 – 3.09 (m, 2H), 2.92 (dd, J = 12.7 Hz, 4.9 Hz, 1H), 2.31 (dd, J = 16.6 Hz, 7.9 Hz, 1H), 2.22 (dd, J = 16.6 Hz, 5.6 Hz, 1H), 1.32 (t, J = 7.1 Hz, 3H), 1.14 (t, J = 7.1 Hz, 3H). ^{13}C NMR (151 MHz, $CDCl_3$) δ = 187.9, 177.1, 171.0, 169.2, 168.7, 154.9, 145.3, 144.3, 142.3, 135.2, 133.7, 128.9, 128.6, 128.4, 127.5, 127.0, 125.1, 122.9, 122.3, 109.2, 62.6, 62.0, 61.7, 59.3, 44.2, 43.8, 40.1, 34.4, 14.0, 13.7. HRMS (ESI, m/z): calcd. for $C_{38}H_{34}N_2O_{10}S$ $[M+H]^+$ 711.2007, found 711.1993.

Diethyl(1R,2S,5R)-1'-benzyl-2-cinnamoyl-5-(2-(4-nitrophenoxy)-2-oxoethyl)-2'-oxospiro[cyclopentane-1,3'-indoline]-3,3-dicarboxylate (**4k**)

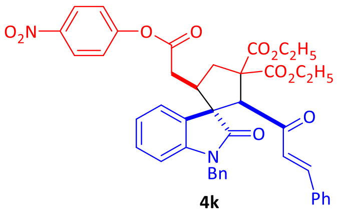

4k

White solid, m.p. 155-157 ℃, 32.9 mg, isolate yield 90%, > 20:1 dr (crude products mixture). ^{1}H NMR (600 MHz, $CDCl_3$) δ = 8.24 – 8.18 (m, 2H), 8.14 (d, J = 9.1 Hz, 1H), 7.47 (d, J = 6.8 Hz, 1H), 7.39 – 7.31 (m, 5H), 7.16 – 7.06 (m, 6H), 6.98 (t, J = 7.5 Hz, 2H), 6.90 (d, J = 9.2 Hz, 1H), 6.65 (d, J = 7.7 Hz, 1H), 6.39 (d, J = 16.1 Hz, 1H), 5.37 (s, 1H), 4.88 (d, J = 15.6 Hz, 1H), 4.65 (d, J = 15.6 Hz, 1H), 4.42 – 4.25 (m, 3H), 4.20 – 4.06 (m, 1H), 3.15 – 2.99 (m, 2H), 2.94 (t, J = 10.6 Hz, 1H), 2.27 – 2.10 (m, 2H), 1.32 (t, J = 7.1 Hz, 3H), 1.19 (t, J = 7.1 Hz, 3H). ^{13}C NMR (151 MHz, $CDCl_3$) δ = 194.4, 177.5, 171.2, 169.8, 168.7, 154.9, 145.3, 144.3, 142.9), 135.3), 134.1, 130.8 128.9, 128.6, 127.6, 127.2, 126.2, 125.4 – 125.0, 122.9, 122.2, 115.6, 109.3, 62.7, 62.0, 61.3, 60.9, 44.4, 43.9, 40.3, 34.2, 14.0, 13.8. HRMS (ESI, m/z): calcd. for $C_{42}H_{38}N_2O_{10}$ $[M+H]^+$ 731.2599, found 731.2588.

Diethyl(1R,2S,5R)-2-acetyl-1'-benzyl-5-(2-(4-nitrophenoxy)-2-oxoethyl)-2'-oxospiro[cyclopentane-1,3'-indoline]-3,3-dicarboxylate (**4l**)

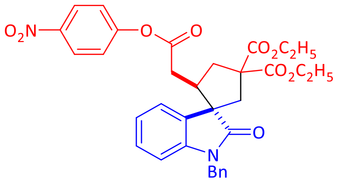

4l

White solid, m.p. 49-51 ℃, 23.1 mg, isolate yield 72%, > 20:1 dr (crude products mixture). ^{1}H NMR (600 MHz, $CDCl_3$) δ = 8.21 (d, J = 9.1 Hz, 2H), 7.35 (d, J = 6.3 Hz, 3H), 7.27 (dd, J = 12.4, 4.5 Hz, 6H), 7.10 (d, J = 9.2 Hz, 2H), 6.86 (d, J = 7.7 Hz, 1H), 5.01 (d, J = 15.4 Hz, 1H), 4.81 (d, J = 15.4 Hz, 1H), 4.78 (s, 1H), 4.41 – 4.18 (m, 4H), 3.05 – 2.82 (m, 3H), 2.15 (d, J = 6.7 Hz, 2H), 1.33 (d, J = 7.1 Hz, 3H), 1.25 (s, 3H). ^{13}C NMR (151 MHz, $CDCl_3$) δ = 202.4, 177.4, 171.0, 170.1, 168.7, 154.9, 145.3, 142.7, 135.6, 128.9, 127.9, 127.7, 126.4, 125.9, 125.1, 123.0, 122.2, 109.5, 65.1, 62.7, 62.1, 61.1, 59.7, 44.5, 43.9, 40.4, 34.1, 31.5, 14.0, 13.8. HRMS (ESI, m/z): calcd. for

$C_{35}H_{34}N_2O_{10}$ $[M+H]^+$ 643.2286, found 643.2288.

Triethyl(1R,2S,5R)-1'-benzyl-5-(2-(4-nitrophenoxy)-2-oxoethyl)-2'-oxospiro[cyclopentane-1,3'-indoline]-2,3,3-tricarboxylate (4m)

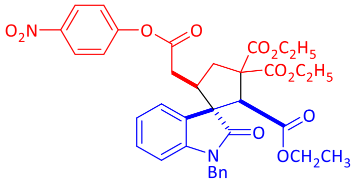

4m

Colorless oil, 25.2 mg, isolate yield 75%, > 20:1 dr (crude products mixture). ^{1}H NMR (600 MHz, $CDCl_3$) δ = 8.22 (d, *J* = 9.2 Hz, 2H), 7.62 – 7.56 (m, 1H), 7.37 (dd, *J* = 9.0, 2.3 Hz, 2H), 7.32 – 7.24 (m, 4H), 7.21 (dd, *J* = 7.8 Hz, 1.1 Hz, 1H), 7.16 – 7.10 (m, 2H), 7.04 (td, *J* = 7.6 Hz, 0.9 Hz, 1H), 6.80 (d, *J* = 7.7 Hz, 1H), 5.09 (d, *J* = 15.4 Hz, 1H), 4.84 (s, 1H), 4.69 (d, *J* = 15.4 Hz, 1H), 4.41 – 4.29 (m, 3H), 4.22 – 4.14 (m, 1H), 3.64 (dq, *J* = 10.8, 7.1 Hz, 1H), 3.50 (dq, *J* = 10.8, 7.1 Hz, 1H), 3.05 – 2.91 (m, 3H), 2.21 (t, *J* = 3.4 Hz, 2H), 1.32 (t, *J* = 7.1 Hz, 3H), 1.26 (d, *J* = 7.2 Hz, 3H), 0.41 (t, *J* = 7.1 Hz, 3H). ^{13}C NMR (151 MHz, $CDCl_3$) δ = 177.4 170.9, 170.0, 168.6, 168.4, 154.9, 145.3, 143.2, 135.7, 128.9, 128.7, 127.8, 126.5, 125.7, 125.1, 122.7, 122.3, 109.1, 62.6, 62.1, 60.9, 60.7, 60.3, 57.6, 44.4, 43.5, 40.8, 34.2, 13.9, 13.0. HRMS (ESI, m/z): calcd. for $C_{36}H_{36}N_2O_{11}$ $[M+H]^+$ 673.2392, found 673.2414.

Diethyl(1R,2S,5R)-2-benzoyl-1'-methyl-5-(2-(4-nitrophenoxy)-2-oxoethyl)-2'-oxospiro[cyclopentane-1,3'-indoline]-3,3-dicarboxylate (4n)

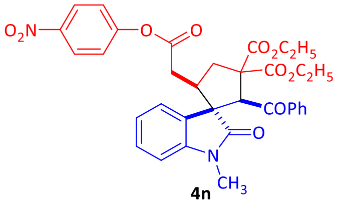

4n

White solid, m.p. 161-163 ℃, 24.2 mg, isolate yield 77%, > 20:1 dr (crude products mixture). ^{1}H NMR (600 MHz, $CDCl_3$) δ = 8.21 (d, *J* = 9.1 Hz, 2H), 7.39 (t, *J* = 7.3 Hz, 2H), 7.34 (d, *J* = 7.4 Hz, 2H), 7.20 (dt, *J* = 23.0 Hz, 7.6 Hz, 3H), 7.12 (d, *J* = 9.1 Hz, 2H), 7.07 (t, *J* = 7.5 Hz, 1H), 6.42 (d, *J* = 7.7 Hz, 1H), 5.82 (s, 1H), 4.44 – 4.26 (m, 3H), 4.20 – 4.08 (m, 1H), 3.05 (ddd, *J* = 16.9 Hz, 11.9 Hz, 9.8 Hz, 2H), 2.93 (dd, *J* = 11.8 Hz, 4.0 Hz, 1H), 2.75 (s, 3H), 2.29 (dd, *J* = 16.5 Hz, 7.7 Hz, 1H), 2.15 (dd, *J* = 16.4 Hz, 5.7 Hz, 1H), 1.32 (t, *J* = 7.1 Hz, 3H), 1.17 (t, *J* = 7.1 Hz, 3H). ^{13}C NMR (151 MHz, $CDCl_3$) δ = 196.4, 177.0, 171.1, 169.7, 168.6, 154.9, 145.3, 143.3, 136.9, 132.7, 128.9, 128.0, 127.0, 125.2 122.8, 122.3, 107.8, 62.6, 61.8, 61.0, 60.7, 60.1, 43.1, 40.6, 34.4, 26.2, 14.0, 13.8. HRMS (ESI, m/z): calcd. for $C_{34}H_{32}N_2O_{10}$ $[M+H]^+$ 629.2130, found 629.2134.

Diethyl(1R,2S,5R)-2-benzoyl-1'-benzyl-5-(2-(4-methoxyphenoxy)-2-oxoethyl)-2'-oxospiro[cyclopentane-1,3'-indoline]-3,3-dicarboxylate (4o)

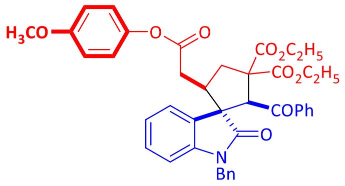

4o

White solid, m.p. 57-59 ℃, 29.7 mg, isolate yield 86%, > 20:1 dr (crude products mixture). ^{1}H NMR (600 MHz, $CDCl_3$) δ = 7.58 – 7.52 (m, 2H), 7.45 (dt, *J* = 5.9, 4.9 Hz, 2H), 7.26 (s, 4H), 7.18 – 7.09 (m, 3H), 7.09 – 7.03 (m, 2H), 6.86 (dd, *J* = 10.2 Hz, 4.1 Hz, 5H), 6.40 (dd, *J* = 6.5 Hz, 2.3 Hz, 1H), 5.97 (s, 1H), 4.63 – 4.48 (m, 2H), 4.43 – 4.22 (m, 3H), 4.19 – 4.08 (m, 1H), 3.78 (s, 3H), 3.16 – 2.93 (m, 3H), 2.19 – 2.02 (m, 2H), 1.31 (t, *J* = 7.1 Hz, 3H), 1.14 (t, *J* = 7.1 Hz, 3H). ^{13}C NMR (151 MHz, $CDCl_3$) δ = 196.4, 177.2, 171.2, 170.0, 169.7 157.2, 143.8, 142.4, 136.8, 135.4, 133.0, 128.6, 128.4, 127.4, 127.0, 125.4, 122.7, 122.1, 114.4, 109.0, 62.6, 61.7, 58.8, 55.5, 44.2, 40.3, 34.2, 14.0, 13.8. HRMS (ESI, m/z): calcd. for $C_{41}H_{39}NO_9$ $[M+H]^+$ 690.2698, found 690.2694.

Dsopropyl(1R,2S,5R)-2-benzoyl-1'-benzyl-5-(2-(4-nitrophenoxy)-2-oxoethyl)-2'-oxo spiro[cyclopentane-1,3'-indoline]-3,3-dicarboxylate (4p)

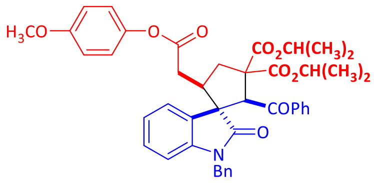

4p

White solid, m.p. 201-203 ℃, 28.9 mg, isolate yield 79%, > 20:1 dr (crude products mixture). ^{1}H NMR (600 MHz, $CDCl_3$) δ = 8.24 – 8.15 (m, 2H), 7.62 – 7.54 (m, 2H), 7.48 – 7.40 (m, 2H), 7.26 (dd, *J* = 6.0, 2.0 Hz, 2H), 7.19 – 7.00 (m, 7H), 6.88 (d, *J* = 7.1 Hz, 2H), 6.46 – 6.34 (m, 1H), 5.93 (s, 1H), 5.28 – 5.01 (m, 2H), 4.55 (s, 2H), 3.10 (dd, *J* = 5.9, 3.9 Hz, 2H), 2.93 (t, *J* = 10.5 Hz, 1H), 2.32 – 2.10 (m, 2H), 1.30 (dd, *J* = 6.3, 1.4 Hz, 6H), 1.20 (d, *J* = 6.2 Hz, 3H), 1.02 (d, *J* = 6.3 Hz, 3H). ^{13}C NMR (151 MHz, $CDCl_3$) δ = 196.1, 177.3, 170.5, 169.0, 168.7, 154.9, 145.3, 142.5, 136.8, 135.2, 133.1, 128.7, 128.4, 127.5, 127.1, 125.2, 122.7, 122.2, 109.0, 70.3, 69.6, 61.8, 60.9, 58.6, 44.2, 43.6, 40.4, 34.3, 21.7 – 21.3, 21.2. HRMS (ESI, m/z): calcd. for $C_{42}H_{40}N_2O_{10}$ $[M+H]^+$ 733.2756, found 733.2744.

(1S,5R)-2-benzoyl-1'-benzyl-5'-chloro-2'-oxo-5-(2-oxopropyl)spiro[cyclopentane-1,3'-indolin]-2-ene-3-carbonitrile (5a)

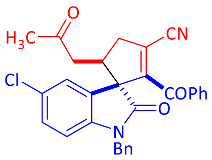

5a

White solid, m.p. 179- 181 ℃, 14.1 mg, isolate yield 57%, > 20:1 dr (crude products mixture). ^{1}H NMR (600 MHz, $CDCl_3$) δ = 7.87 – 7.74 (m, 2H), 7.74 – 7.61 (m, 1H), 7.51 (t, *J* = 7.7 Hz, 2H), 7.28 (dd, *J* = 7.2, 3.7 Hz, 6H), 7.19 (dd, *J* = 8.4, 2.1 Hz, 1H), 7.04 (d, *J* = 2.0 Hz, 1H), 6.63 (d, *J* = 8.4 Hz, 1H), 5.09 (d, *J* = 15.9 Hz, 1H), 4.76 (d, *J* = 15.9 Hz, 1H), 3.53 (ddd, *J* = 25.9, 16.8, 8.1 Hz, 2H), 2.80 (dd, *J* = 17.5, 9.3 Hz, 1H),

2.44 (dd, J = 10.3, 7.4 Hz, 2H), 1.93 (s, 3H). ^{13}C NMR (151 MHz, $CDCl_3$) δ = 205.2, 190.3, 176.1, 155.0, 141.8, 136.1, 135.0, 134.3, 129.5, 128.9, 127.9, 127.2, 124.2, 122.2, 113.4, 111.2, 65.2, 44.7, 44.0, 41.9 – 41.7, 41.6, 29.9. HRMS (ESI, m/z): calcd. for $C_{30}H_{23}ClN_2O_3$ $[M+H]^+$ 495.1470, found 495.1466.

(1S,5R)-2-benzoyl-1'-benzyl-5'-bromo-2'-oxo-5-(2-oxopropyl)spiro[cyclopentane-1, 3'-indolin]-2-ene-3-carbonitrile (5b)

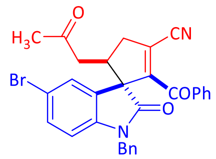

5b

White solid, m.p. 183-185 ℃, 18.6 mg, isolate yield 69%, > 20:1 dr (crude products mixture). ^{1}H NMR (600 MHz, $CDCl_3$) δ = 7.82 – 7.73 (m, 2H), 7.67 – 7.60 (m, 1H), 7.50 (dd, J = 10.6 Hz, 4.8 Hz, 2H), 7.33 (dd, J = 8.4 Hz, 2.0 Hz, 1H), 7.30 – 7.26 (m, 6H), 7.16 (d, J = 1.9 Hz, 1H), 6.57 (d, J = 8.4 Hz, 1H), 5.07 (d, J = 15.9 Hz, 1H), 4.75 (d, J = 15.9 Hz, 1H), 3.59 (t, J = 8.1 Hz, 1H), 3.45 (dd, J = 17.5 Hz, 8.4 Hz, 1H), 2.79 (dd, J = 17.5, 9.2 Hz, 1H), 2.49 – 2.33 (m, 2H), 1.92 (s, 3H). ^{13}C NMR (151 MHz, $CDCl_3$) δ = 205.2, 190.3, 176.0, 154.9, 142.3, 136.1, 134.9, 134.3, 132.4, 129.4, 129.2, 128.9, 127.8, 127.2, 126.8, 122.2, 115.2, 113.4, 111.7, 65.2, 44.6, 44.0, 41.7, 41.5, 29.8. HRMS (ESI, m/z): calcd. for $C_{30}H_{23}BrN_2O_3$ $[M+H]^+$ 539.0985, found 539.0972.

(1S,5R)-2-benzoyl-1'-benzyl-5'-methyl-2'-oxo-5-(2-oxopropyl)spiro[cyclopentane-1, 3'-indolin]-2-ene-3-carbonitrile (5c)

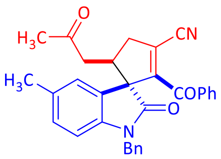

5c

Yellow solid, m.p. 207-209 ℃, 18.3 mg, isolate yield 77%, > 20:1 dr (crude products mixture). ^{1}H NMR (600 MHz, $CDCl_3$) δ = 7.78 (dd, J = 5.1, 3.3 Hz, 2H), 7.66 – 7.56 (m, 1H), 7.48 (dd, J = 10.6, 4.8 Hz, 2H), 7.29 – 7.23 (m, 6H), 7.01 (dd, J = 8.0 Hz, 0.8 Hz, 1H), 6.87 (s, 1H), 6.59 (d, J = 8.0 Hz, 1H), 5.02 (d, J = 15.8 Hz, 1H), 4.76 (d, J = 15.8 Hz, 1H), 3.66 – 3.54 (m, 1H), 3.44 (dd, J = 17.3, 8.4 Hz, 1H), 2.79 (dd, J = 17.4 Hz, 9.4 Hz, 1H), 2.37 (d, J = 7.7 Hz, 2H), 2.31 (s, 3H), 1.89 (s, 3H). ^{13}C NMR (151 MHz, $CDCl_3$) δ = 205.4, 190.4, 176.2, 155.7, 140.6, 136.2, 135.5, 134.1, 132.2, 129.9, 129.4, 128.7, 127.5, 127.1, 124.6, 121.4, 113.6, 110.0, 65.4, 44.4, 43.9, 41.9, 41.3, 29.8, 21.2. HRMS (ESI, m/z): calcd. for $C_{31}H_{26}N_2O_3$ $[M+H]^+$ 475.2016, found 475.2012.

(1S,5R)-2-benzoyl-1'-benzyl-5'-methyl-2'-oxo-5-(2-oxopropyl)spiro[cyclopentane-1, 3'-indolin]-2-ene-3-carbonitrile (5d)

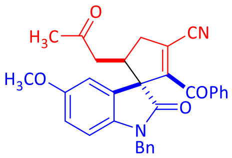

5d

White solid, m.p. 204-206 ℃, 16.4 mg, isolate yield 67%, > 20:1 dr (crude products mixture). ^{1}H NMR (600 MHz, $CDCl_3$) δ = 7.78 (dd, *J* = 5.1, 3.3 Hz, 2H), 7.64 – 7.57 (m, 1H), 7.48 (dd, *J* = 10.7, 4.7 Hz, 2H), 7.30 – 7.21 (m, 6H), 6.75 – 6.69 (m, 1H), 6.67 (d, *J* = 2.4 Hz, 1H), 6.59 (d, *J* = 8.5 Hz, 1H), 5.03 (d, *J* = 15.8 Hz, 1H), 4.76 (d, *J* = 15.8 Hz, 1H), 3.74 (s, 3H), 3.65 – 3.49 (m, 1H), 3.44 (dd, *J* = 17.4 Hz, 8.4 Hz, 1H), 2.77 (dd, *J* = 17.4, 9.2 Hz, 1H), 2.39 (dd, *J* = 7.4 Hz, 2.4 Hz, 2H), 1.90 (s, 3H). ^{13}C NMR (151 MHz, $CDCl_3$) δ = 205.4, 190.4, 176.0, 155.7, 155.5, 136.5, 136.2, 135.4, 134.1, 129.4, 128.7, 128.3, 127.6, 127.2, 121.7, 113.5, 112.9, 111.9, 110.5, 65.5, 55.7, 44.5, 43.9, 41.8, 41.3, 29.9. HRMS (ESI, m/z): calcd. for $C_{31}H_{26}N_2O_4$ $[M+H]^+$ 491.1965, found 491.1959.

(1S,5R)-2-benzoyl-1'-benzyl-6'-bromo-2'-oxo-5-(2-oxopropyl)spiro[cyclopentane-1,3'-indolin]-2-ene-3-carbonitrile (5e)

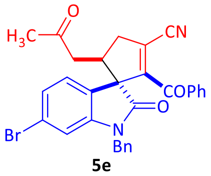

5e

Light yellow solid, m.p. 89-91 ℃, 18.1 mg, isolate yield 67%, > 20:1 dr (crude products mixture). ^{1}H NMR (600 MHz, $CDCl_3$) δ = 7.77 (dd, *J* = 8.3, 1.2 Hz, 2H), 7.63 (ddd, *J* = 8.7, 2.5, 1.2 Hz, 1H), 7.49 (t, *J* = 7.7 Hz, 2H), 7.31 – 7.24 (m, 6H), 7.17 (dd, *J* = 7.9, 1.7 Hz, 1H), 6.92 (d, *J* = 7.9 Hz, 1H), 6.84 (d, *J* = 1.7 Hz, 1H), 5.03 (d, *J* = 15.9 Hz, 1H), 4.75 (d, *J* = 15.9 Hz, 1H), 3.57 (dd, *J* = 15.9 Hz, 7.8 Hz, 1H), 3.45 (dd, *J* = 17.4 Hz, 8.5 Hz, 1H), 2.76 (dd, *J* = 17.4 Hz, 9.1 Hz, 1H), 2.39 (qd, *J* = 18.0 Hz, 7.4 Hz, 2H). ^{13}C NMR (151 MHz, $CDCl_3$) δ = 204.9, 190.0, 176.1, 154.7, 144.3, 135.8, 134.6, 134.1, 129.2, 128.6, 127.6, 126.9, 125.7, 125.2, 124.8, 123.0, 121.5, 113.2, 64.6, 44.4, 43.8, 41.2, 29.6. HRMS (ESI, m/z): calcd. for $C_{30}H_{23}BrN_2O_3$ $[M+H]^+$ 539.0965, found 539.0964.

diethyl(1R,2S,5R)-5-(2-(benzhydryloxy)-2-oxoethyl)-2-benzoyl-1'-benzyl-2'-oxospiro[cyclopentane-1,3'-indoline]-3,3-dicarboxylate (7)

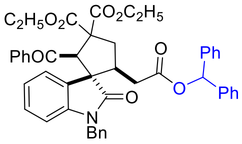

7

Light yellow oil, 16.5 mg, isolate yield 45%, > 20:1 dr (crude products mixture). ^{1}H NMR (400 MHz, $CDCl_3$) δ=7.56 – 7.49 (m, 2H), 7.46 – 7.36 (m, 2H), 7.34 – 7.19 (m, 11H), 7.14 (dd, J = 11.1 Hz, 4.6 Hz, 3H), 7.07 (dd, J = 10.0 Hz, 4.6 Hz, 2H), 7.03 – 6.98 (m, 2H), 6.84 (d, J = 7.2 Hz, 2H), 6.71 (s, 1H), 6.30 (dt, J = 5.5, 3.1 Hz, 1H), 5.93 (s, 1H), 4.44 (d, J = 1.8 Hz, 2H), 4.35 (ddd, J = 14.2 Hz, 8.9 Hz, 5.4 Hz, 1H), 4.24 (ddq, J = 14.2, 10.7, 7.1 Hz, 2H), 4.15 – 4.04 (m, 1H), 3.05 – 2.97 (m, 2H), 2.89 – 2.77 (m, 1H), 2.04 – 1.91 (m, 2H), 1.25 (d, J = 7.1 Hz, 3H), 1.15 – 1.08 (m, 3H). ^{13}C NMR (101 MHz, $CDCl_3$) δ=196.5, 177.2, 171.3, 170.1, 169.8, 142.2, 139.8, 136.8, 135.4, 133.0, 128.7 – 128.1, 127.9, 127.6 – 126.9, 126.1, 125.4, 122.6, 109.0, 77.5 – 76.9, 76.7, 62.5, 61.6, 61.1, 58.7, 44.1, 40.3, 34.3, 29.3, 13.8. HRMS (ESI, m/z): calcd. for $C_{47}H_{43}NO_8$ $[M+H]^+$ 772.2889, found 772.2886.

Ⅲ-a-1: General procedure for the preparation of D-A cyclopropyl acetates substrates:

The D–A cyclopropyl acetates substrates were prepared and characterized according to a known procedure [2], as briefed below:

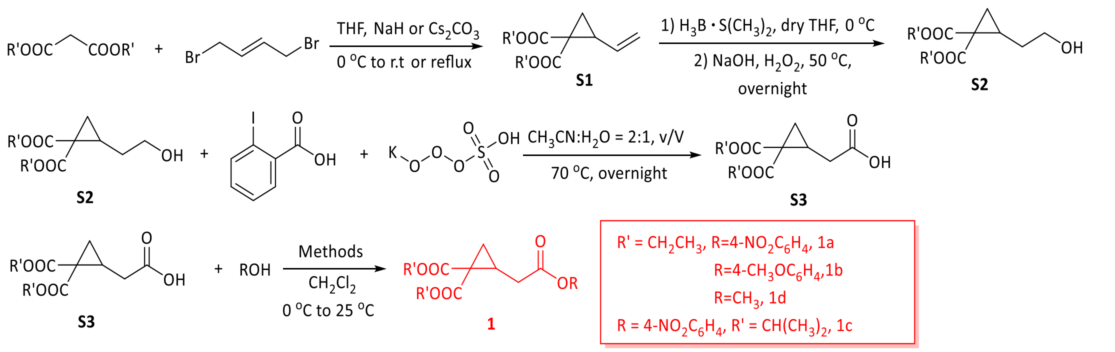

Supplementary Scheme S1. Preparation of substrates **1**

Step 1:

General procedure (1): To a solution of the corresponding malonate (40 mmol, 1.0 equivn.) and (*E*)-1,4-dibromobut-2-ene (40 mmol, 1.0 eq) in dry THF (151 mL) was added cesium carbonate (100 mmol, 2.5 equivn.). The crude products mixture was then heated at reflux overnight. After cooling down to room temperature, the crude products mixture was filtered over celite and washed with Et_2O. The organic phase was washed with saturated aq. $NaHCO_3$, water and brine, respectively. The organic phase was dried over anhydrous Na_2SO_4, filtered and the solvent was removed under reduced pressure. The crude product was used for the next step without further purification.

General procedure (2): To a 100 mL flame-dried round bottom flask was added NaH (44 mmol, 60% mineral dispersion) and anhydrous THF (60 mL) at 0 °C under N_2. Then a solution of malonate (20 mmol) in 10 mL dry THF was added dropwisely. After addition, the resulting crude products mixture was stirred for 2 h and then a solution of (*E*)-1,4-dibromobut-2-ene (20 mmol) in dry THF (10 mL) was added dropwisely. After addition, the reaction was stirred for 5 h at reflux and cooled down to room temperature. The solvent was removed under reduced pressure. The residue was dissolved by 100 mL EA and then washed with sat. Na_2CO_3. The organic phase was separated and the aqueous phase was extracted with EA. The organic phase was combined and dried over anhydrous $MgSO_4$, filtered and concentrated under vacuum. The crude products were purified by flash column chromatography with the mixture of EA and PE as eluent.

Step 2:

Borane dimethylsulfide complex (10.0 M in $S(CH_3)_2$) (42.2 mmol, 1.2 equivn.) was added to a solution of the corresponding vinylcyclopropane **S1** (35.2 mmol, 1.0 equivn.) in dry THF (0.53 M) at 0 ℃. The crude products mixture was stirred at 0 ℃ for 3 h. Then NaOH (3.0 M, 1.2 equivn., 42.2 mmol) was slowly added, followed by the addition of H_2O_2 (42.2 mmol, 1.2 equivn.). The mixture was then stirred at 50 ℃ overnight. The reaction was quenched with H_2O (35 mL) and extracted with EA. The organic extracts were combined and washed with brine. After filtration over anhydrous

Na_2SO_4, the solvent was removed under reduced pressure. Pure products were isolated after flash column chromatography on silica gel with the mixture of EA and PE as eluent.

Step 3:

To a solution of the alcohol substrate **S2** (1.0 equivn., 3.7 mmol) in acetonitrile: water (49 mL, 2: 1, v/v) was added a catalytic amount of oxidant 2IBAcid (0.2 equivn., 0.74 mmol) and Oxone (1.3 equivn., 4.8 mmol). The mixture was then stirred at 70 ℃ for 6 h and subsequently cooled in an ice bath to completely precipitate the insoluble hypervalent iodine by-product which was then removed by filtration. The precipitate was successively washed with cold water and cold dichloromethane and the combined filtrate was subsequently extracted with dichloromethane. The organic extract obtained from the work-up was dried over ($MgSO_4$) and evaporated to yield the carboxylic acid product.

Step 4:

General procedure (A): To a 100 mL round bottom flask equipped with a magnetic stir bar was added successively **S3** (1.0 equivn., 11.5 mmol), *p*-nitrophenol (1.0 equivn., 11.5 mmol), 35 mL dry CH_2Cl_2. The resulting mixture was cooled to 0 ℃ and added DCC (1.03 equivn., 11.8 mmol). The crude products mixture was then stirred at room temperature till complete consumption of **S3** (monitored by TLC). The crude products mixture was then cooled in an ice-bath and then filtered and concentrated under reduced pressure. Pure esters **1** were isolated after column chromatography on silica gel.

General procedure (B): To a 50 mL round bottom flask equipped with a magnetic stir bar was added successively **S3** (1.0 equivn., 2.6 mmol), 15 mL dry CH_2Cl_2, 1.6 mL oxalyl chloride (2.0 M in CH_2Cl_2, 1.2 equivn., 3.1 mmol), followed by 2 drops of dry DMF. The resulting mixture was stirred at room temperature for 11 h. Then, *p*-methoxyphenol or methanol (1.2 equivn., 3.1 mmol) was added, and the crude products mixture was cooled to 0 ℃ and pyridine (1.2 equivn., 3.1 mmol) was added slowly. The resulting crude products mixture was stirred at room temperature till complete consumption of **S3** (monitored by TLC). The crude products mixture was washed with water and separated. The inorganic phase was extracted with CH_2Cl_2 and the combined organic phase was dried over anhydrous $MgSO_4$, filtered and concentrated under reduced pressure. Pure esters **1** were isolated after column chromatography on silica gel.

Ⅲ-a-2: General procedure for the preparation of cyclopropyl aldehyde substrate:

The cyclopropyl aldehyde substrate **1e** were prepared and characterized according to a known procedure [3], as briefed below:

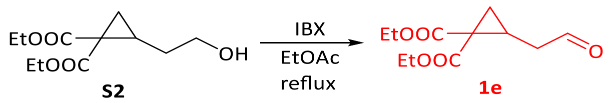

Supplementary Scheme S2. Preparation of **1e**

IBX (2.5 eq, 25 mmol) was added to a solution of the corresponding alcohol **S2** (1 eq, 10 mmol) in EtOAc (0.1 M). The crude products mixture was stirred at reflux for 3 h. Afterwards, the reaction was filtered through a celite pad. The solvent was removed under reduced pressure and pure products were isolated after flash column chromatography purification with PE: EA as the eluent.

Ⅲ-b: General procedure for the preparation of α,β-unsaturated ketone substrates:

The α,β-unsaturated ketone substrates were prepared and characterized according to a known procedure [4], as briefed below:

Method A:

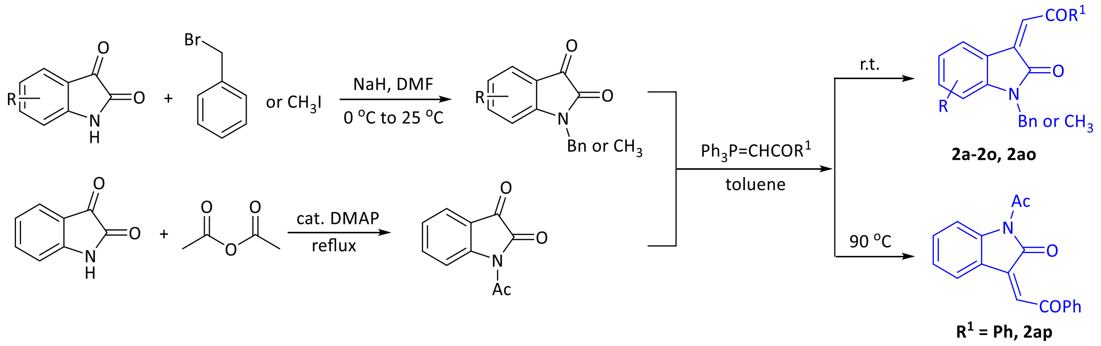

Supplementary Scheme S3. Preparation of the **2a-2o**，**2ao**，**2ap**

Step1-1: To a 250 mL round bottom flask isatin (2.94 g, 20 mmol) and DMF (37 mL) were added. The resulting bright orange solution was cooled to 0 ℃ by using an ice/water bath. NaH (60% dispersion in mineral oil, 840 mg, 21 mmol) was added portionwise to result in a deep purple solution. The solution was stirred until any effervescence had ceased (~15 min). Benzyl bromide (4.10 g, 2.85 mL, 24 mmol) or CH_3I (3.41 g, 1.49 mL, 24 mmol) was added in a dropwise manner and the resulting red-brown mixture was stirred for an additional 30 min at 0 ℃. H_2O (176 mL) was added to precipitate the product. The product was filtered and recrystallized from EtOH to afford the *N*-protected isatins.

Step1-2: Isatin (1 g, 6.98 mmol) and acetic anhydride (16 ml) was heated at 90–100 ℃ under nitrogen atmosphere for overnight. When isatin was consumed completely monitored by TLC analysis, the crude products mixture was allowed to keep in freeze for overnight to afford fine yellow crystal product.

Step2: Wittig reagent (1.58 mmol, 1.05 equiv) was added to a solution of the *N*-benzyl isatin or *N*-acetyl isatin (1.5 mmol, 1.0 equiv) in toluene (5 mL) in a 25 mL round bottom flask. The solution was stirred at rt or 90 ℃until the consumption of isatins was completed monitored by TLC analysis. The mixture was purified by column chromatography on SiO_2 to afford the final products **2a-2o**, **2ao**, **2ap**.

Method B:

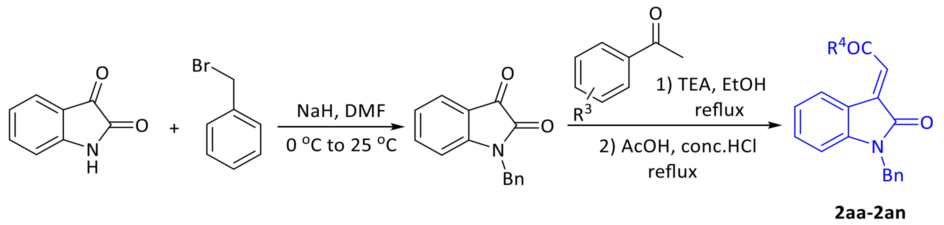

Supplementary Scheme S4. Preparation of the **2aa-2an**

Step1: To a 250 mL round bottom flask isatin (2.94 g, 20 mmol) and DMF (37 mL) were added. The resulting bright orange solution was cooled to 0 ℃ by using an ice/water bath. NaH (60% dispersion in mineral oil, 840 mg, 21 mmol) was added portionwise resulting in a deep purple solution. The solution was stirred until any effervescence had ceased (~15 min). Benzyl bromide (4.10 g, 2.85 mL, 24 mmol) was added in a dropwise manner and the resulting red-brown mixture was stirred for an additional 30 min at 0 ℃. H_2O (176 mL) was added to precipitate the product. The product was filtered and recrystallized from EtOH to afford the *N*-protected isatins.

Step2-1: To a warm solution of *N*-protected isatin (1.47 g, 10 mmol) in EtOH (30 mL), substituted acetophenone (10 mmol) and Et_3N (5 drops) were added. When the reaction was finished monitored by TLC, the crude products mixture was cooled to room temperature and the crystallized product was filtered and washed with cold EtOH.

Step2-2: A suspension of the above intermediates (2 mmol) in EtOH (8 mL) and cont. HCl (8 mL) was heated to boiling. Then the crude products mixture was cooled to room temperature when the material was consumed completely and the precipitate was filtered, washed with H_2O and cold EtOH, respectively, to afford the final products **2aa-2an**.

Ⅲ-c: General procedures for the base-catalyzed addition of D-A cyclopropanes to α,β-unsaturated ketones

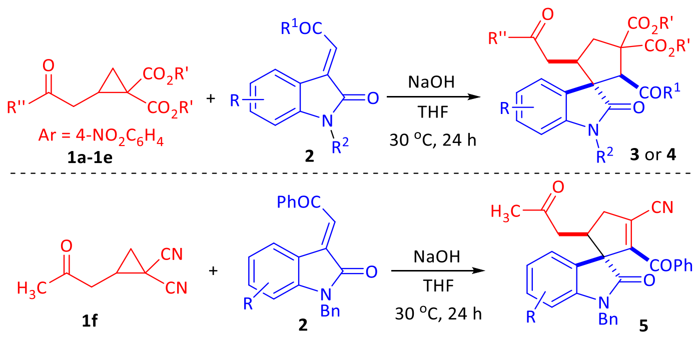

Supplementary Scheme S5. Preparation of the desired product **3**, **4** and **5**

To a 4 mL reaction vial equipped with a magnetic stir bar was added **2** (0.05 mmol), NaOH (0.01 mmol), esters or aldehyde **1a-1e** or 2-cyclopropyl ketone **1f** (0.10 mmol) and THF (1.0 mL). The mixture was stirred at 30 ℃ for 24 h. Then the solvent was removed under reduced pressure and the residue was purified via column chromatography to afford the products **3**, **4** or **5**.

Ⅲ-d: Synthetic Transformations of product 3a

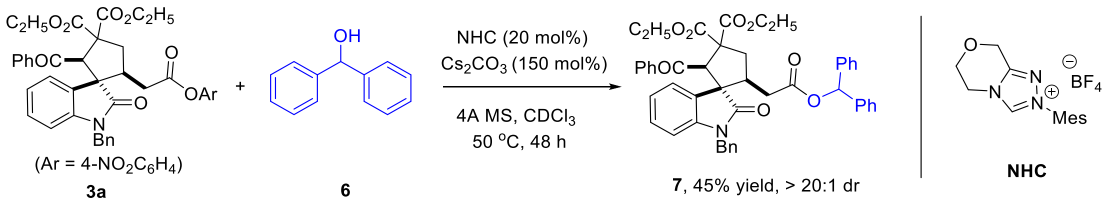

Supplementary Scheme S6. Synthetic Transformations of product **3a**

To a 2 mL reaction vial equipped with a magnetic stir bar was added **3a** (0.05 mmol), **6** (0.10 mmol) Cs_2CO_3 (0.075 mmol), NHC (0.01 mmol), 4Å MS (100 mg) and $CDCl_3$ (2.0 mL). The mixture was stirred at 50 ℃ for 48 h. Then the solvent was removed under reduced pressure and the residue was purified via column chromatography to afford the product **7**.

[1] Blom, J.; Vidal‐Albalat, A.; Jøgensen, J.; Barløe, C. L.; Jessen, K. S.; Iversen, M. V.; Jøgensen, K. A.; *Angew. Chem.* **2017**, *129*, 11993.

[2] Pan, D.; Mou, C.; Zan, N.; Lv, Y.; Song, B.-A.; Chi, Y. R.; Jin, Z. *Org. Lett*. **2019,** *21*, 6624.

[3] Sanchez-Diez, E.; Vesga, D. L.; Reyes, E.; Uria, U.; Carrillo, L.; Vicario, J. L. *Org. Lett*. **2016**, *16*, 1270.

[4] (a) Jensen, T.; Madsen, R. *J. Org. Chem.* **2009**, *74*, 3990; (b) Zhang, X. C.; Cao, S.-H.; Wei, Y.; Shi, M. *Chem. Commun.* **2011**, *47*, 1548; (c) Beccalli, E. M.; Marchesini, A. *Tetrahedron* **1993**, *49*, 474; (d) Tan, B.; Zeng, X. W. Y.; Leong, Shi,W. Z.; Barbas III, C. F.; Zhong, G. *Chem. Eur. J.* **2012**, *18*, 63.

3 Stereochemistry determination via X-ray crystallographic analysis

Relative configurations of the product **3** and **4** were assigned based on the crystal X-ray structures of **3e**. CCDC 1977165 (**3e**, obtained as colorless particles via evaporization of an EtOAc/Hex. solution) contains the supplementary X-ray crystallographic data.

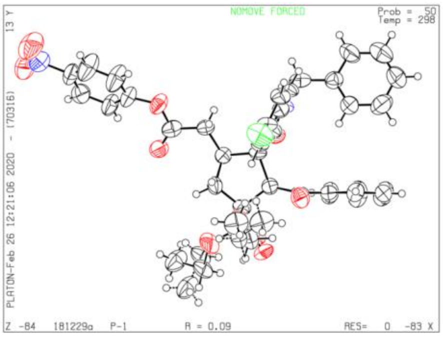

checkCIF/PLATON report

You have not supplied any structure factors. As a result the full set of tests cannot be run.

THIS REPORT IS FOR GUIDANCE ONLY. IF USED AS PART OF A REVIEW PROCEDURE FOR PUBLICATION, IT SHOULD NOT REPLACE THE EXPERTISE OF AN EXPERIENCED CRYSTALLOGRAPHIC REFEREE.

No syntax errors found. CIF dictionary Interpreting this report

Datablock: 181229a

```
Bond precision:  C-C = 0.0165 A          Wavelength=0.71073

Cell:           a=10.2560(9)      b=14.1850(12)     c=14.4350(13)
                alpha=62.399(1)   beta=79.724(2)    gamma=85.725(4)
Temperature:    298 K
```

	Calculated	Reported
Volume	1831.1(3)	1831.1(3)
Space group	P -1	P-1
Hall group	-P 1	?
Moiety formula	C40 H35 Br N2 O10	?
Sum formula	C40 H35 Br N2 O10	C40 H35 Br N2 O10
Mr	783.60	783.61
Dx,g cm-3	1.421	1.421
Z	2	2
Mu (mm-1)	1.185	1.185
F000	808.0	808.0
F000’	807.82	
h,k,lmax	12,16,17	12,16,17
Nref	6469	6362
Tmin,Tmax	0.892,0.942	0.789,0.943
Tmin’	0.780	

```
Correction method= # Reported T Limits: Tmin=0.789 Tmax=0.943
AbsCorr = MULTI-SCAN

Data completeness= 0.983            Theta(max)= 25.020

R(reflections)= 0.0931( 1941)       wR2(reflections)= 0.2259( 6362)

S = 1.062                  Npar= 520
```

Supplementary Figure S1. The crystal X-ray structures of **3e**

Relative configuration of the product **5** were assigned based on the crystal X-ray structures of **5a**. CCDC 1977149 (**5a**, obtained as colorless needle via evaporization of an EtOAc/Hex. solution) contains the supplementary X-ray crystallographic data.

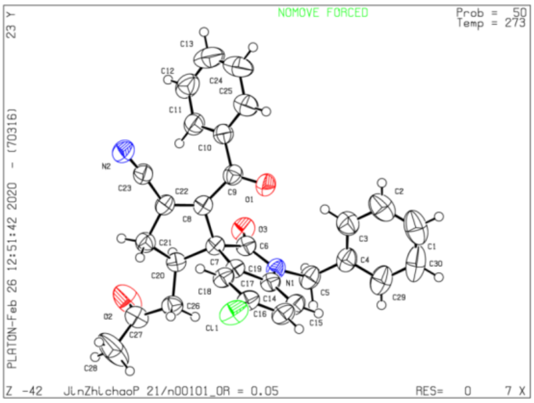

checkCIF/PLATON report

You have not supplied any structure factors. As a result the full set of tests cannot be run.

THIS REPORT IS FOR GUIDANCE ONLY. IF USED AS PART OF A REVIEW PROCEDURE FOR PUBLICATION, IT SHOULD NOT REPLACE THE EXPERTISE OF AN EXPERIENCED CRYSTALLOGRAPHIC REFEREE.

No syntax errors found. CIF dictionary Interpreting this report

Datablock: JinZhichaoPDW20200101_0m

```
Bond precision:  C-C = 0.0037 A          Wavelength=1.54178

Cell:            a=10.7298(6)     b=22.5657(13)       c=11.6473(7)
                 alpha=90         beta=115.765(2)     gamma=90
Temperature:     273 K

                 Calculated                Reported
Volume           2539.8(3)                 2539.7(3)
Space group      P 21/n                    P 21/n
Hall group       -P 2yn                    -P 2yn
Moiety formula   C30 H23 Cl N2 O3          ?
Sum formula      C30 H23 Cl N2 O3          C30 H23 Cl N2 O3
Mr               494.95                    494.95
Dx,g cm-3        1.294                     1.294
Z                4                         4
Mu (mm-1)        1.608                     1.608
F000             1032.0                    1032.0
F000'            1036.33
h,k,lmax         11,24,12                  11,24,12
Nref             3523                      3512
Tmin,Tmax
Tmin'

Correction method= Not given

Data completeness= 0.997          Theta(max)= 57.893

R(reflections)= 0.0461( 3008)     wR2(reflections)= 0.1304( 3512)

S = 1.052                 Npar= 326
```

Supplementary Figure S2. The crystal X-ray structures of **5a**

3 NMR Spectra for all new products

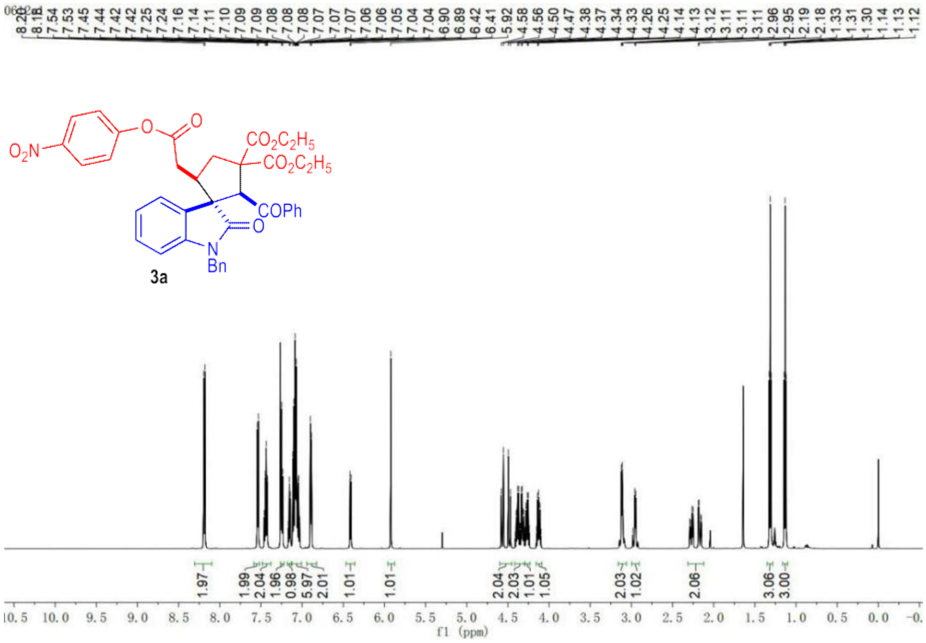

Supplementary Figure S3 ^{1}H NMR spectrum of **3a** (600 MHz, $CDCl_3$)

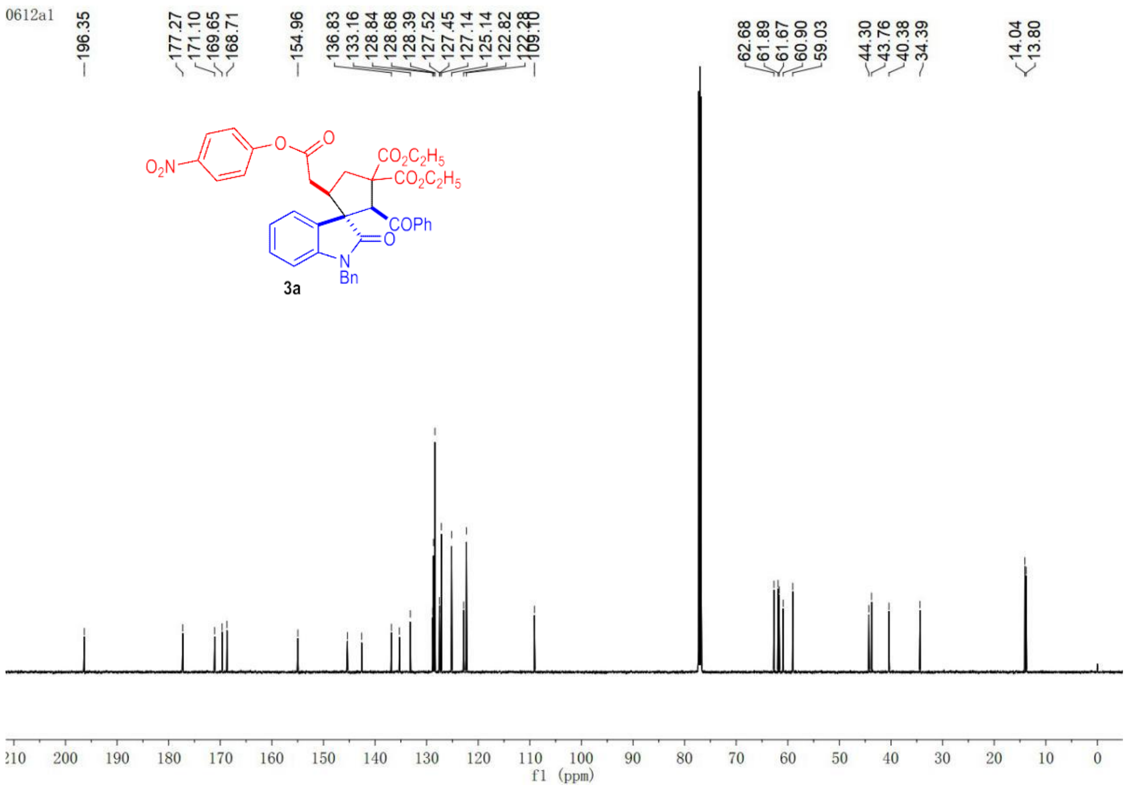

Supplementary Figure S4 ^{13}C NMR spectrum of **3a** (151 MHz, $CDCl_3$)

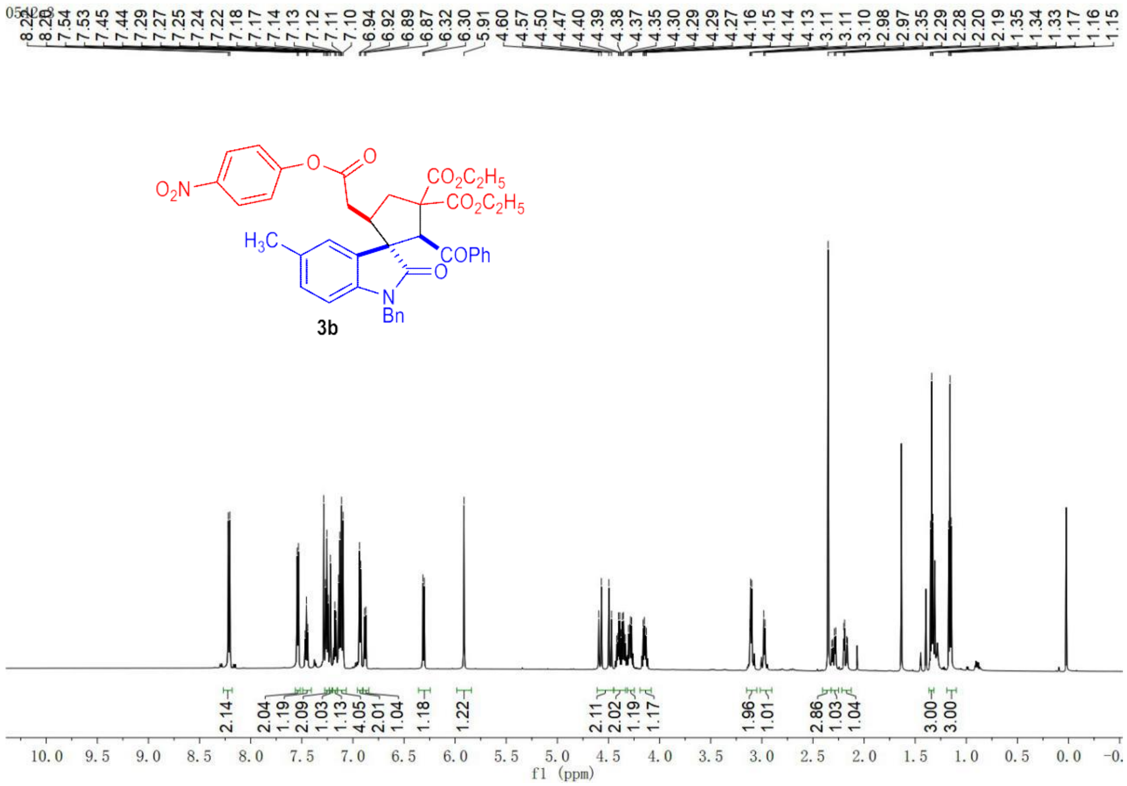

Supplementary Figure S5 ^{1}HNMRspectrumof **3b** (600MHz,CDCl$_3$)

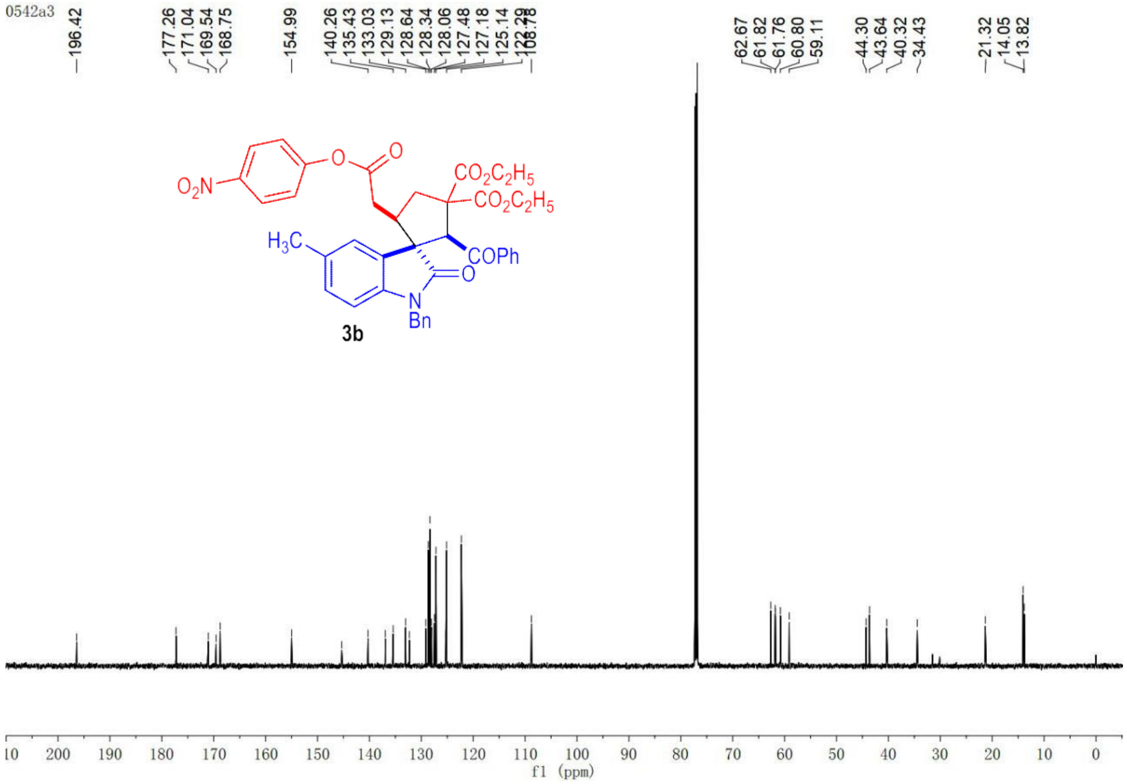

Supplementary Figure S6 ^{13}C NMR spectrum of **3b** (151 MHz, CDCl$_3$)

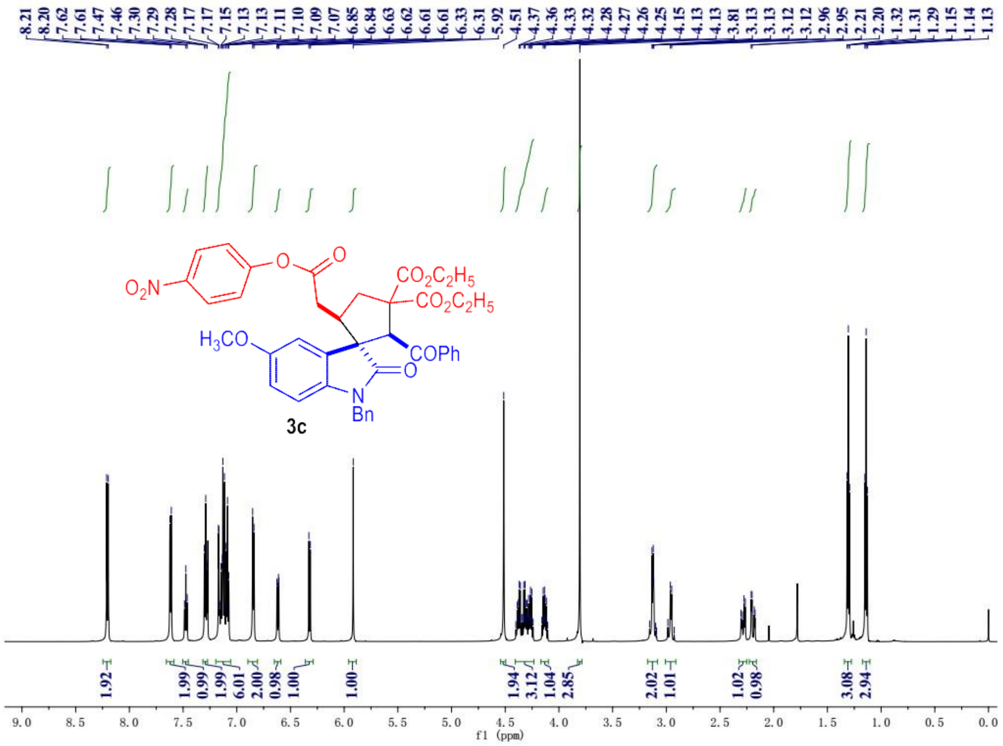

Supplementary Figure S7 ^{1}H NMR spectrum of **3c** (600 MHz, $CDCl_3$)

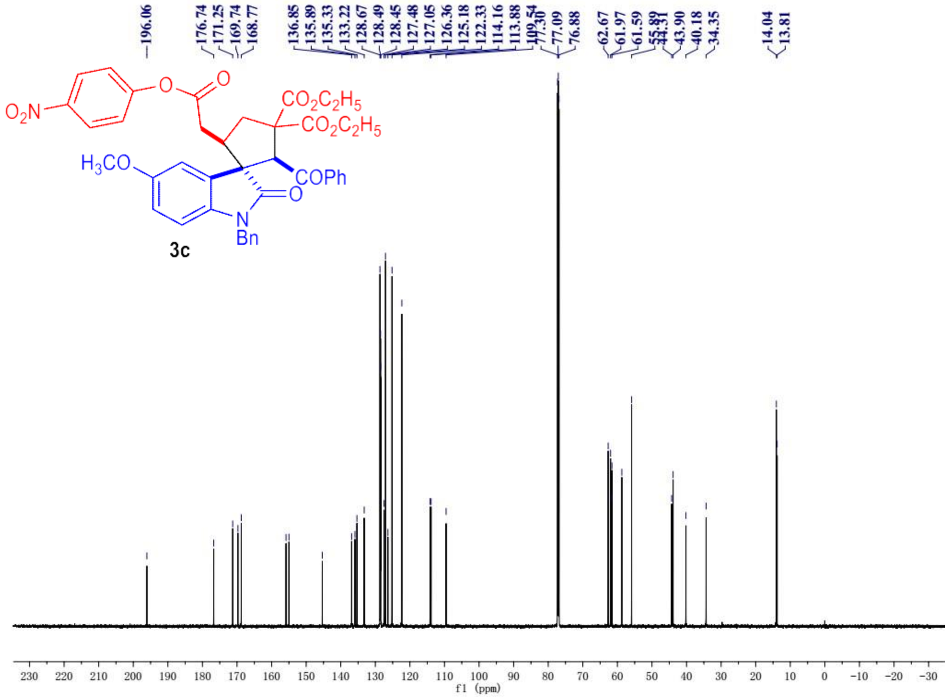

Supplementary Figure S8 ^{13}C NMR spectrum of **3c** (151 MHz, $CDCl_3$)

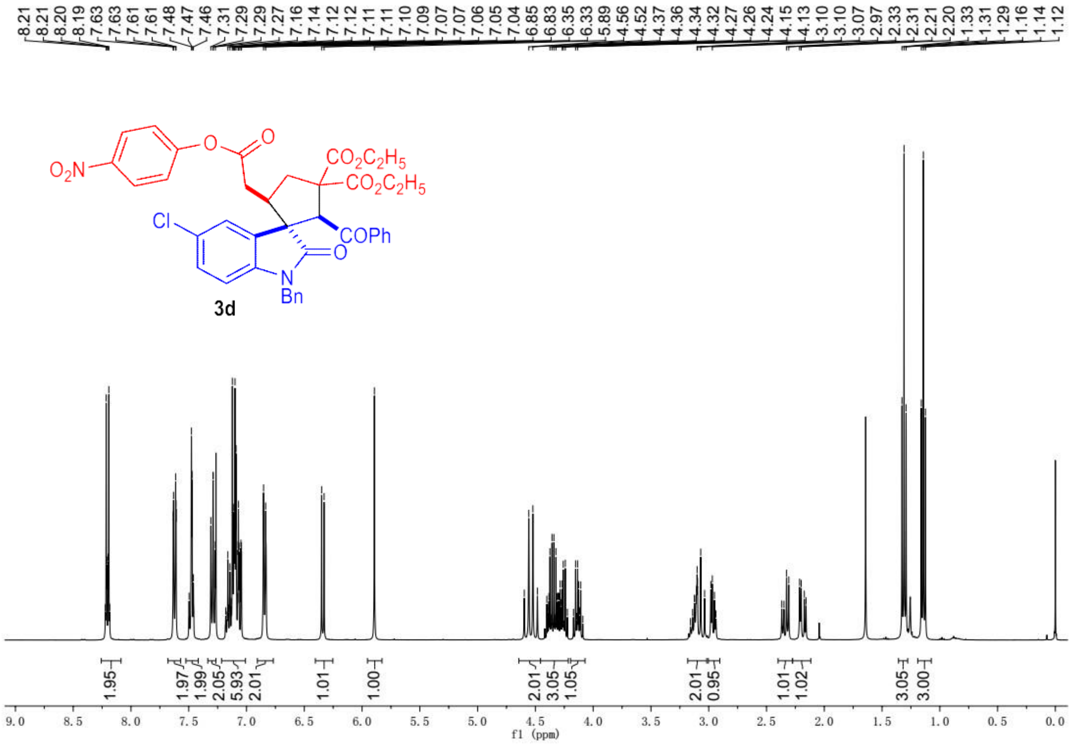

Supplementary Figure S9 ^{1}H NMR spectrum of **3d** (600MHz, $CDCl_3$)

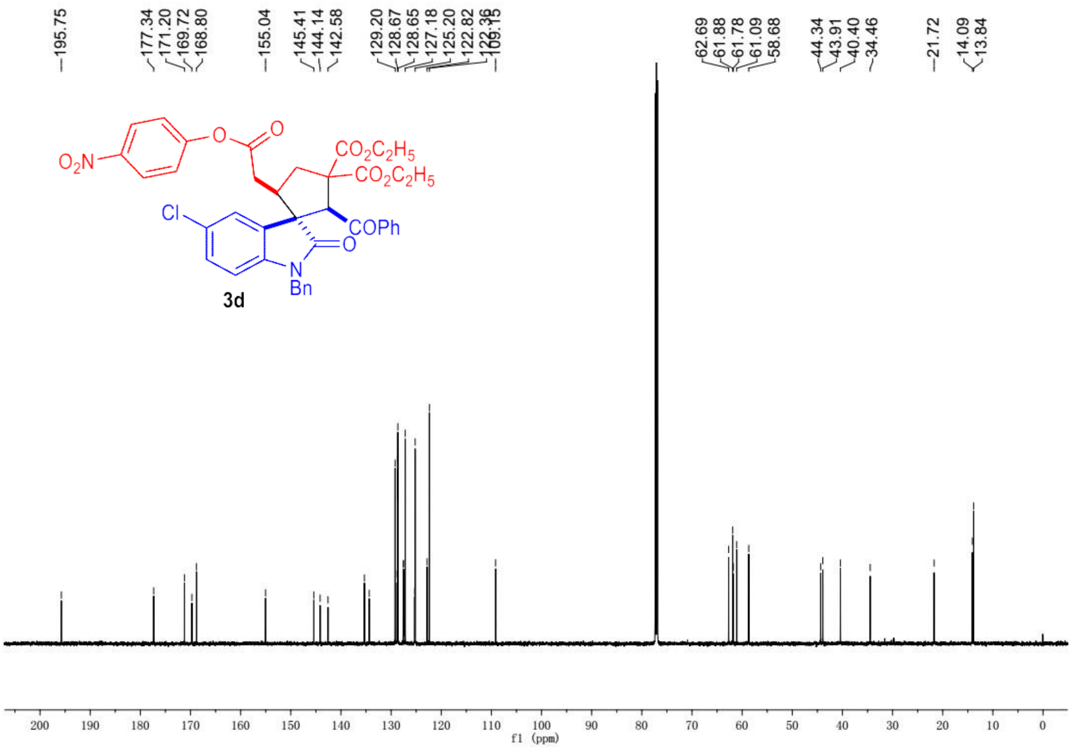

Supplementary Figure S10 ^{13}C NMR spectrum of **3d** (151 MHz, $CDCl_3$)

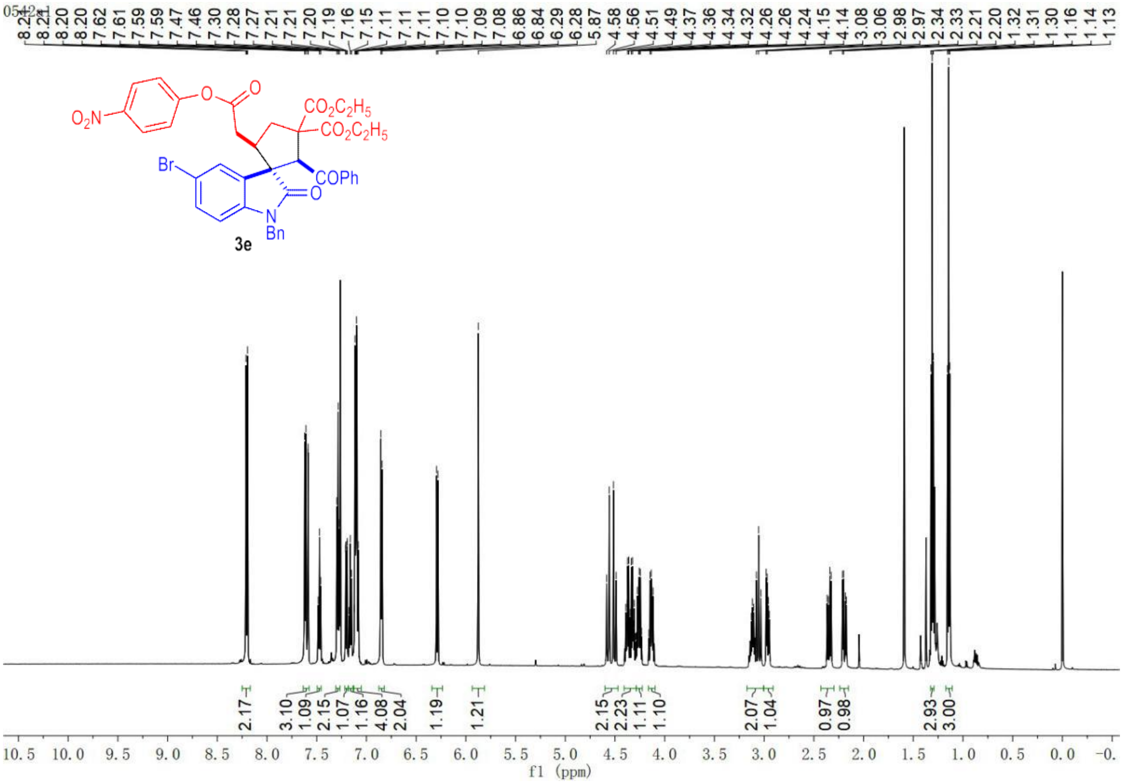

Supplementary Figure S11 ^{1}H NMR spectrumof **3e** (600MHz, $CDCl_3$)

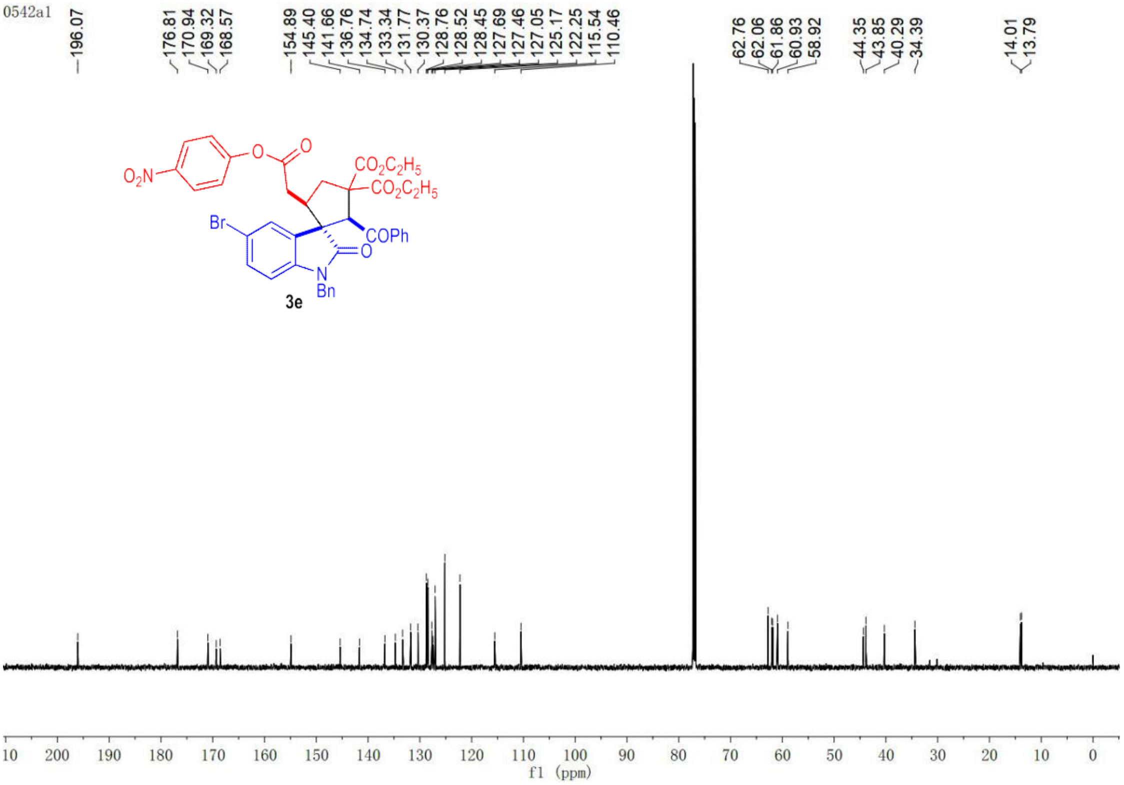

Supplementary Figure S12 ^{13}C NMR spectrum of **3e** (151 MHz, CDCl

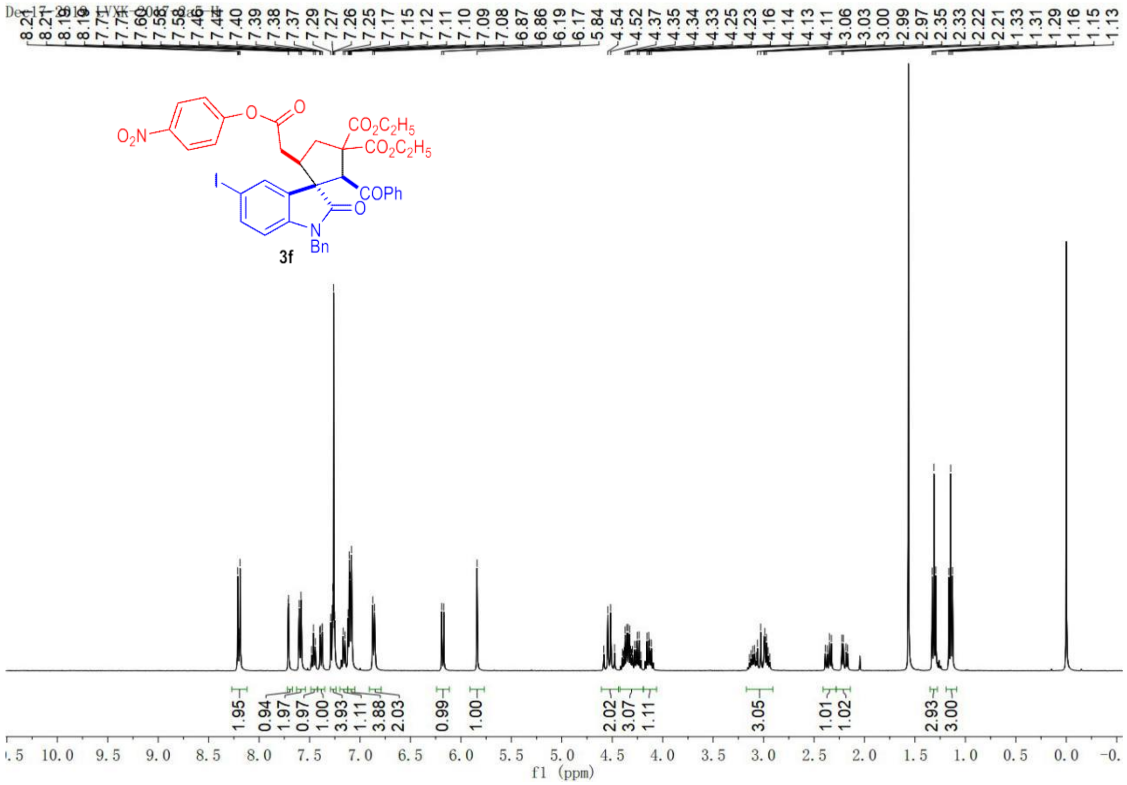

Supplementary Figure S13 ^{1}H NMR spectrumof **3f** (600MHz,$CDCl_3$)

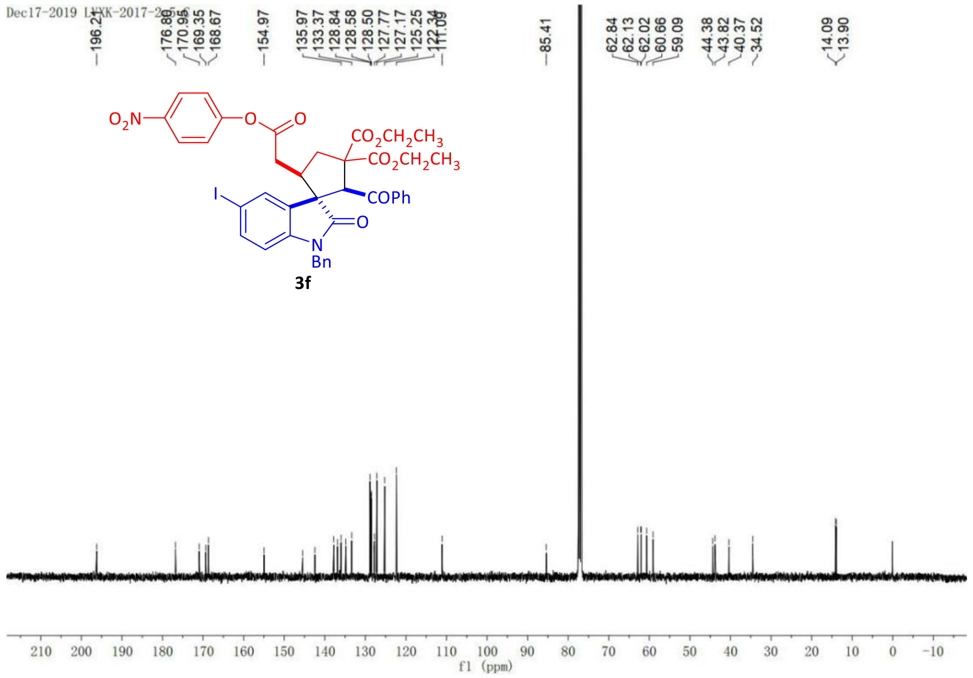

Supplementary Figure S14 ^{13}C NMR spectrum of **3f** (151 MHz, $CDCl_3$

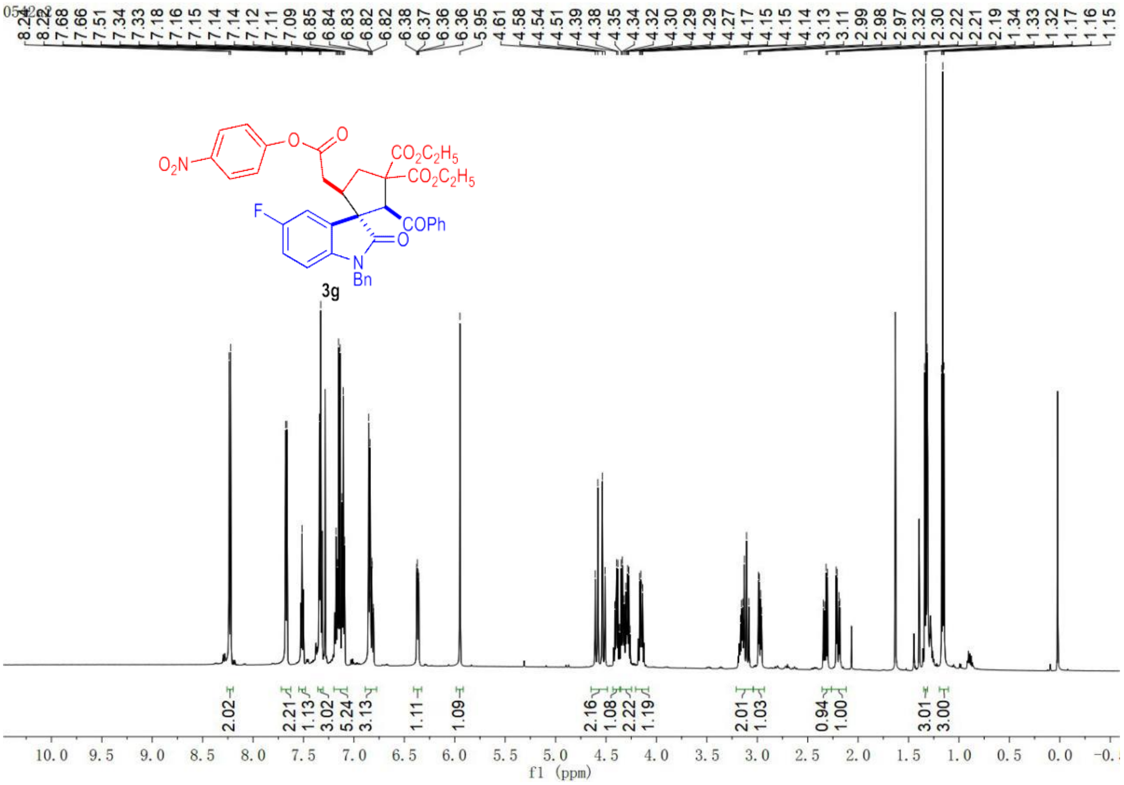

Supplementary Figure S15 ^{1}H NMR spectrum of **3g** (600 MHz, $CDCl_3$)

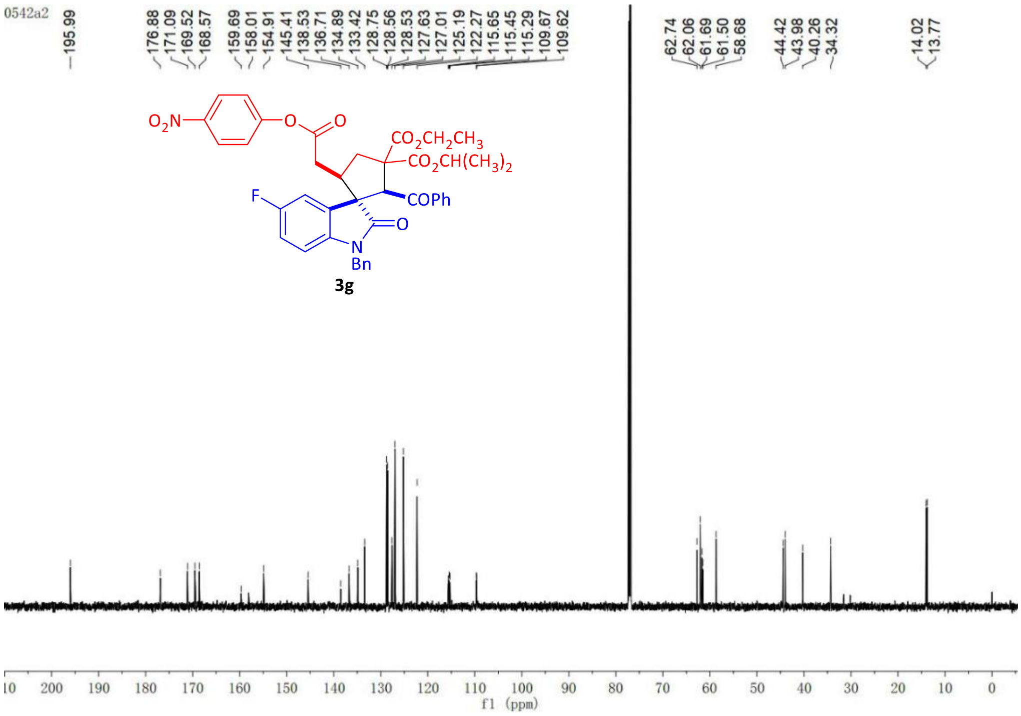

Supplementary Figure S16 ^{13}C NMR spectrum of **3g** (151 MHz, $CDCl_3$)

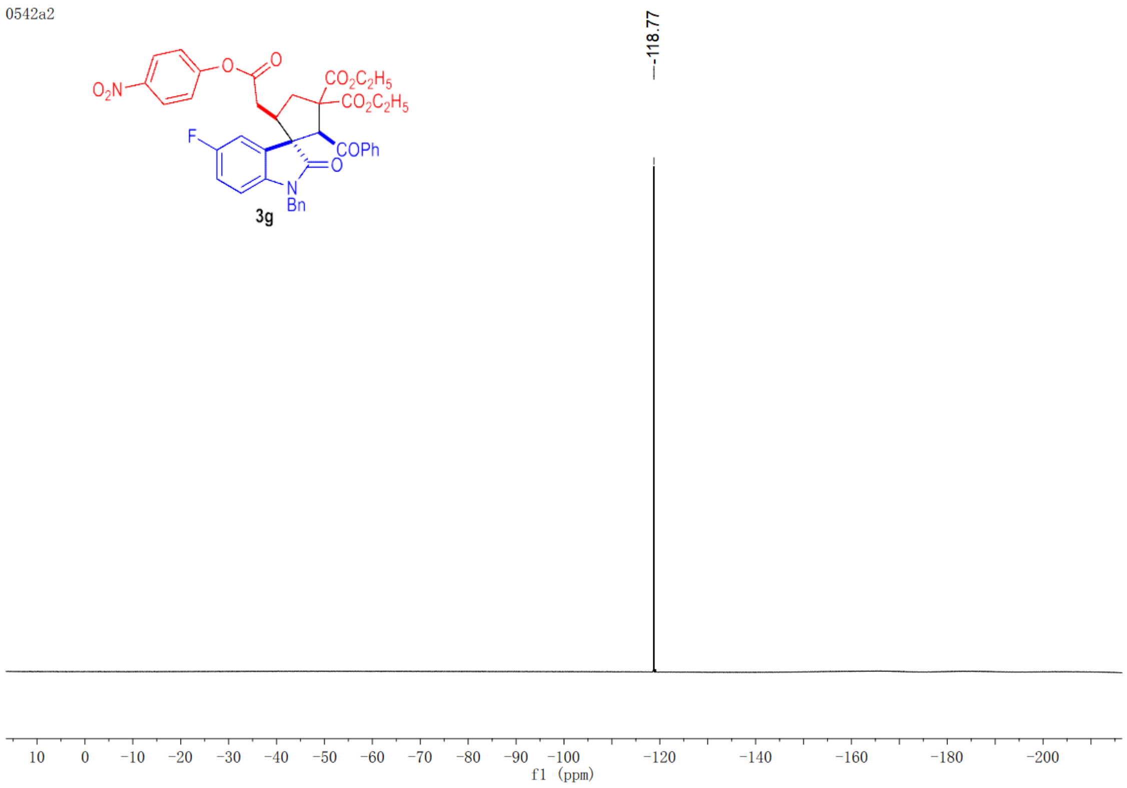

Supplementary Figure S17 ^{19}F NMR spectrum of **3g** (565 MHz, $CDCl_3$)

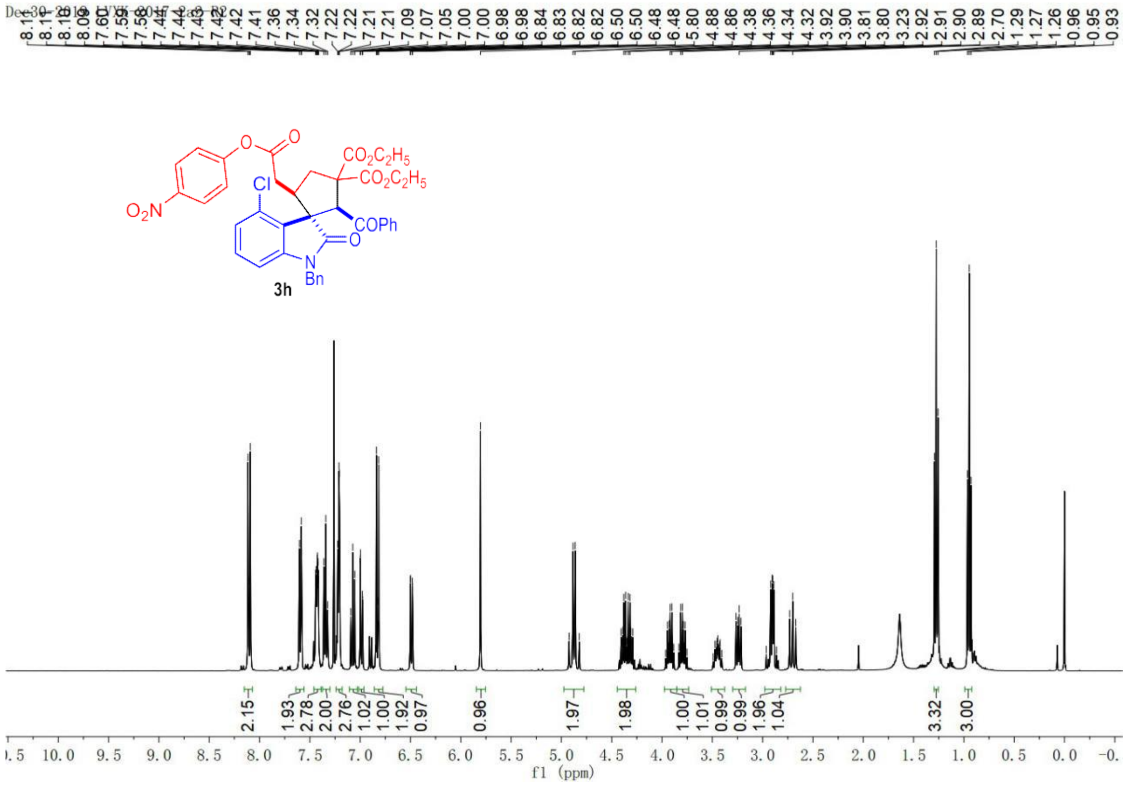

Supplementary Figure S18 ^{1}H NMR spectrum of **3h** (600 MHz, $CDCl_3$)

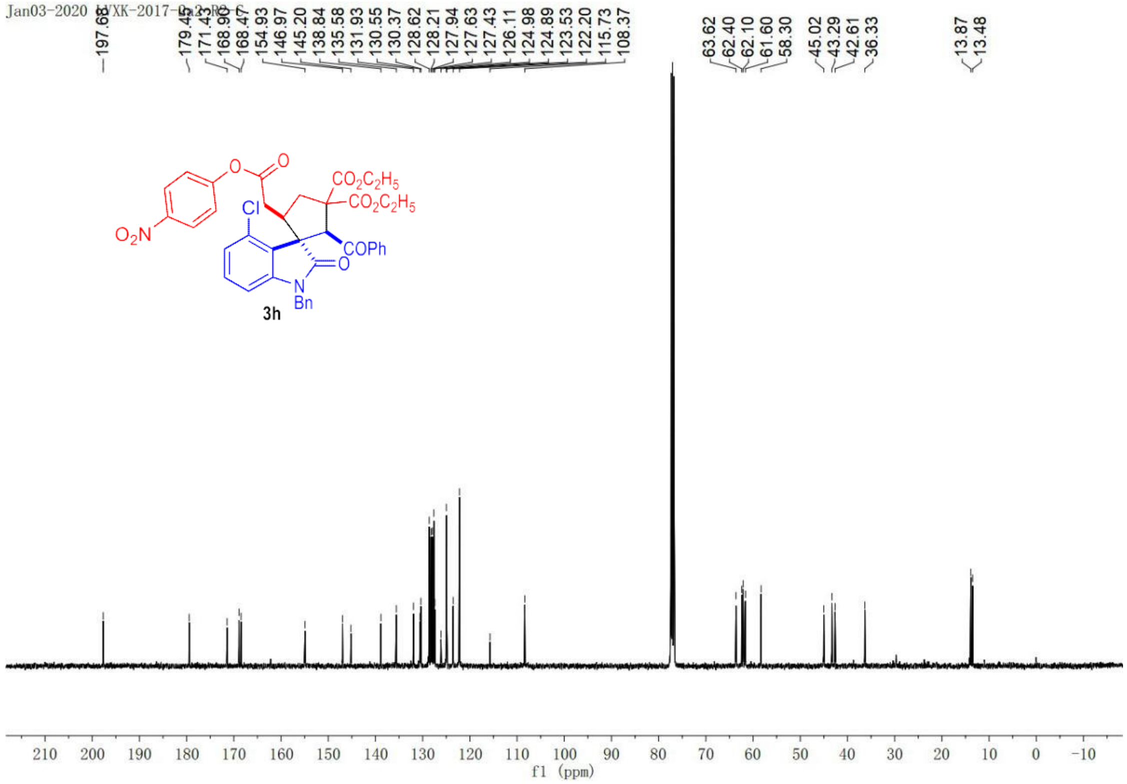

Supplementary Figure S19 ^{13}C NMR spectrum of **3h** (151 MHz, $CDCl_3$)

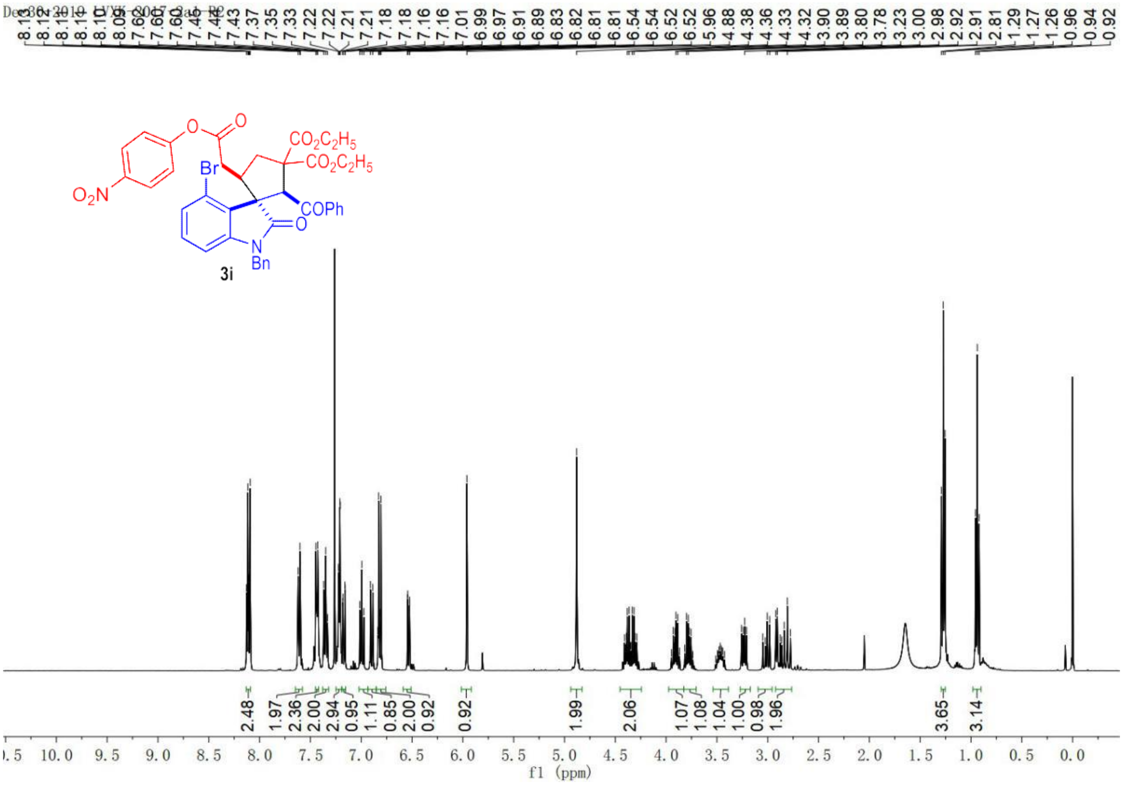

Supplementary Figure S20 ^{1}H NMR spectrum of **3i** (600MHz, $CDCl_3$)

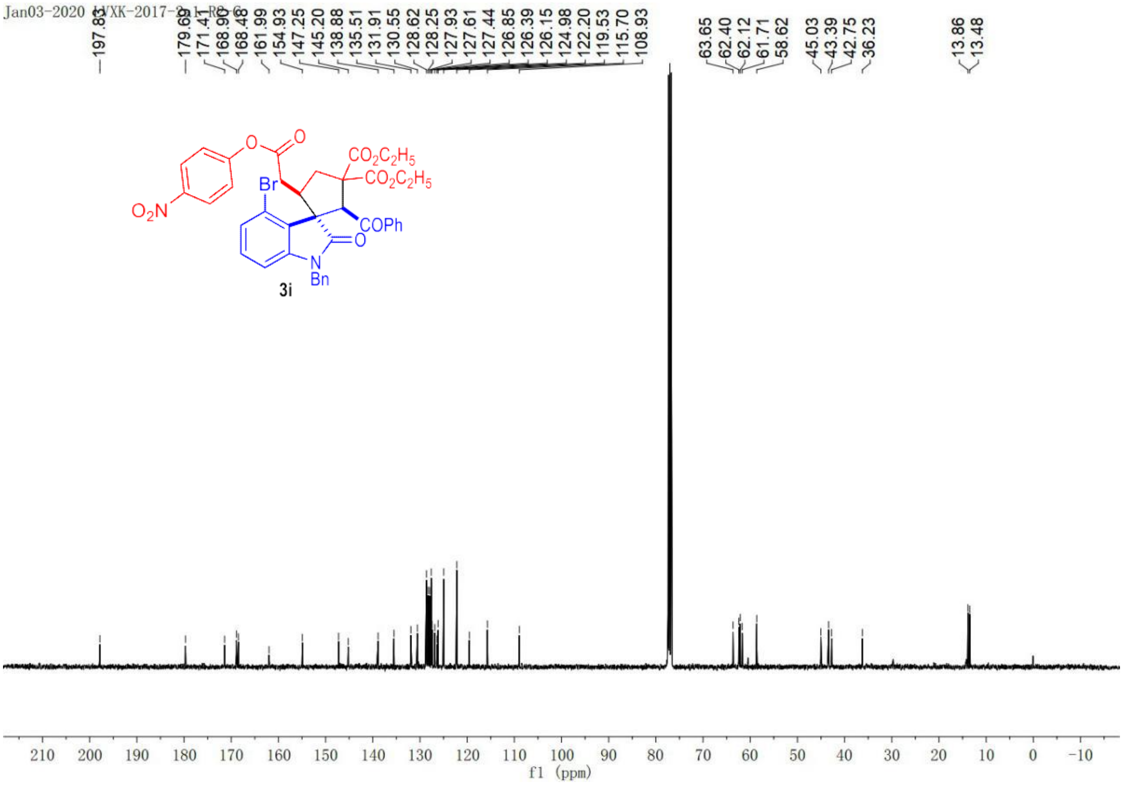

Supplementary Figure S21 ^{13}C NMR spectrum of **3i** (151 MHz, $CDCl_3$)

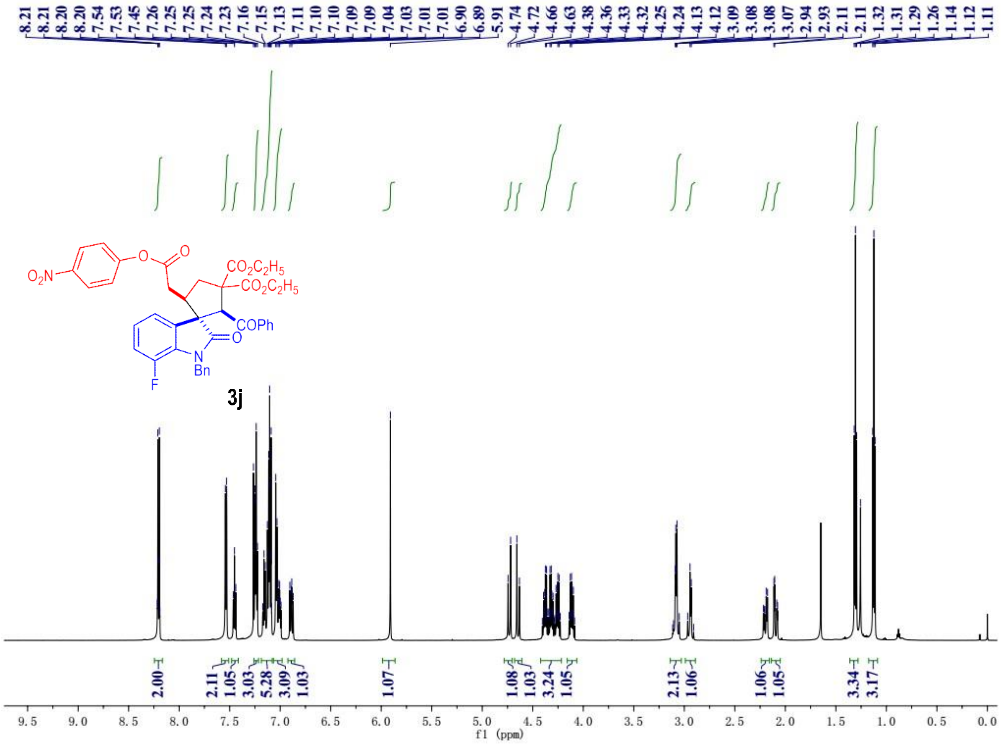

Supplementary Figure S22 ^{1}H NMR spectrumof **3j** (600MHz, $CDCl_3$)

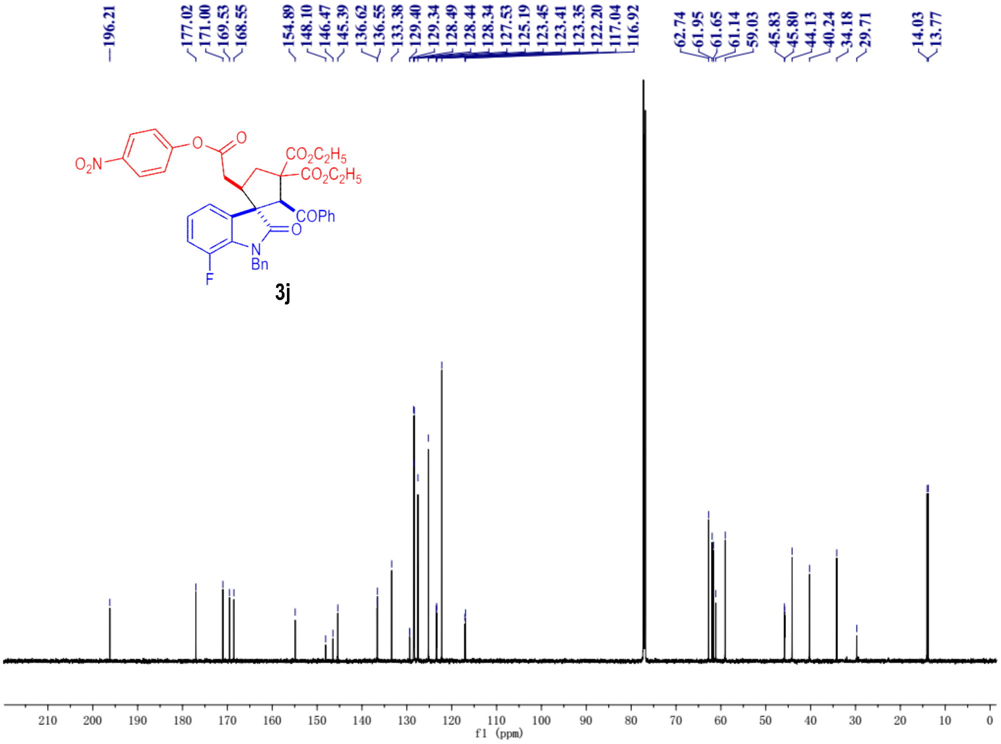

Supplementary Figure S23 ^{13}C NMR spectrum of **3j** (151 MHz, $CDCl_3$)

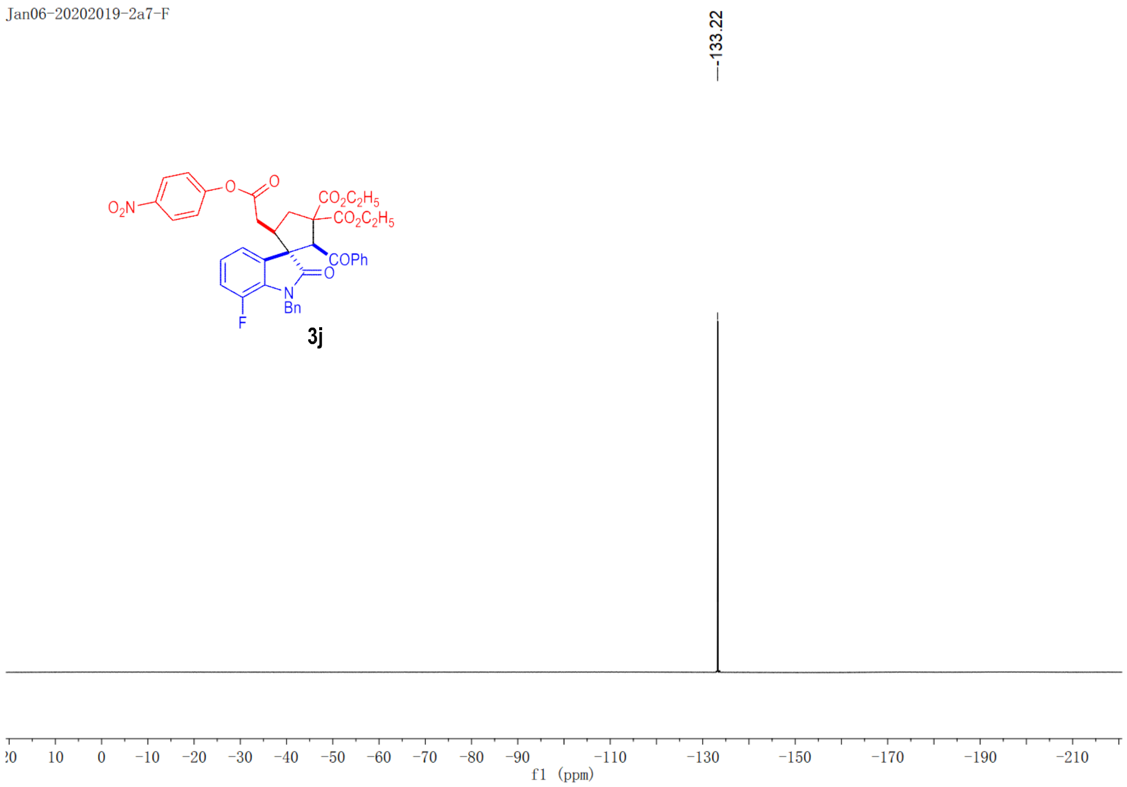

Supplementary Figure S24 ^{19}F NMR spectrum of **3j** (377 MHz, $CDCl_3$)

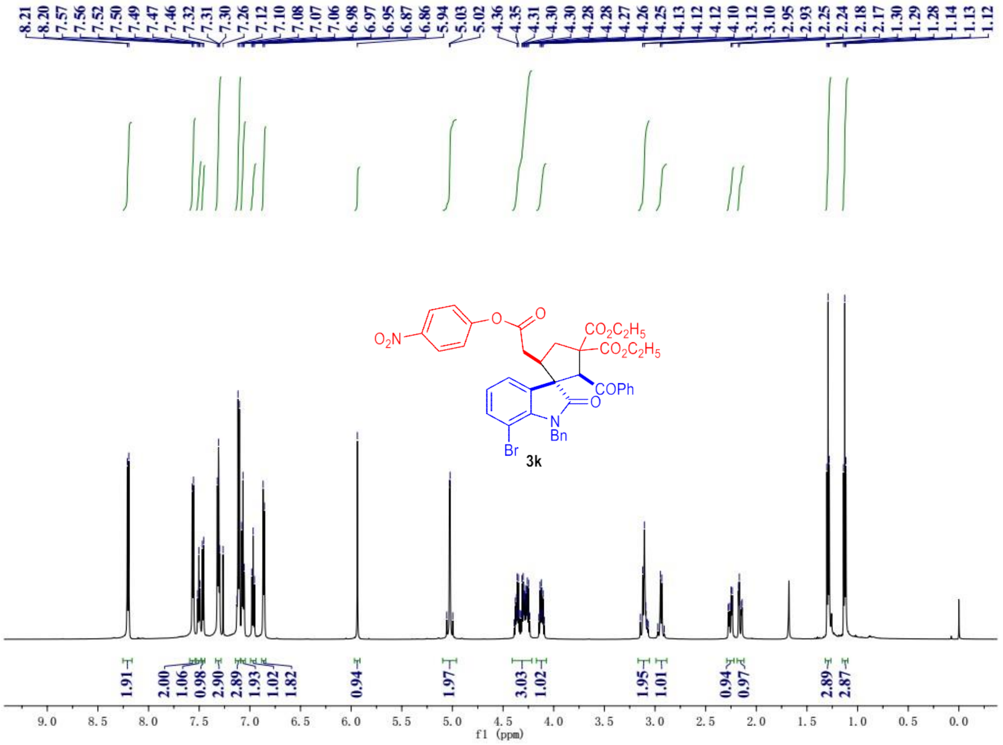

Supplementary Figure S25 ^{1}H NMR spectrum of **3k** (600 MHz, $CDCl_3$)

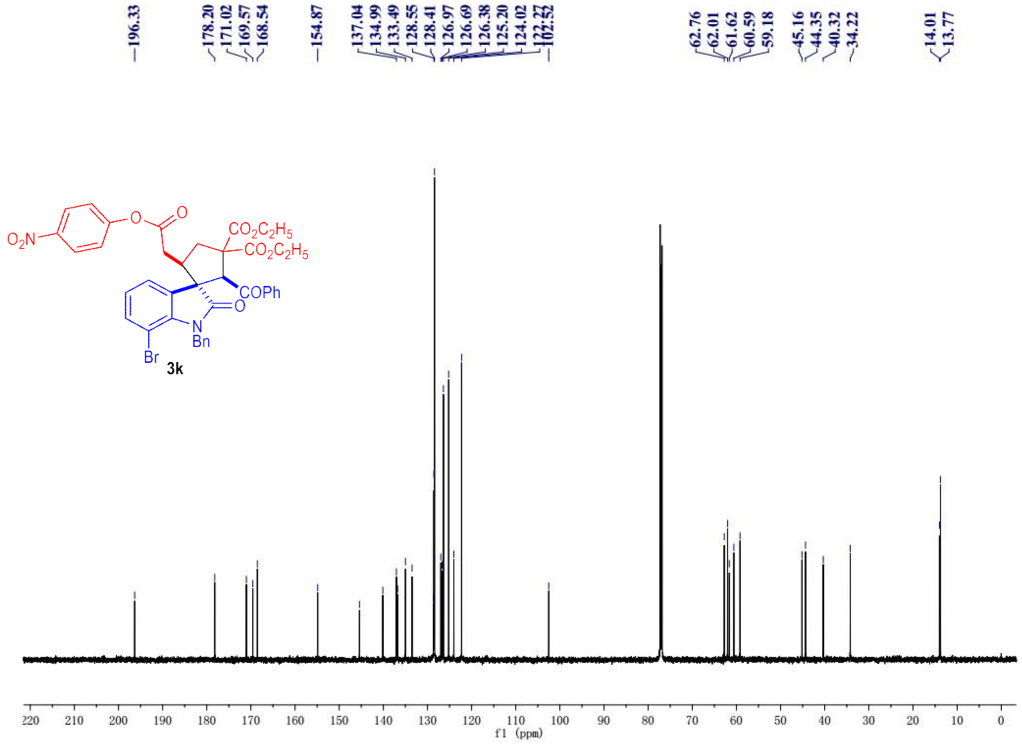

Supplementary Figure S26 ^{13}C NMR spectrum of **3k** (151 MHz, $CDCl_3$)

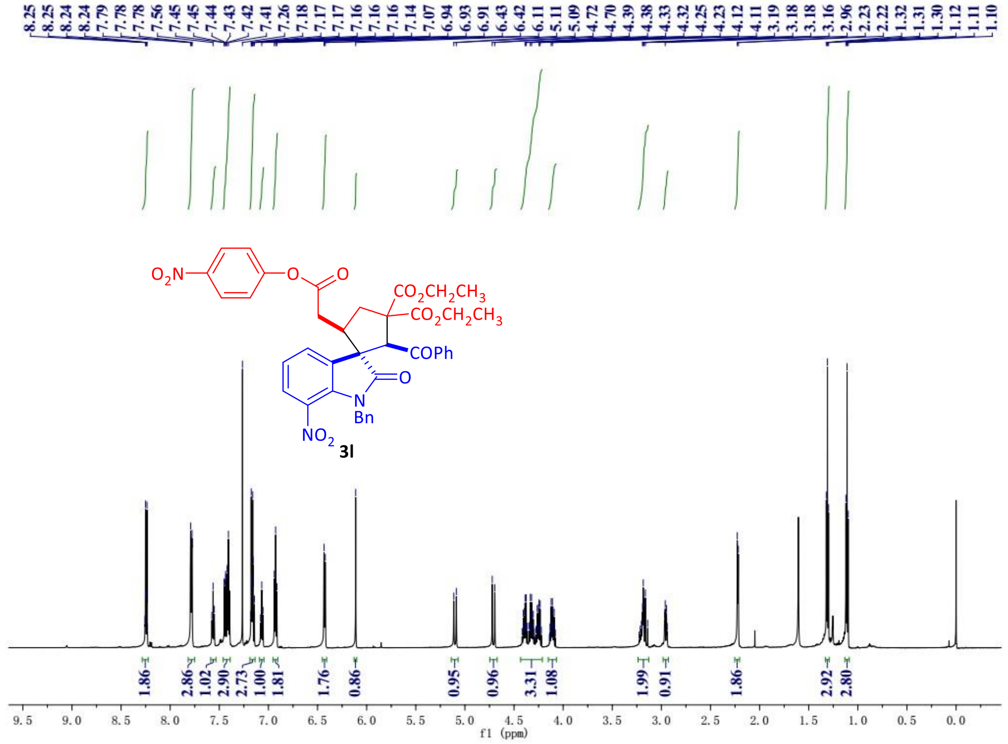

Supplementary Figure S27 ^{1}H NMR spectrum of **3l** (600MHz, $CDCl_3$)

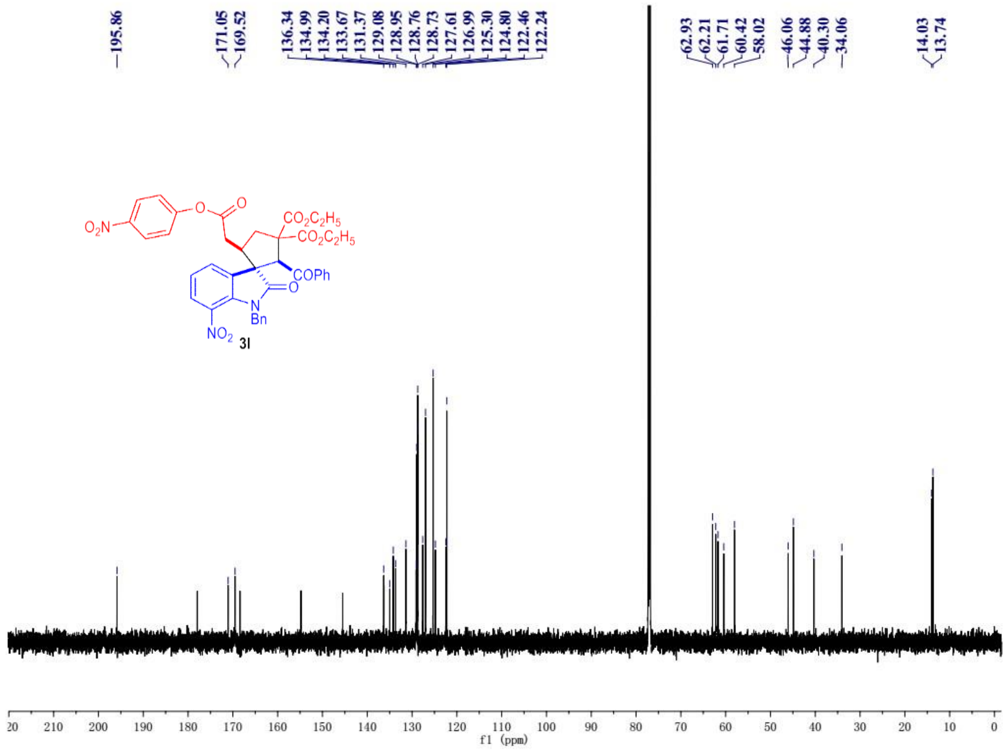

Supplementary Figure S28 ^{13}C NMR spectrum of **3l** (151 MHz, $CDCl_3$)

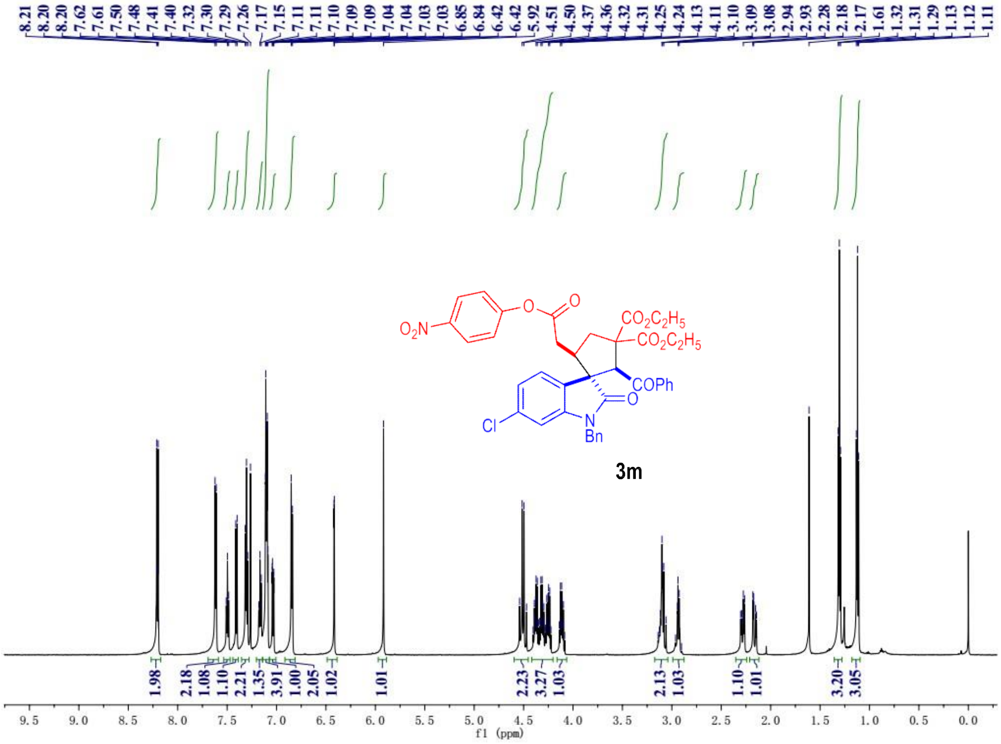

Supplementary Figure S29 ^{1}H NMR spectrum of **3m** (600 MHz, CDCl^{3})

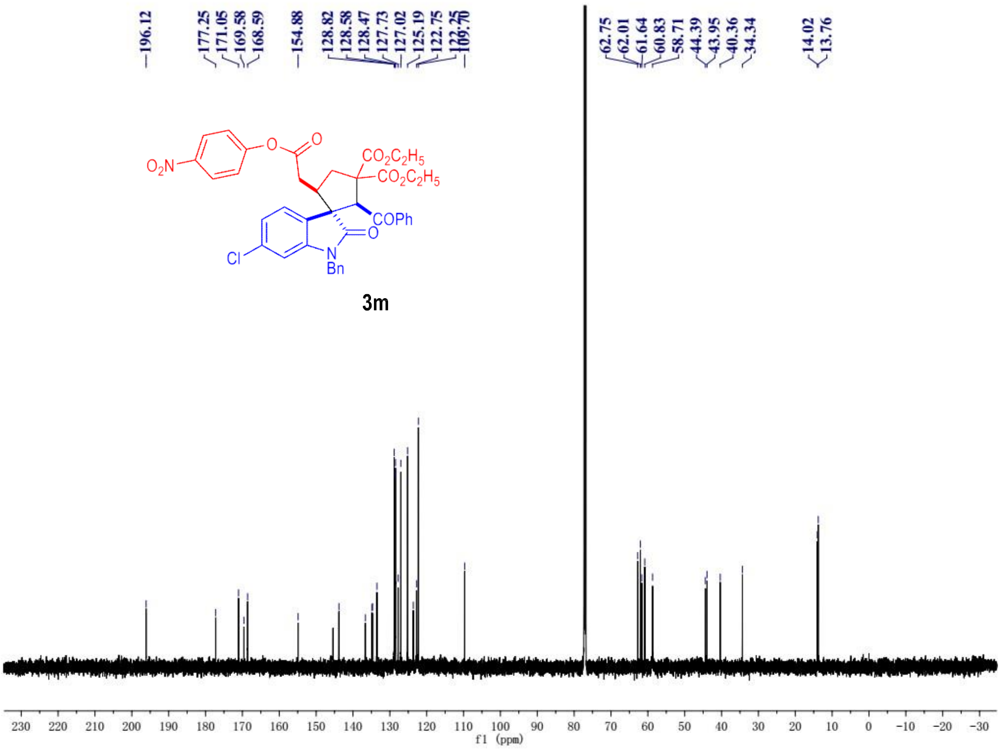

Supplementary Figure S30 ^{13}C NMR spectrum of **3m** (151 MHz, $CDCl_3$)

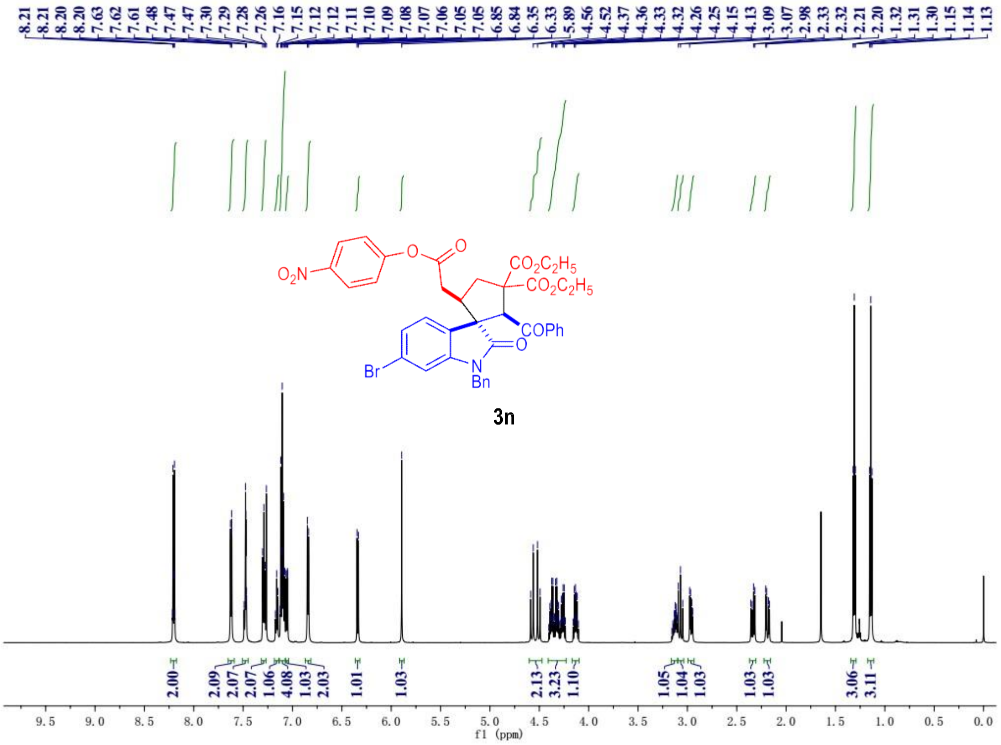

Supplementary Figure S31 ^{1}H NMR spectrum of **3n** (600 MHz, $CDCl_3$)

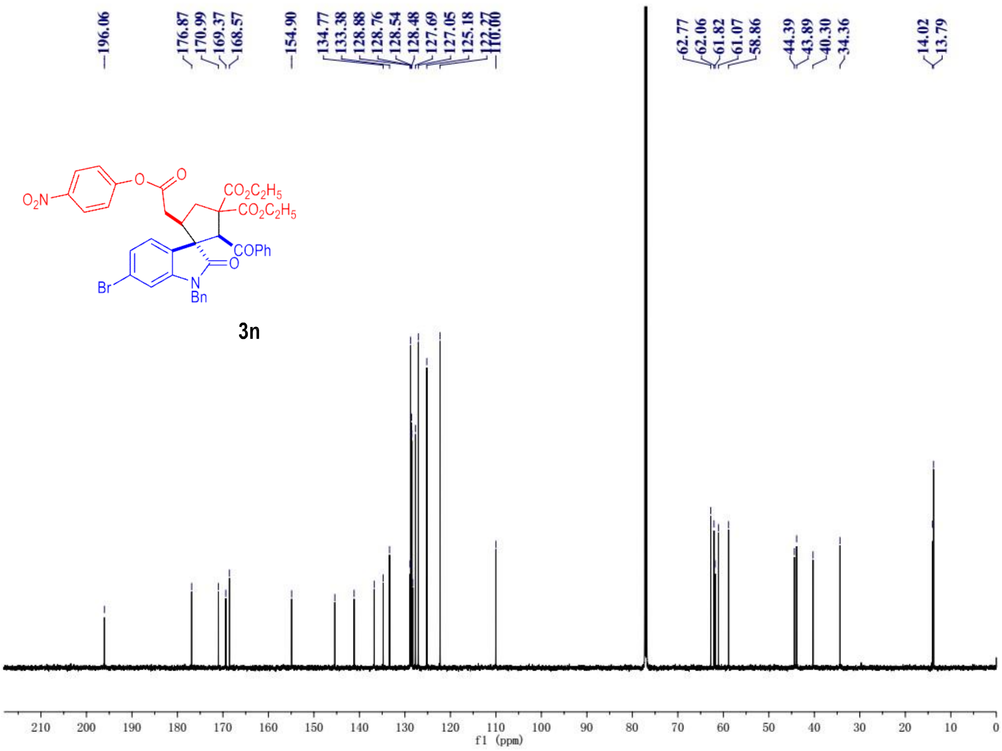

Supplementary Figure S32 ^{13}C NMR spectrum of **3n** (151 MHz, $CDCl_3$)

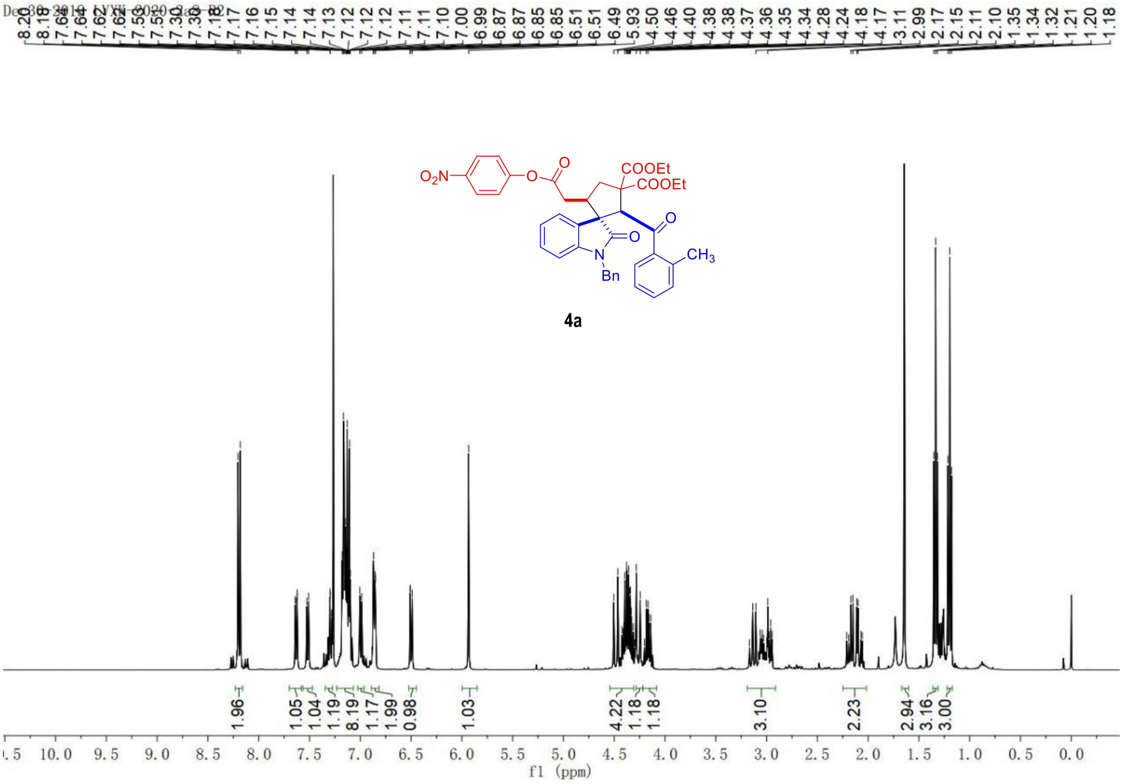

Supplementary Figure S33 ^{1}H NMR spectrum of **4a** (600 MHz, $CDCl_3$)

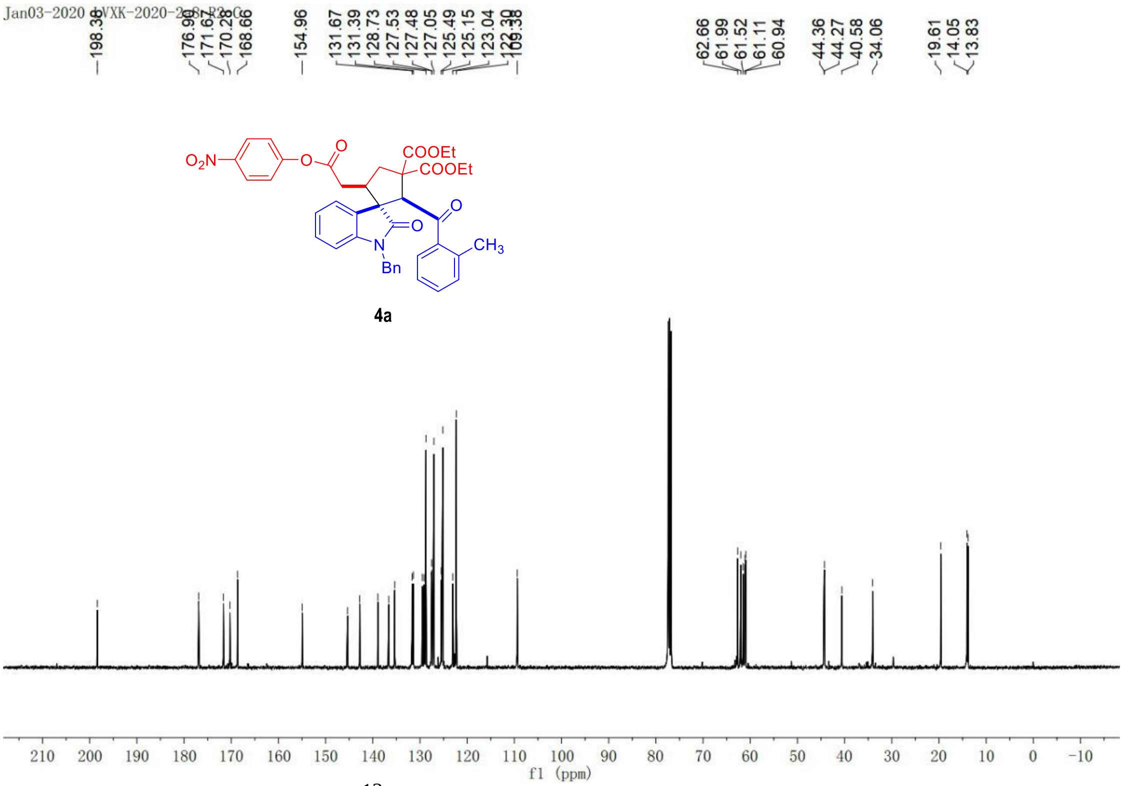

Supplementary Figure S34 ^{13}C NMR spectrum of **4a** (151 MHz, $CDCl_3$)

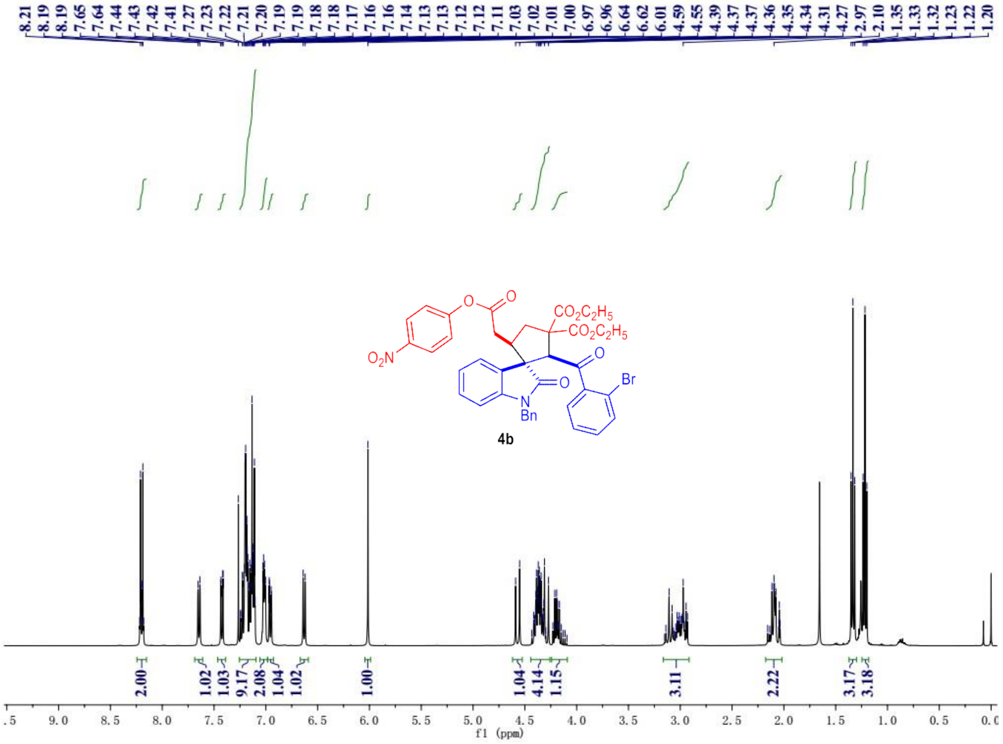

Supplementary Figure S35 ^{1}H NMR spectrum of **4b** (600MHz, $CDCl_3$)

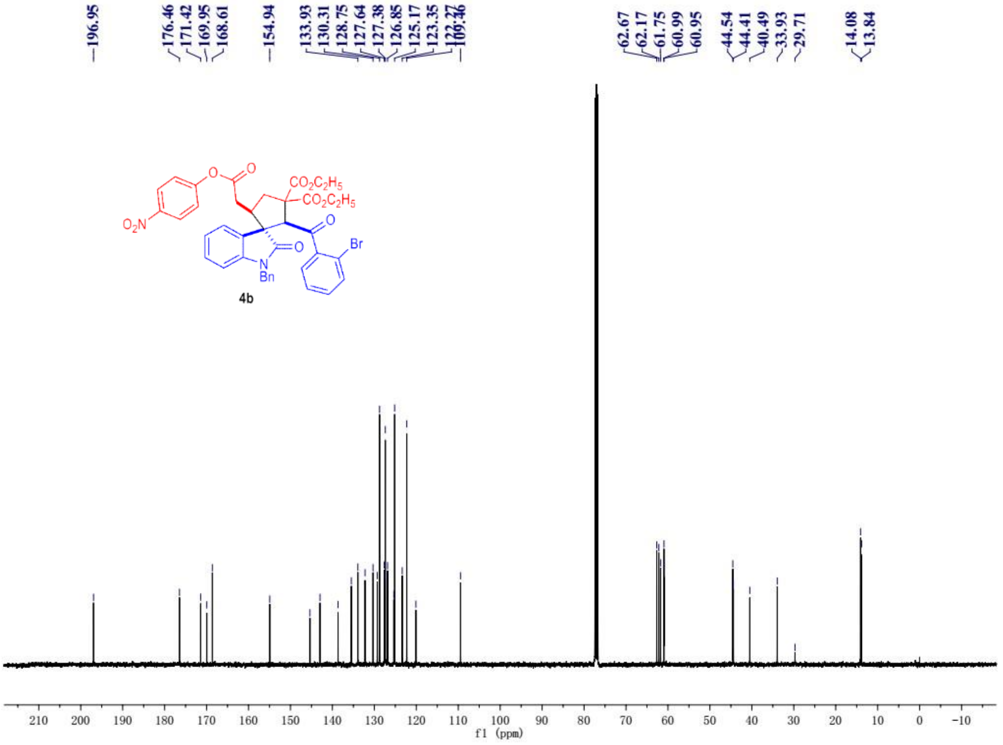

Supplementary Figure S36 ^{13}C NMR spectrum of **4b** (151 MHz, $CDCl_3$)

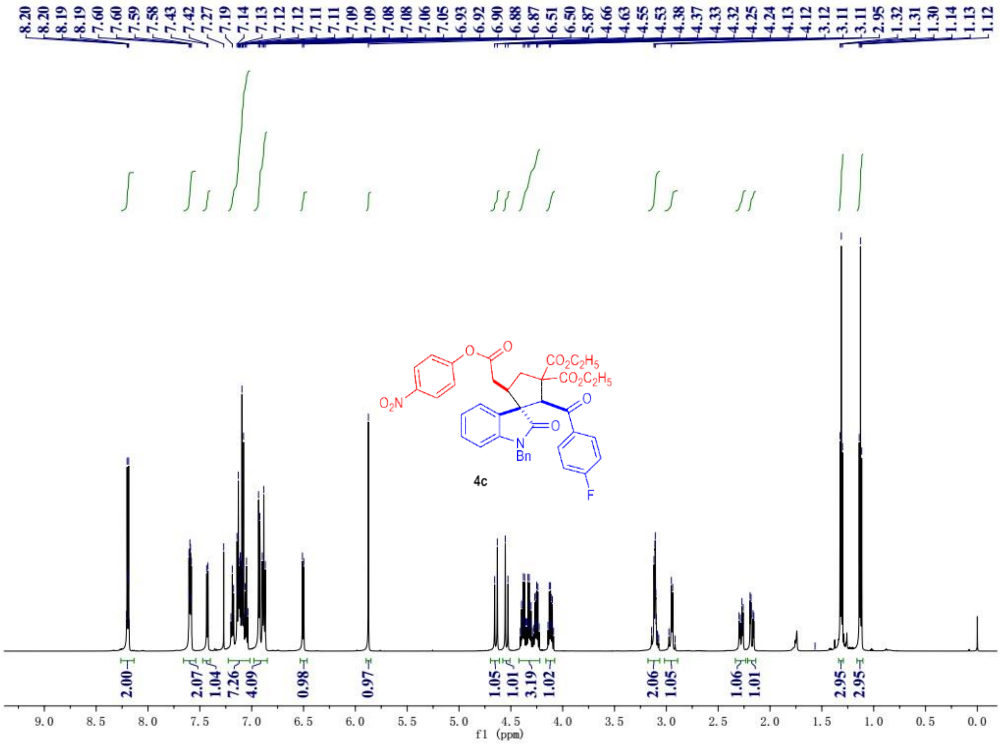

Supplementary Figure S37 ^{1}H NMR spectrumof **4c** (600MHz, $CDCl_3$)

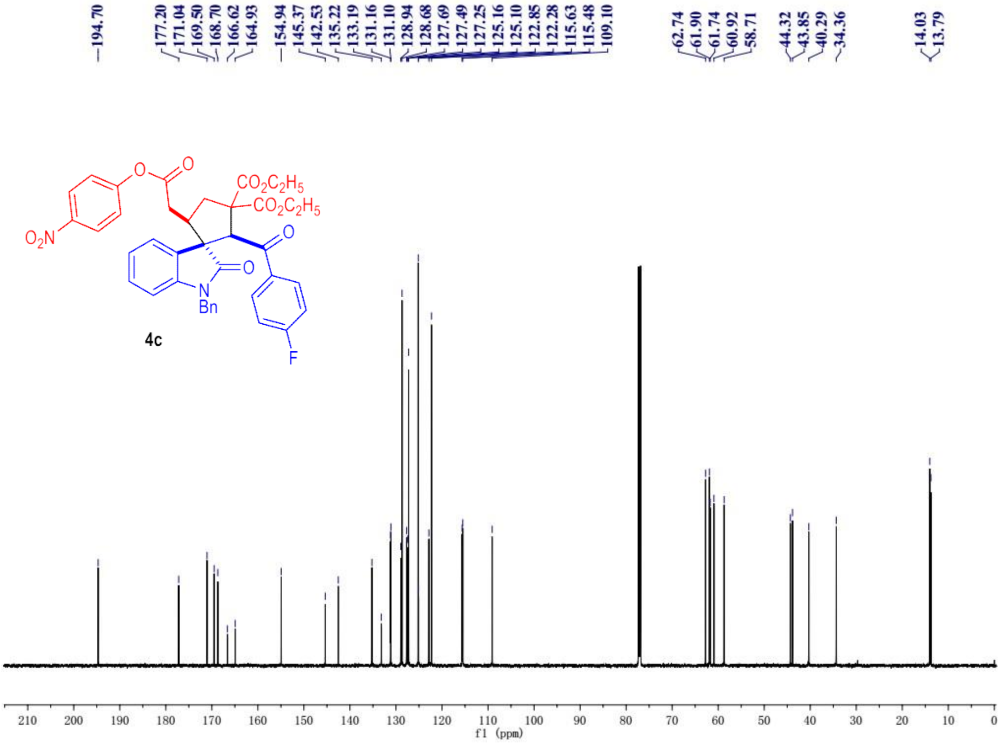

Supplementary Figure S38 ^{13}C NMR spectrum of **4c** (151 MHz, $CDCl_3$)

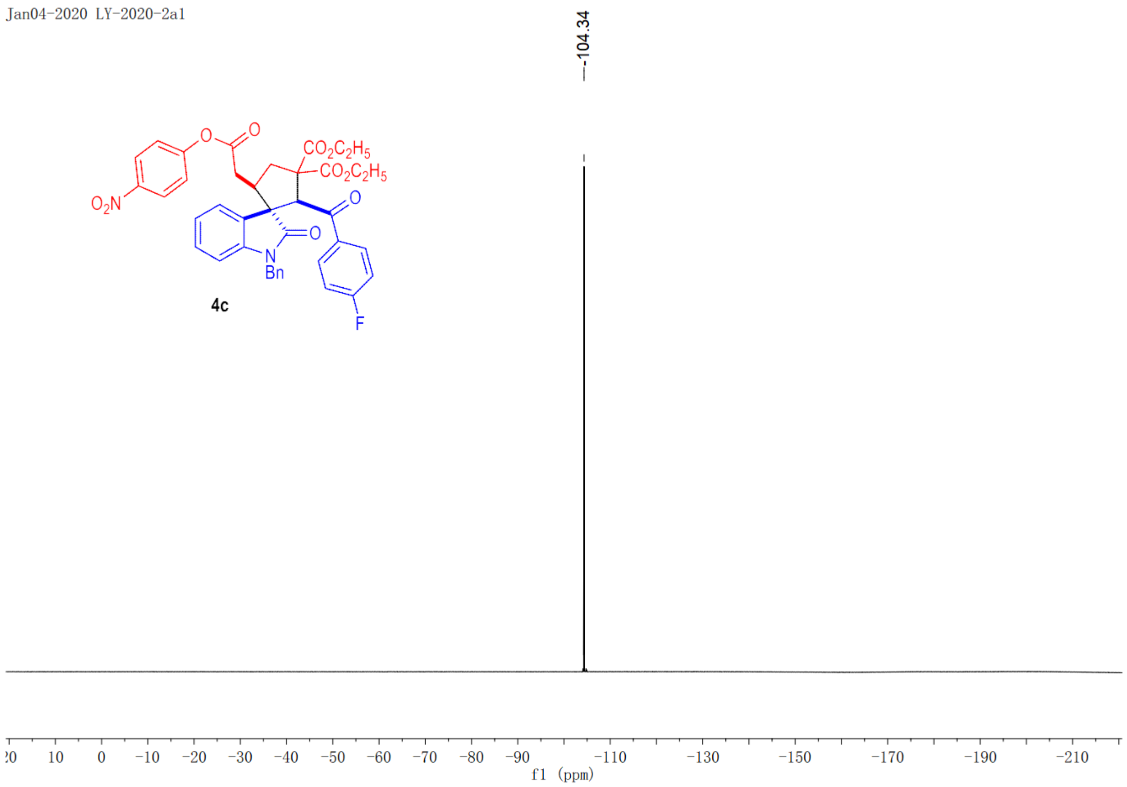

Supplementary Figure S39 ^{19}F NMR spectrum of **4c** (565 MHz, $CDCl_3$)

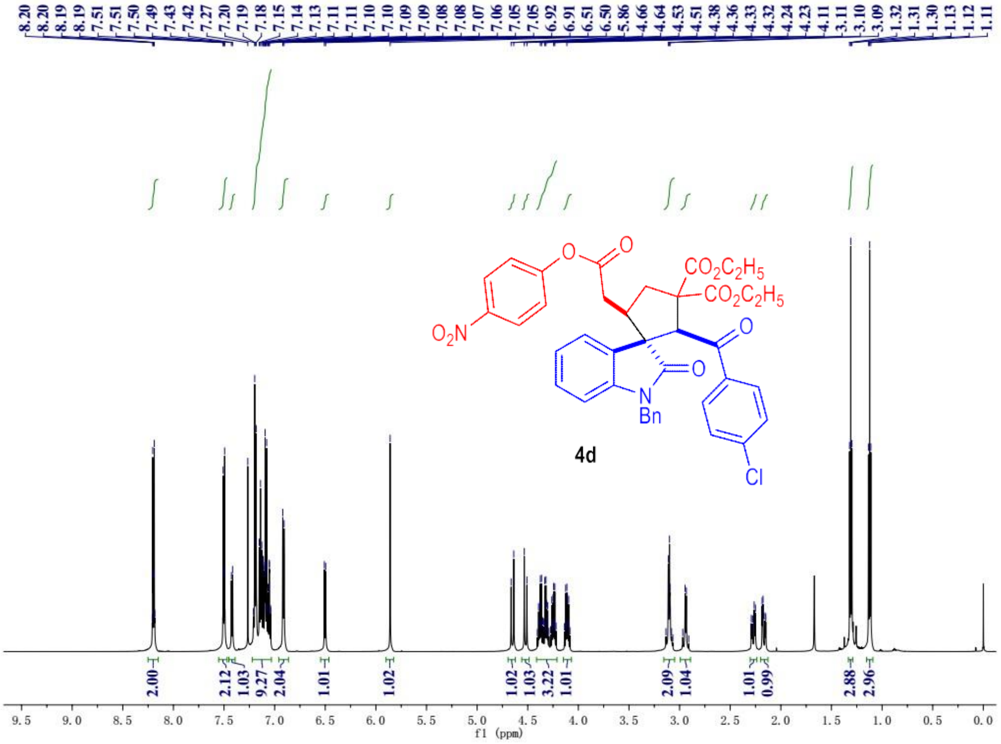

Supplementary Figure S40 ^{1}H NMR spectrum of **4d** (600 MHz, $CDCl_3$)

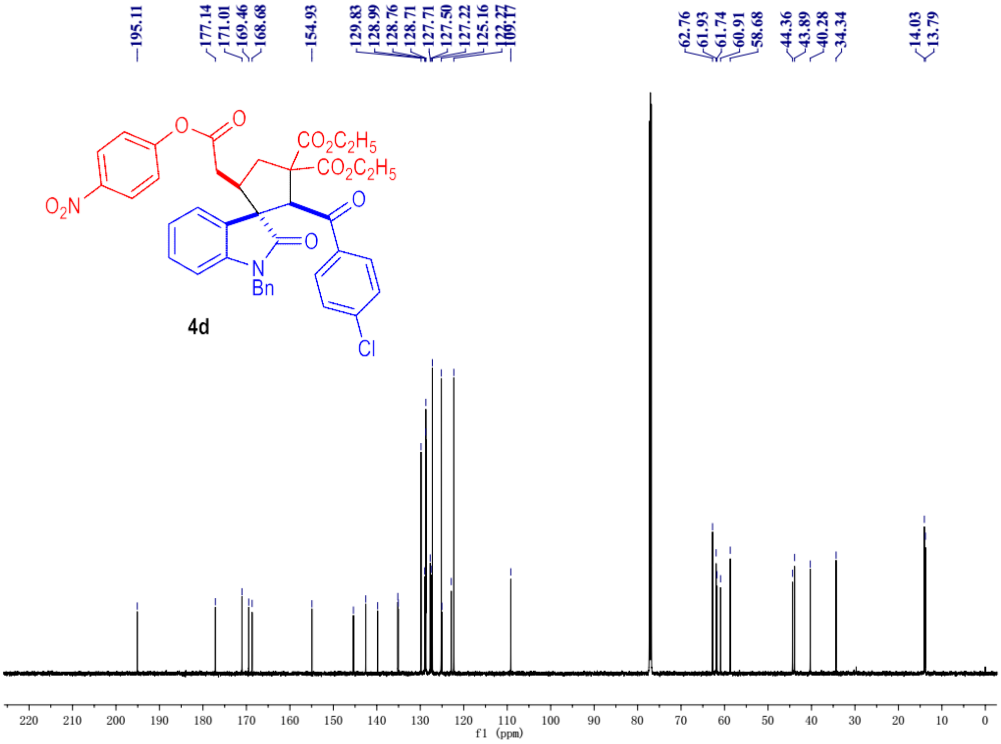

Supplementary Figure S41 ^{13}C NMR spectrum of **4d** (151 MHz, $CDCl_3$)

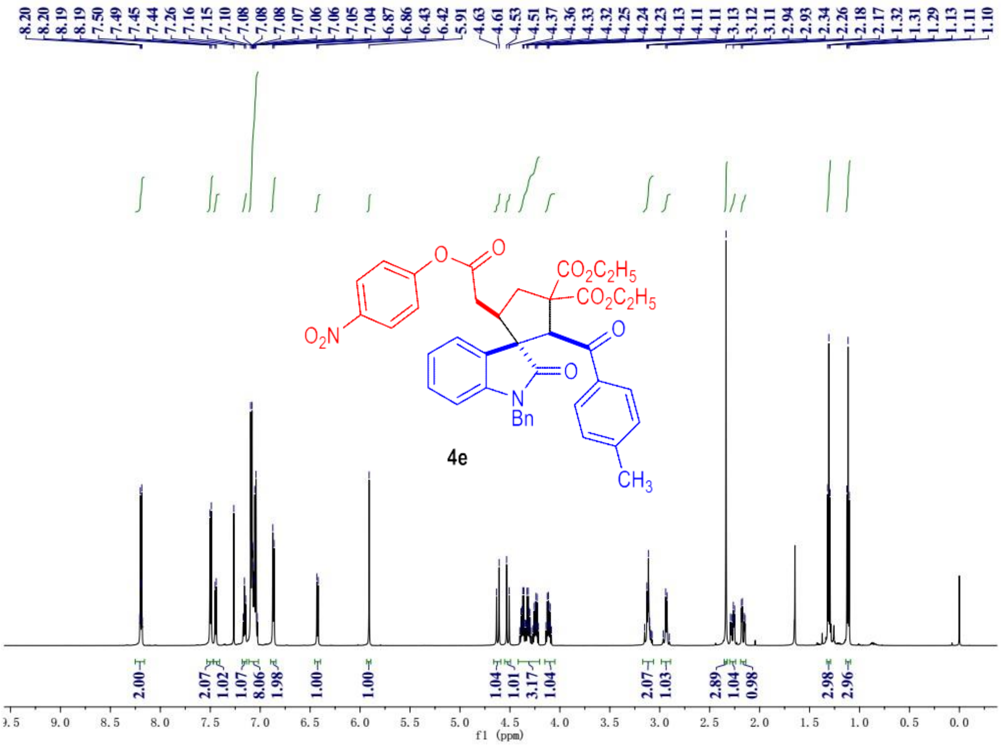

Supplementary Figure S42 ^{1}H NMR spectrum of **4e** (600 MHz, $CDCl_3$)

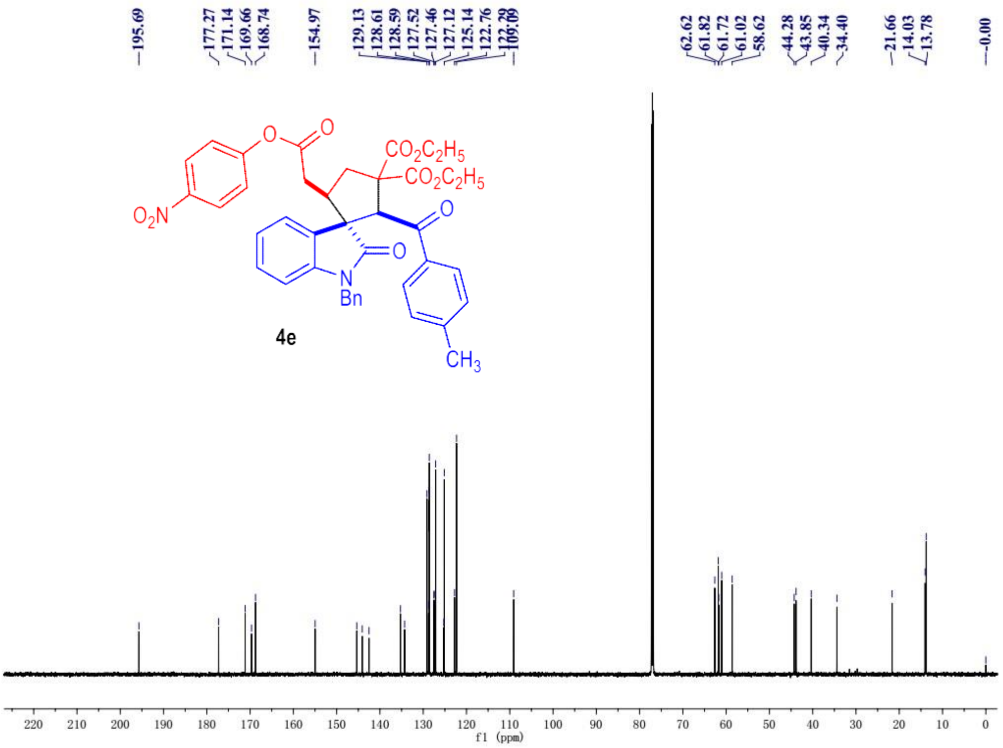

Supplementary Figure S43 ^{13}C NMR spectrum of **4e** (151 MHz, $CDCl_3$)

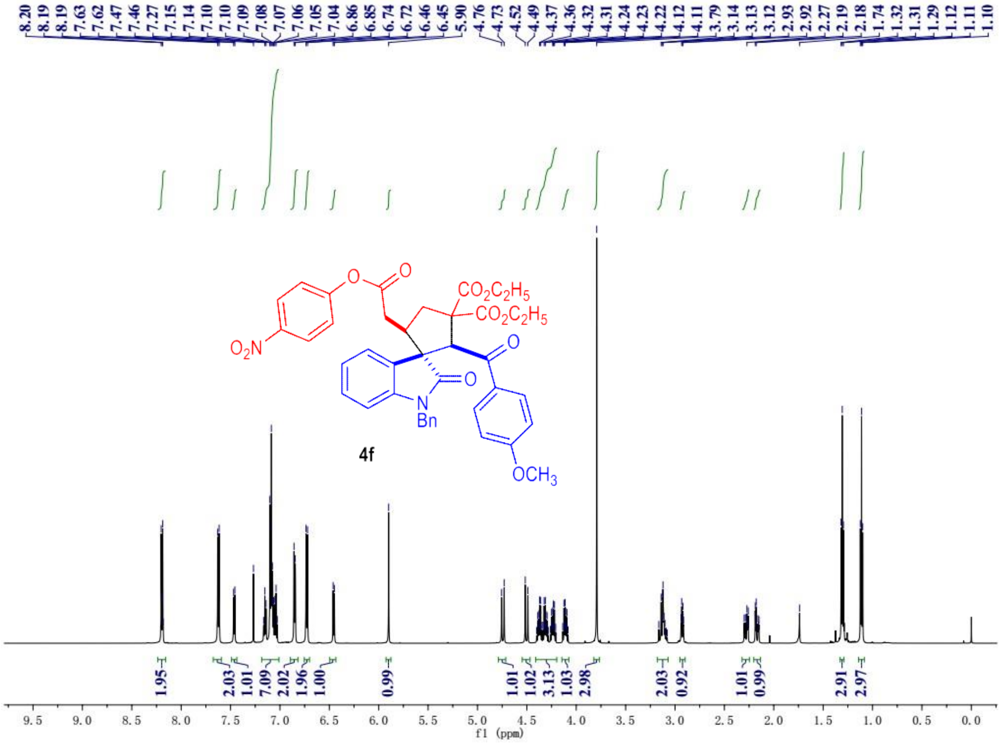

Supplementary Figure S44 ^{1}H NMRspectrumof **4f** (600MHz,CDCl$_3$)

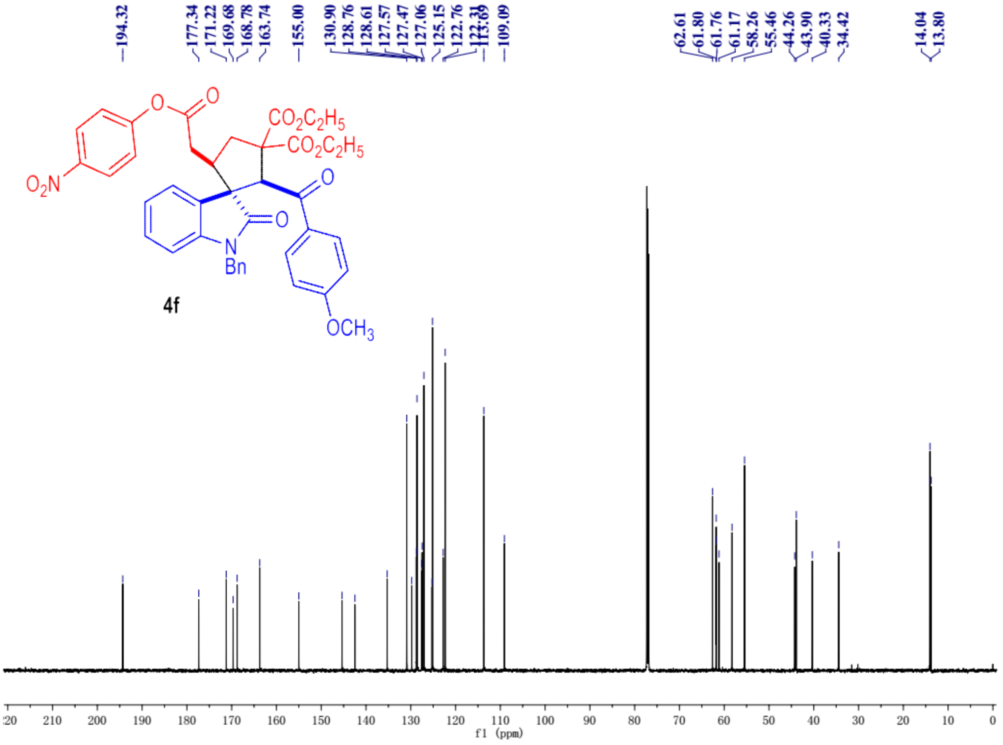

Supplementary Figure S45 ^{13}C NMR spectrum of **4f** (151 MHz, CDCl$_3$)

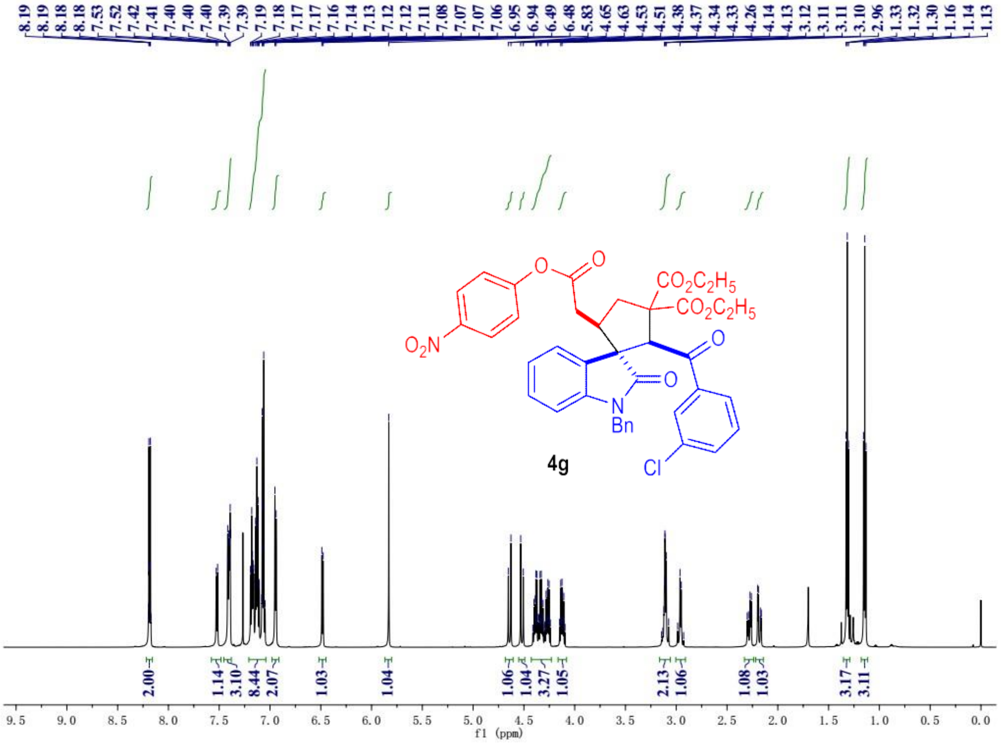

Supplementary Figure S46 ^{1}H NMR spectrum of **4g** (600 MHz, $CDCl_3$)

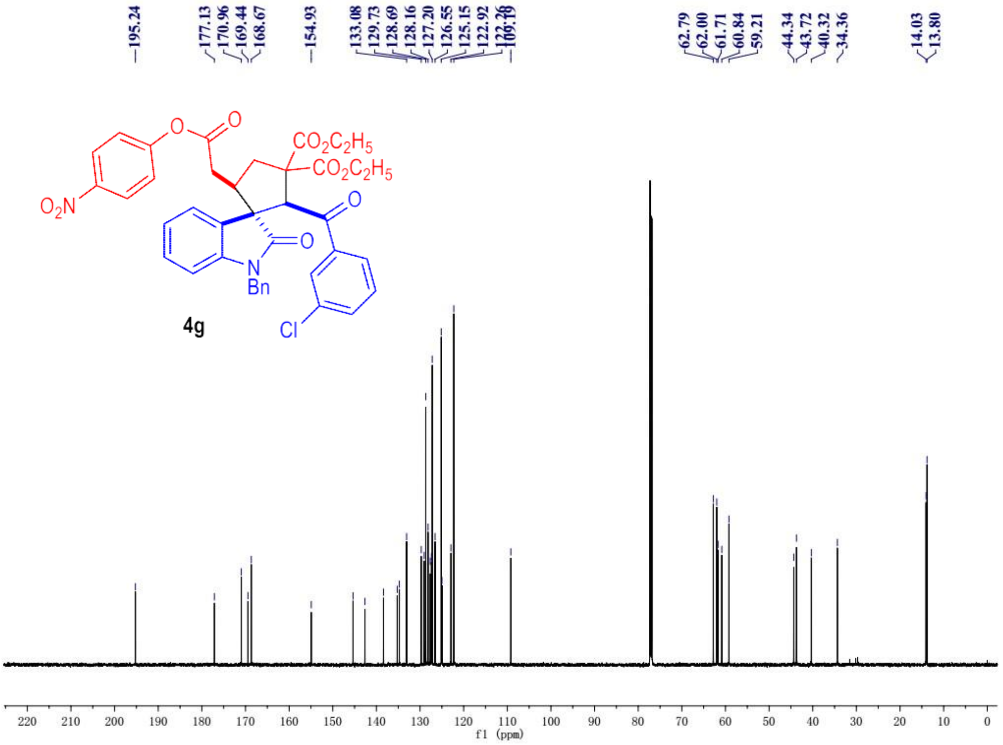

Supplementary Figure S47 ^{13}C NMR spectrum of **4g** (151 MHz, $CDCl_3$)

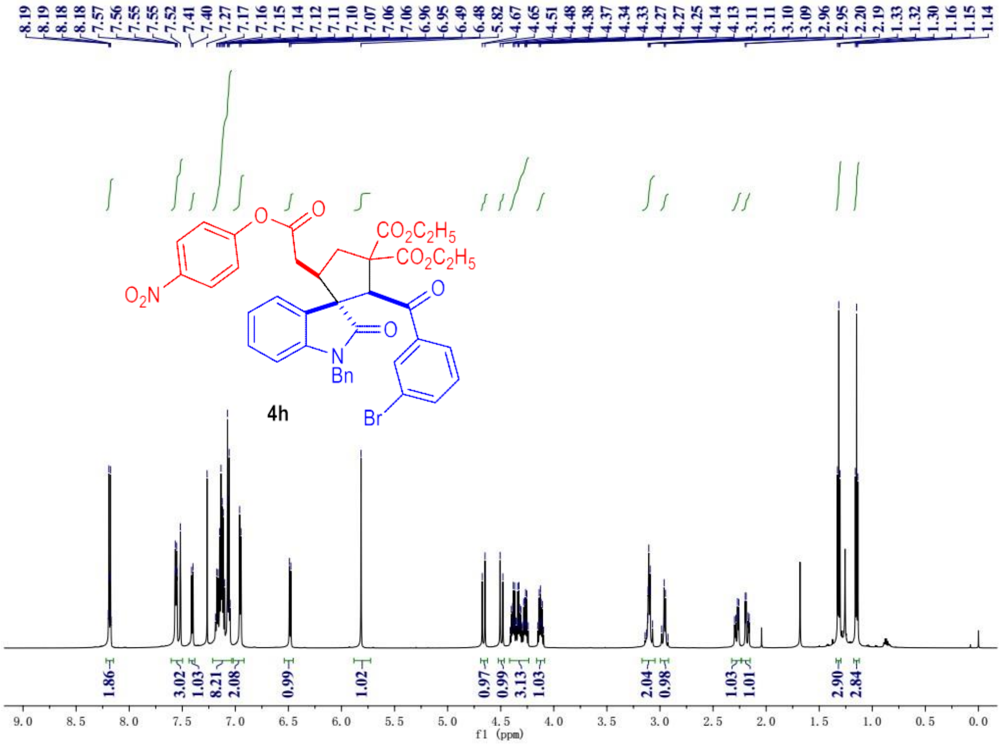

Supplementary Figure S48 ^{1}H MRspectrum of **4h** (600MHz, $CDCl_3$)

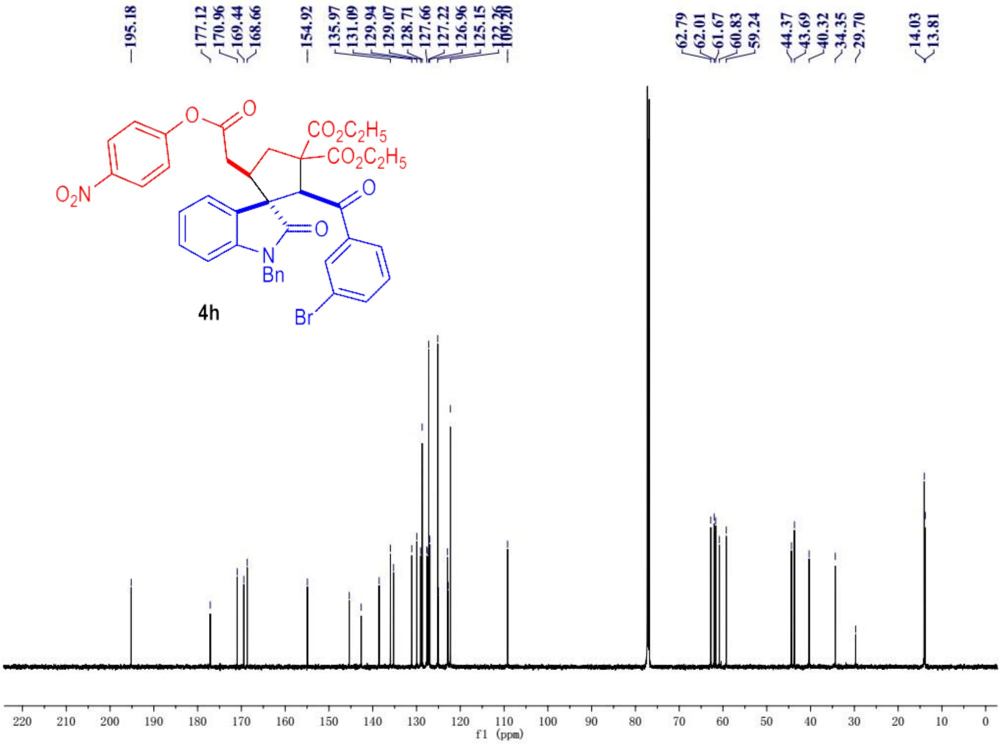

Supplementary Figure S49 ^{13}C NMR spectrum of **4h** (151 MHz, $CDCl_3$)

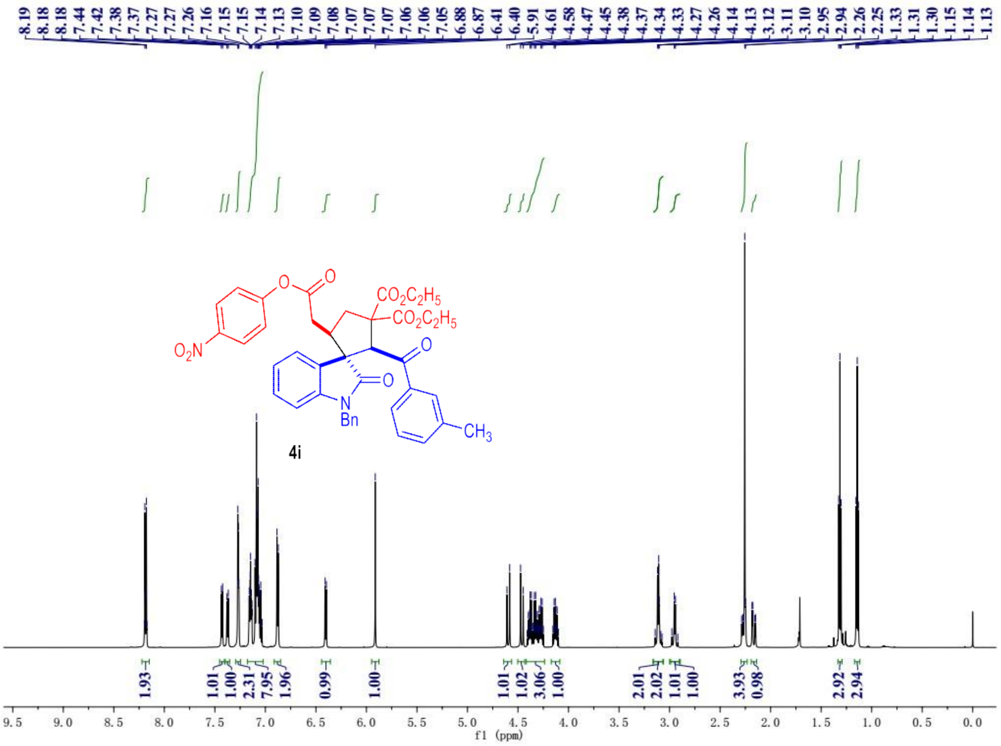

Supplementary Figure S50 ^{1}H NMR spectrum of **4i** (600 MHz, $CDCl_3$)

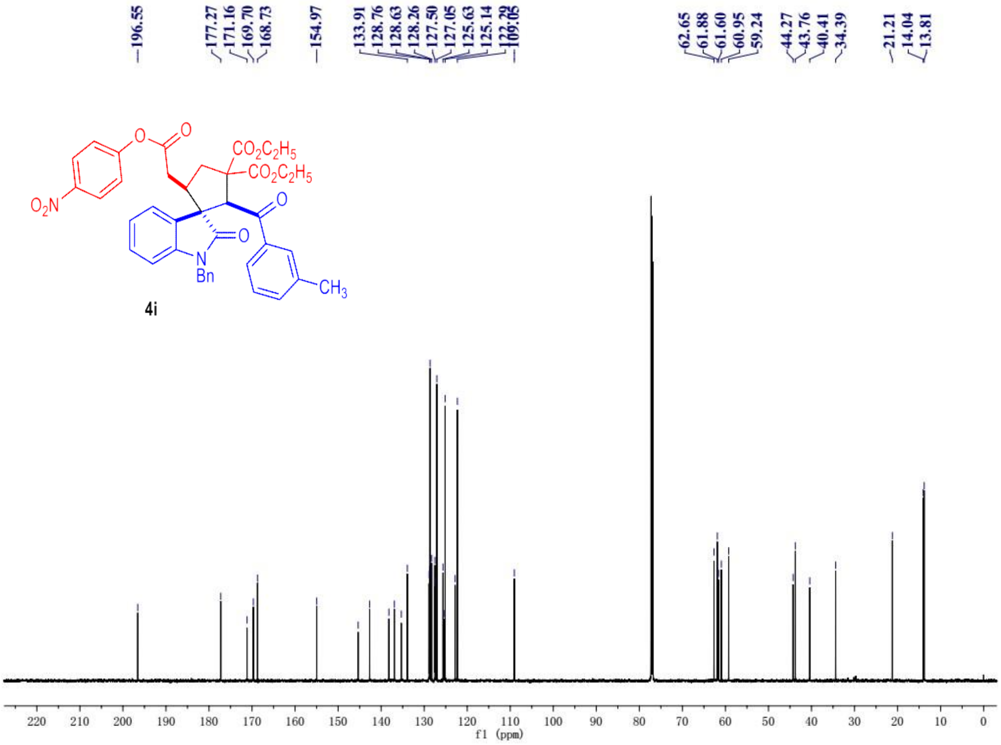

Supplementary Figure S51 ^{13}C NMR spectrum of **4i** (151 MHz, $CDCl_3$)

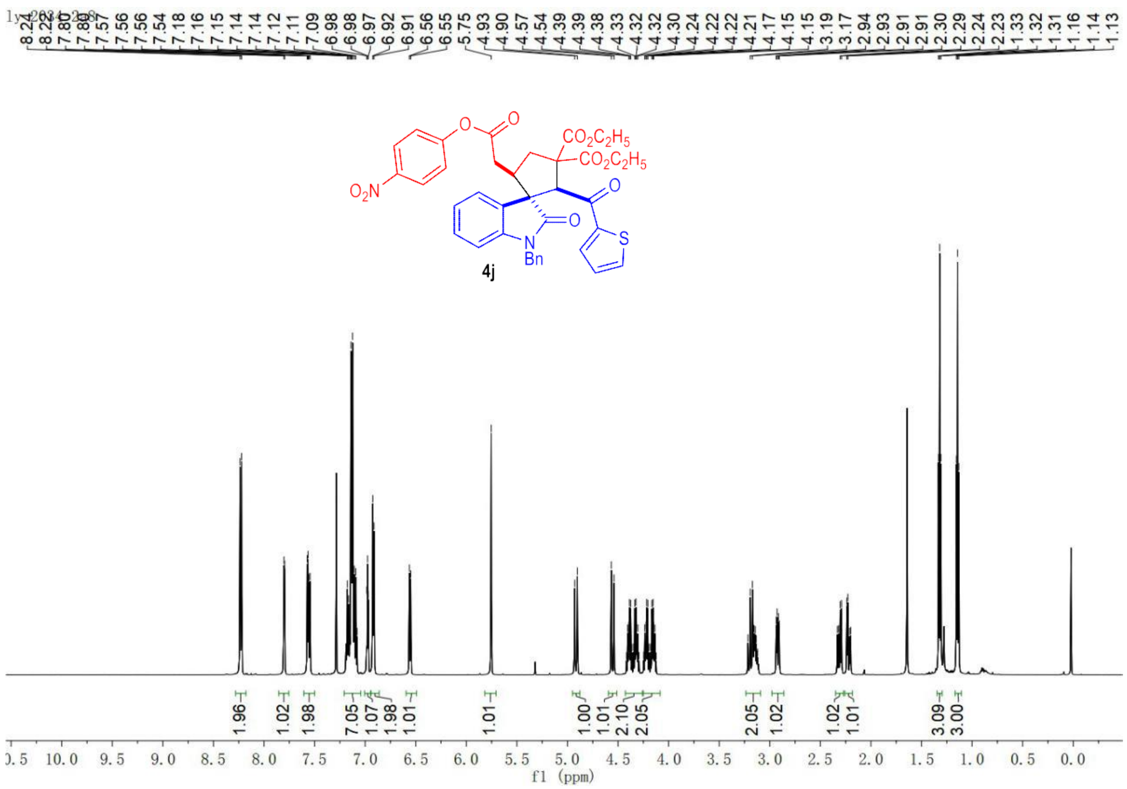

Supplementary Figure S52 ^{1}H NMR spectrum of **4j** (600 MHz, $CDCl_3$)

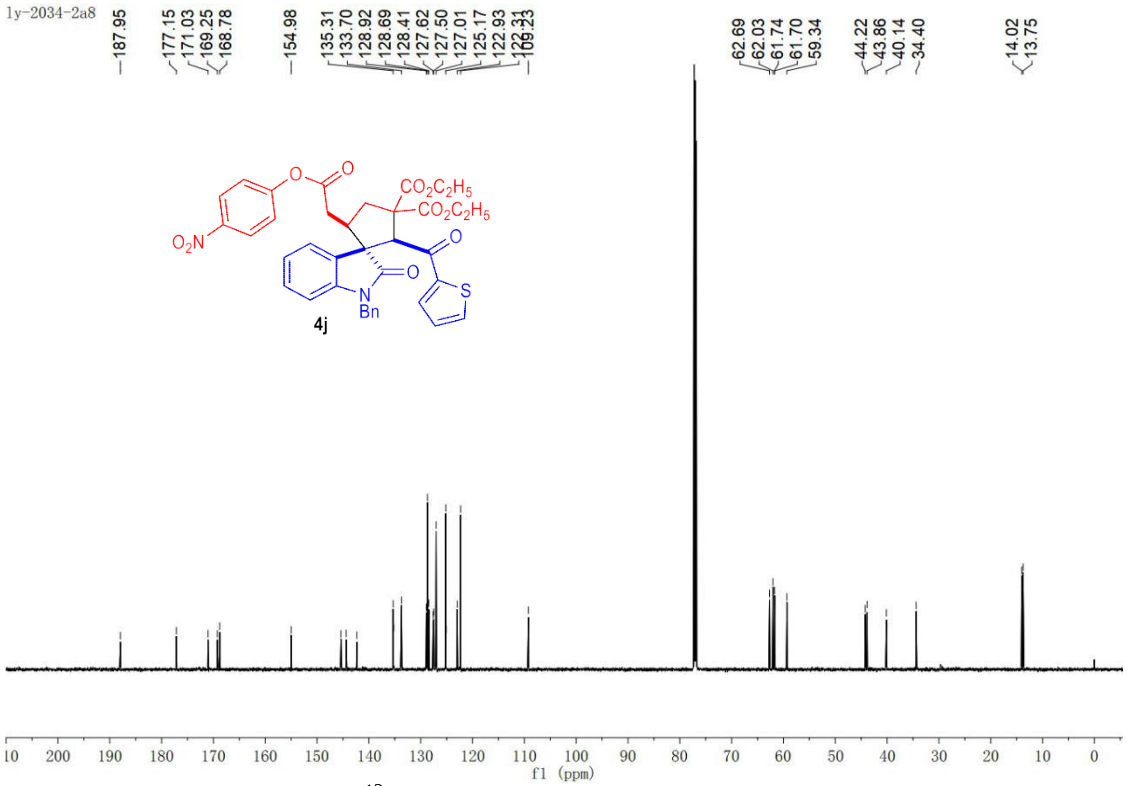

Supplementary Figure S53 ^{13}C NMR spectrum of **4j** (151 MHz, $CDCl_3$)

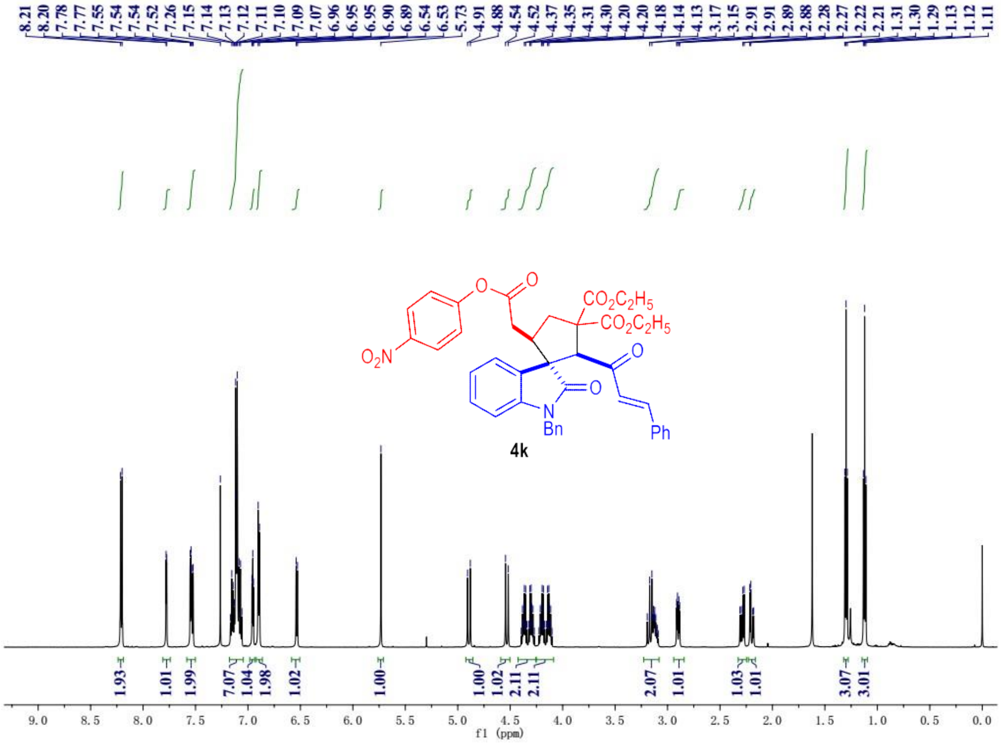

Supplementary Figure S54 ^{1}H NMR spectrum of **4k** (600 MHz, $CDCl_3$)

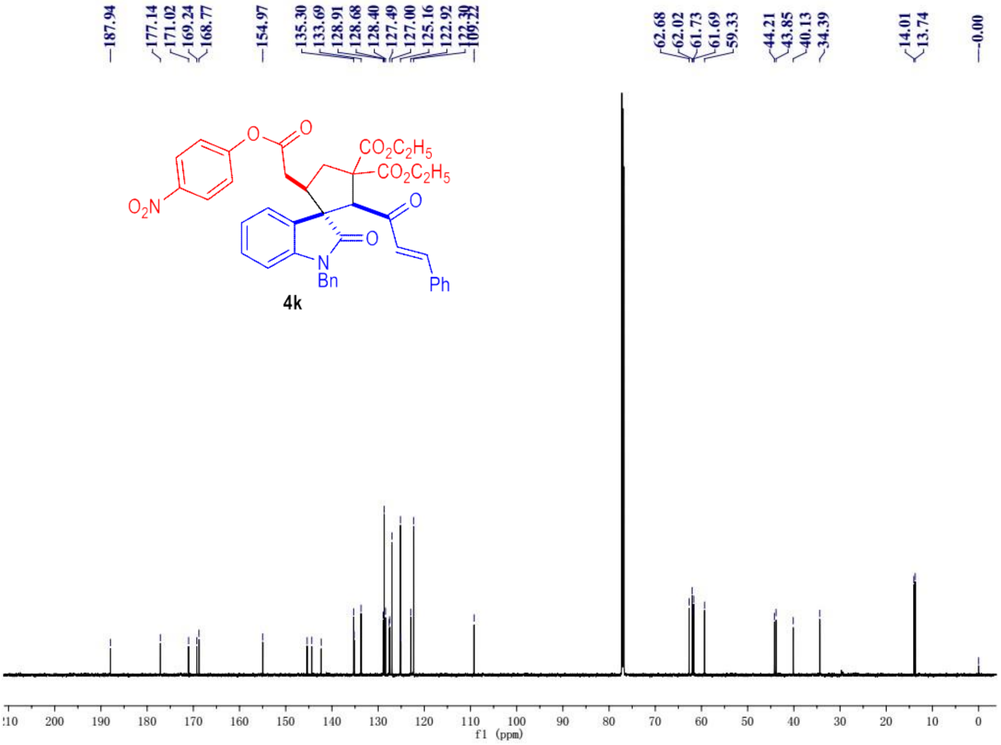

Supplementary Figure S55 ^{13}C NMR spectrum of **4k** (151 MHz, $CDCl_3$)

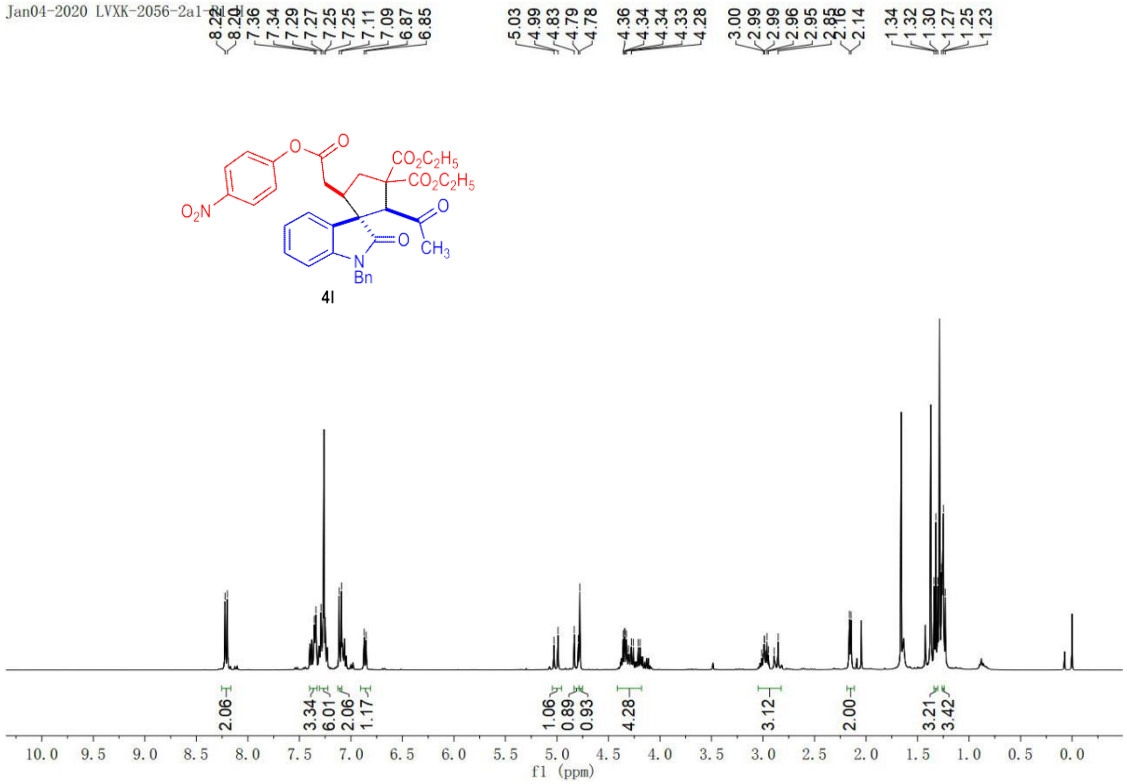

Supplementary Figure S56 ^{1}H NMR spectrum of **4l** (600 MHz, $CDCl_3$)

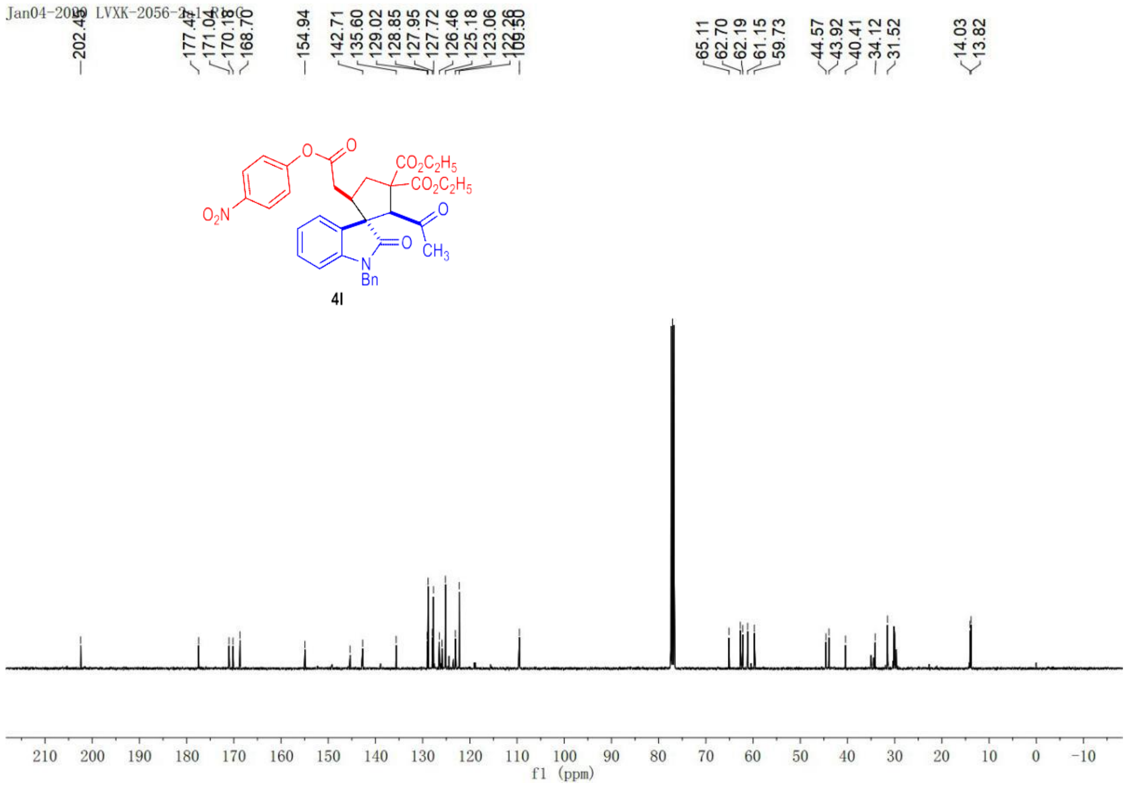

Supplementary Figure S57 ^{13}C NMR spectrum of **4l** (151 MHz, $CDCl_3$)

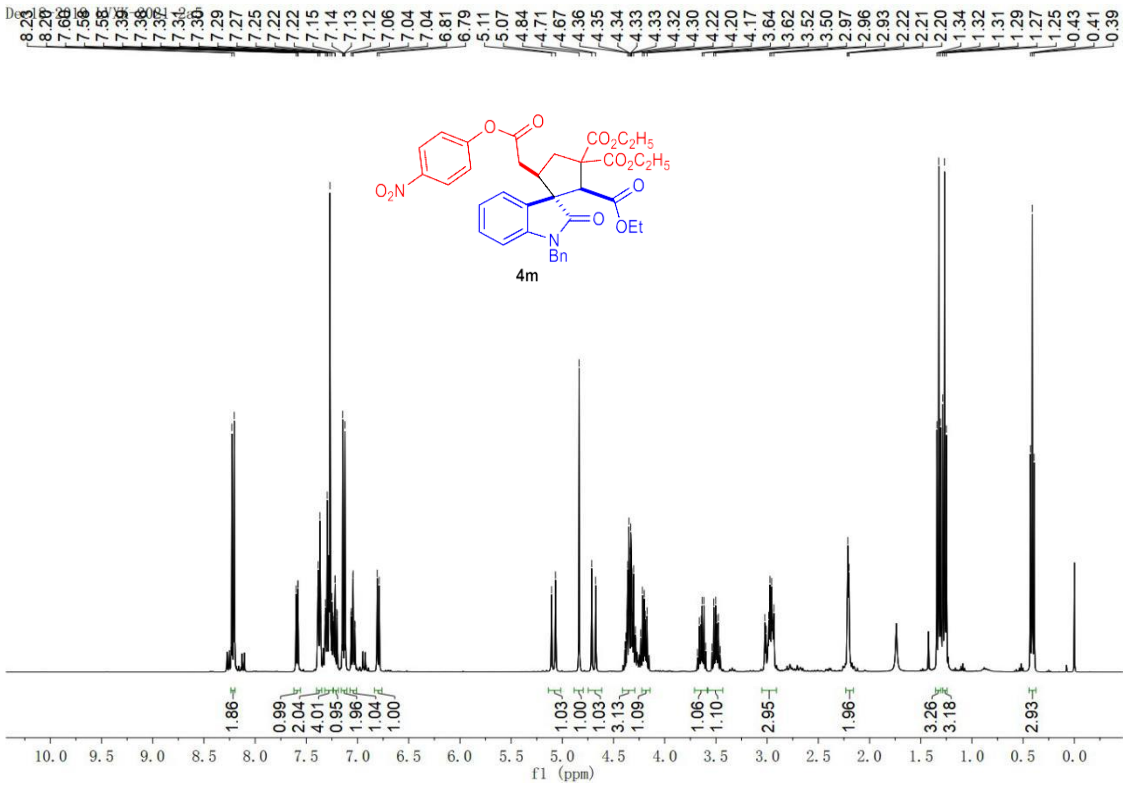

Supplementary Figure S58 ^{1}H NMR spectrum of **4m** (600 MHz, $CDCl_3$)

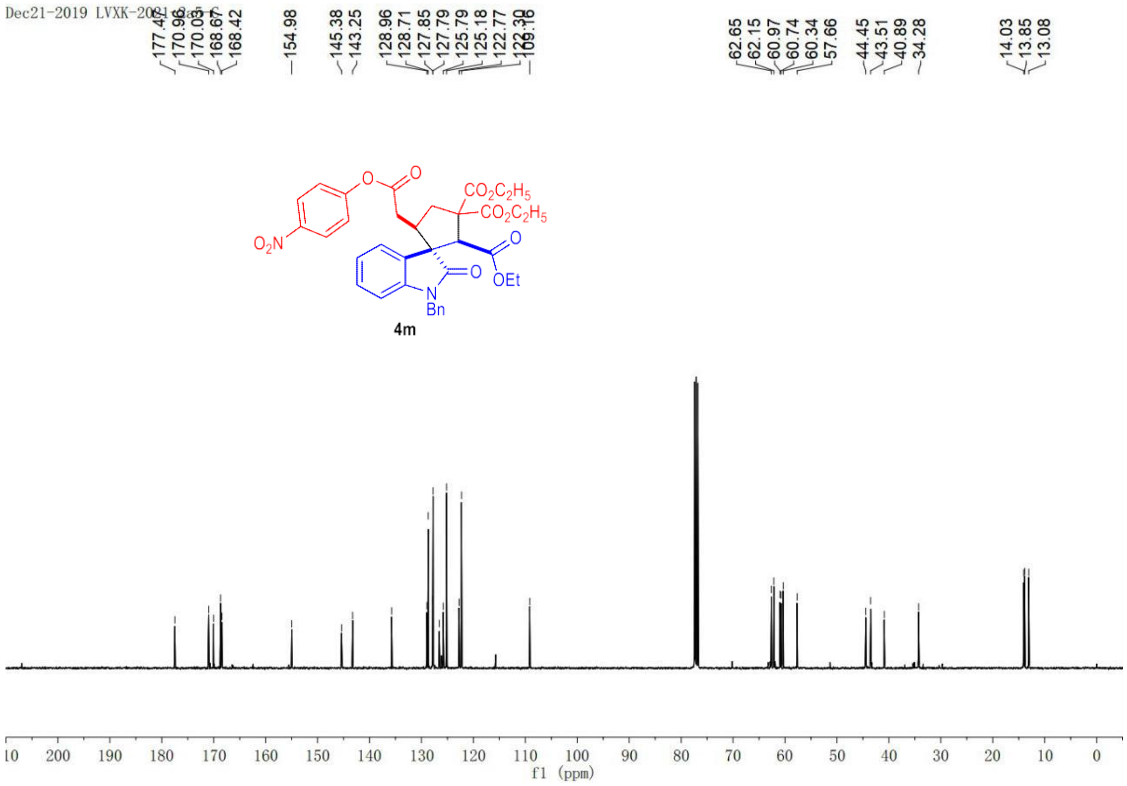

Supplementary Figure S59 ^{13}C NMR spectrum of **4m** (151 MHz, $CDCl_3$)

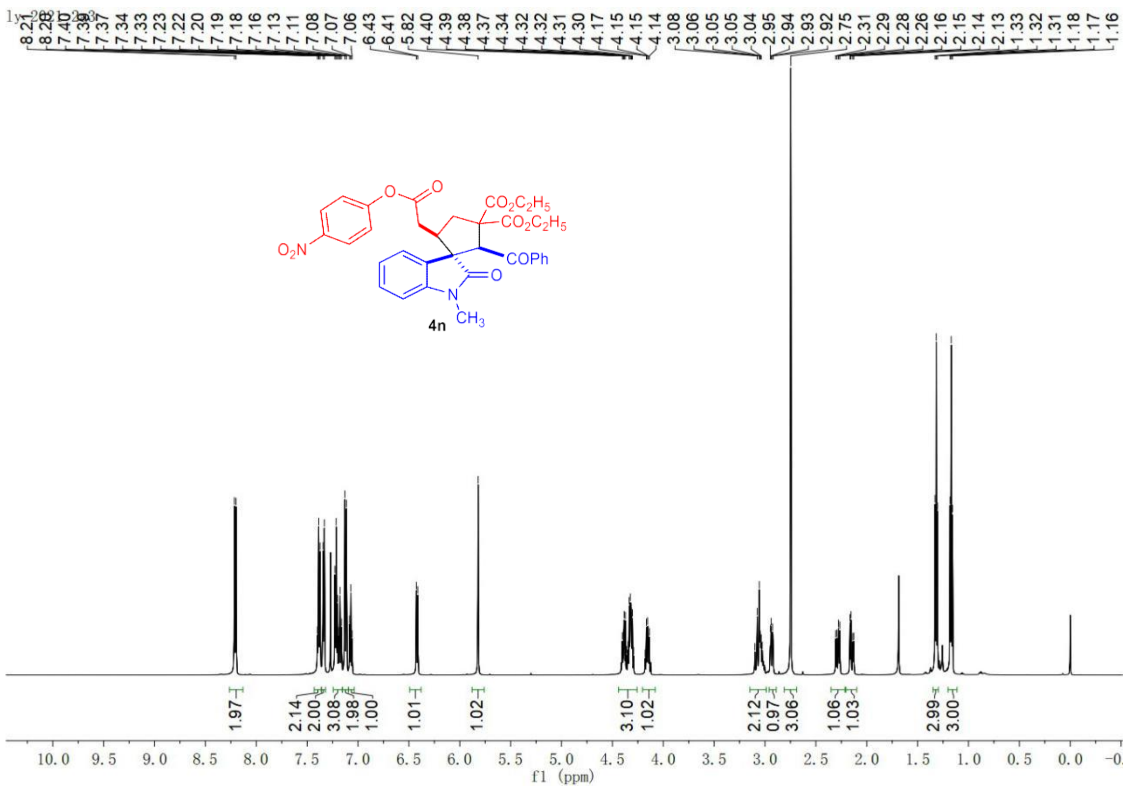

Supplementary Figure S60 ^{1}H NMR spectrum of **4n** (600 MHz, $CDCl_3$)

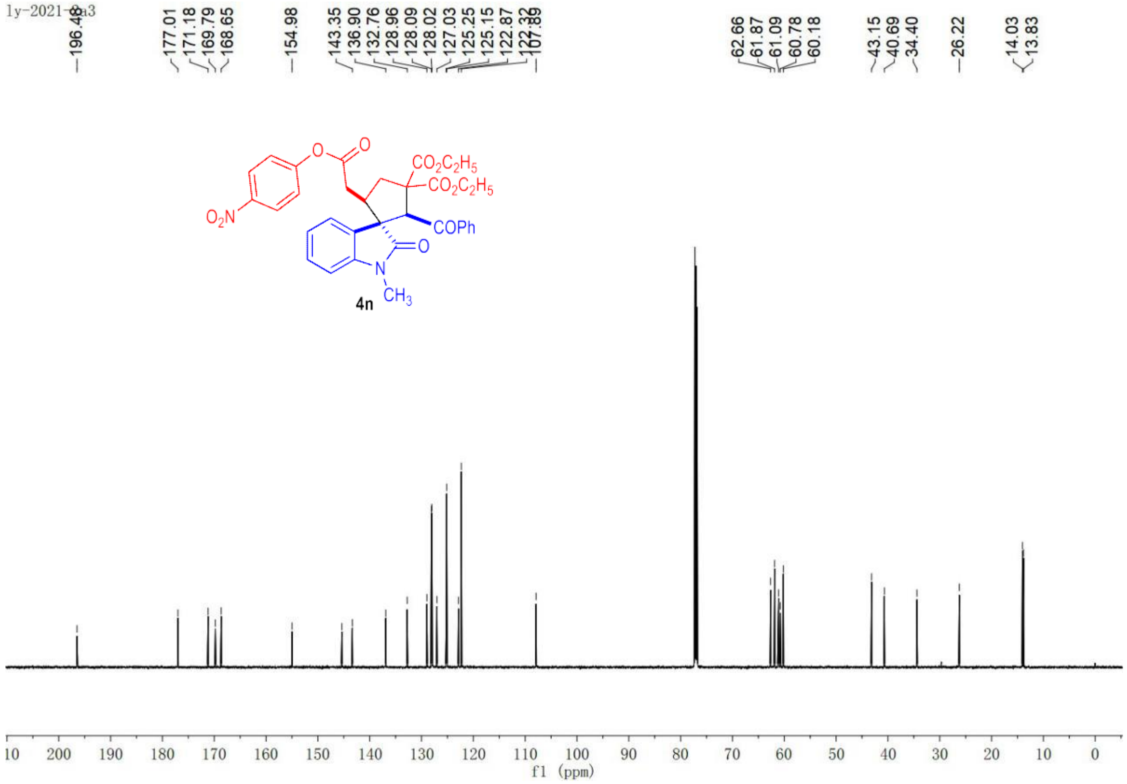

Supplementary Figure S61 ^{13}C NMR spectrum of **4n** (151 MHz, $CDCl_3$)

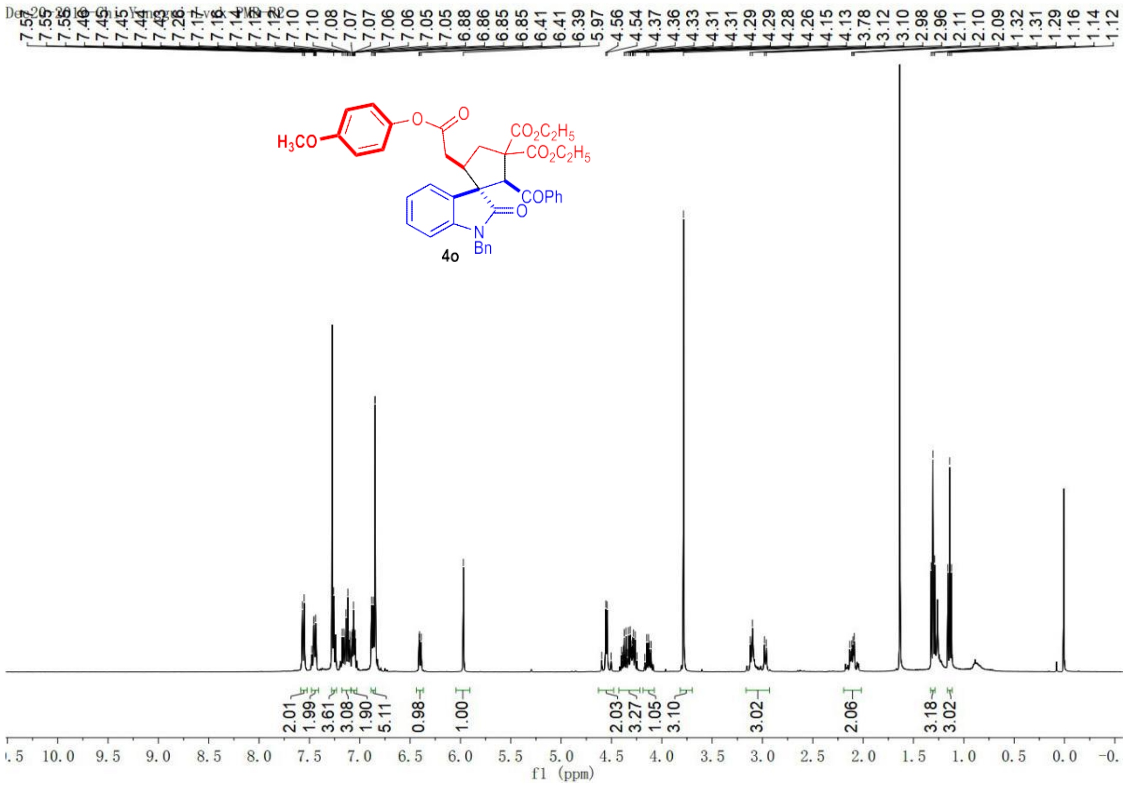

Supplementary Figure S62 ^{1}H NMR spectrum of **4o** (600MHz, $CDCl_3$)

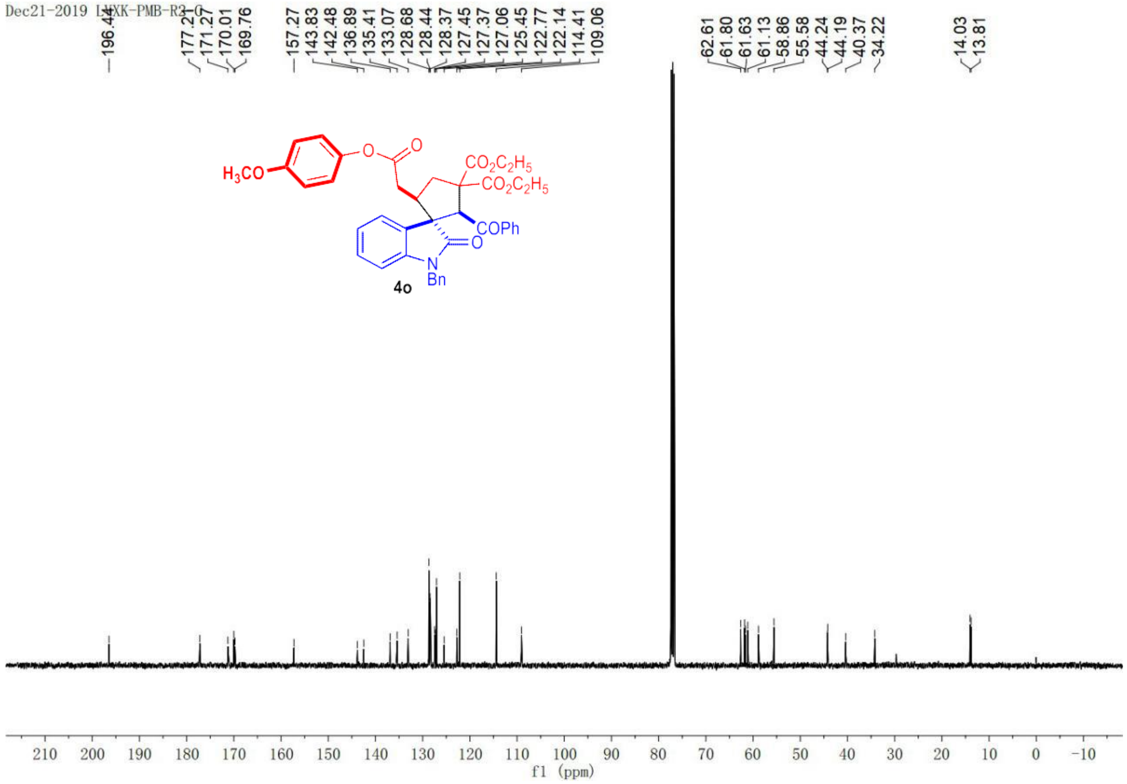

Supplementary Figure S63 ^{13}C NMR spectrum of **4o** (151 MHz, $CDCl_3$)

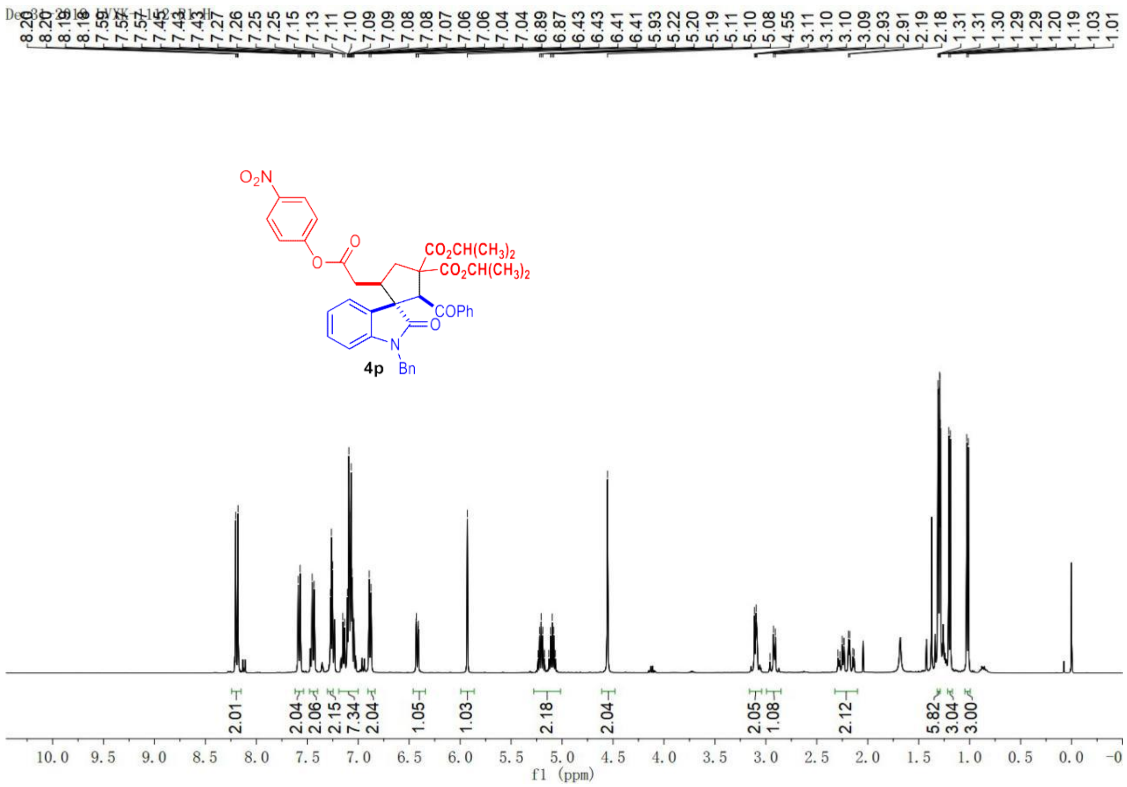

Supplementary Figure S64 ^{1}H NMR spectrum of **4p** (600 MHz, $CDCl_3$)

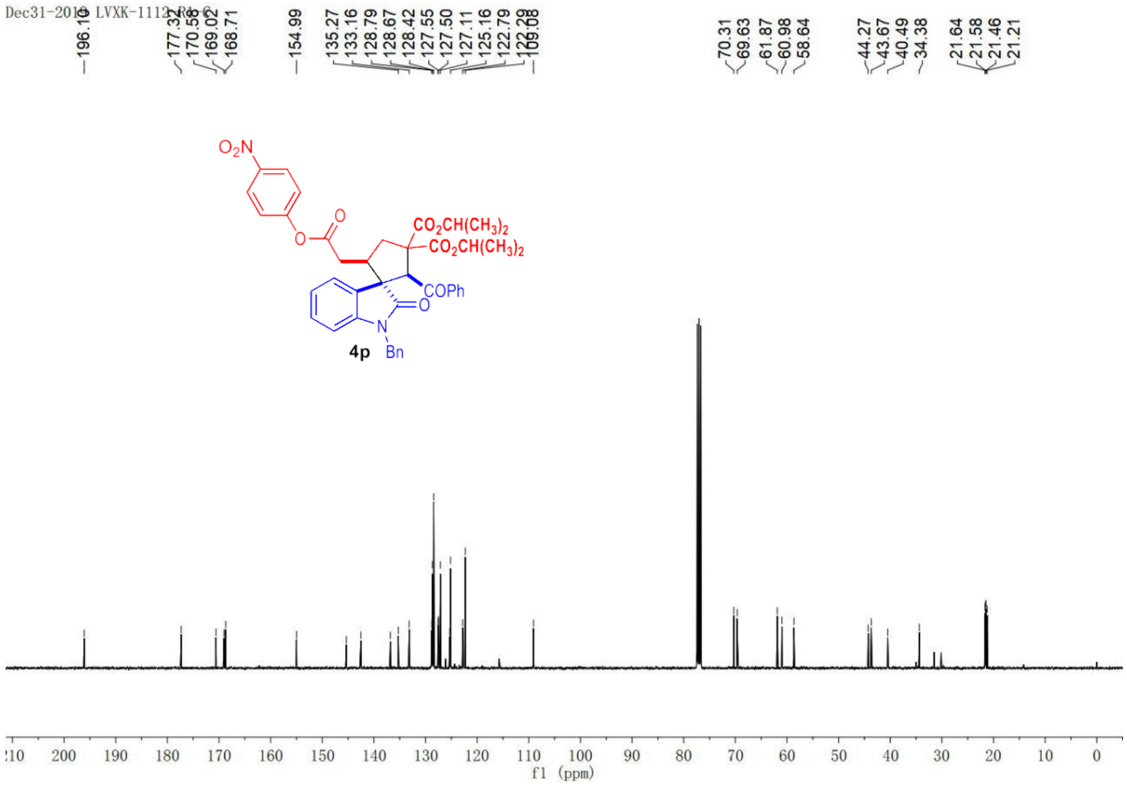

Supplementary Figure S65 ^{13}C NMR spectrum of **4p** (151 MHz, $CDCl_3$)

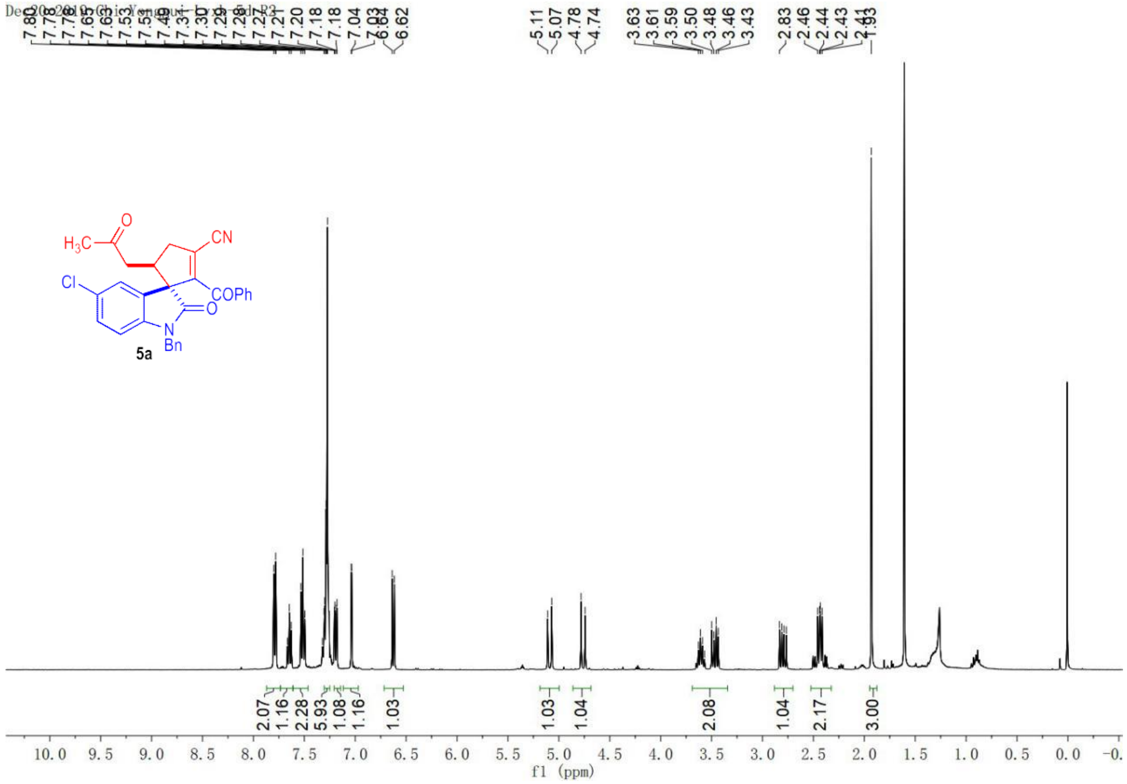

Supplementary Figure S66 ^{1}H NMR spectrum of **5a** (600 MHz, $CDCl_3$)

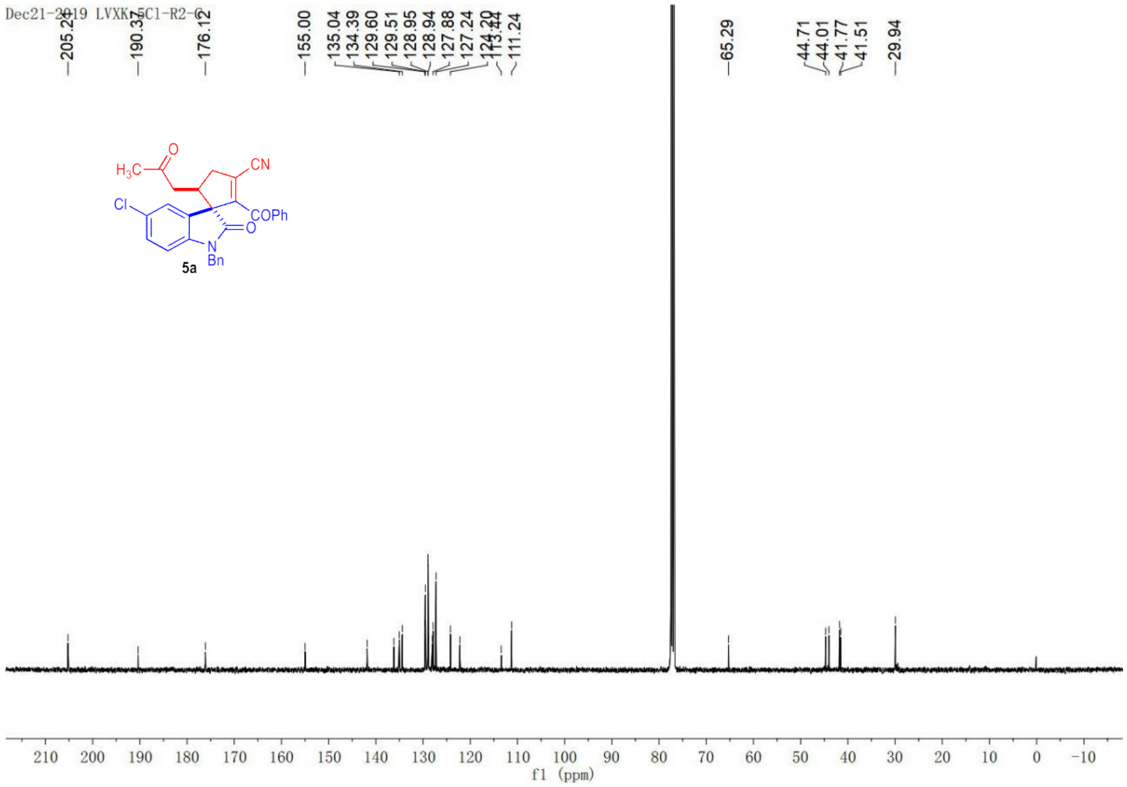

Supplementary Figure S67 ^{13}C NMR spectrum of **5a** (151 MHz, $CDCl_3$)

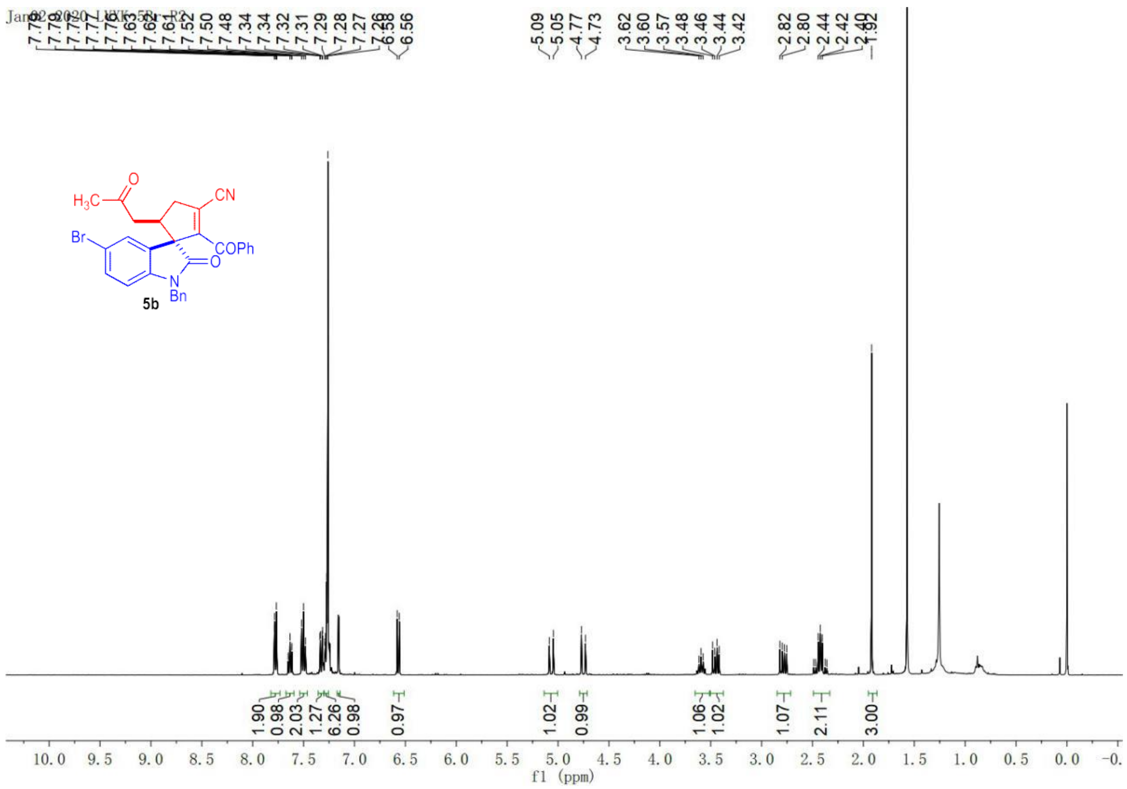

Supplementary Figure S68 ^{1}H NMR spectrum of **5b** (600MHz, $CDCl_3$)

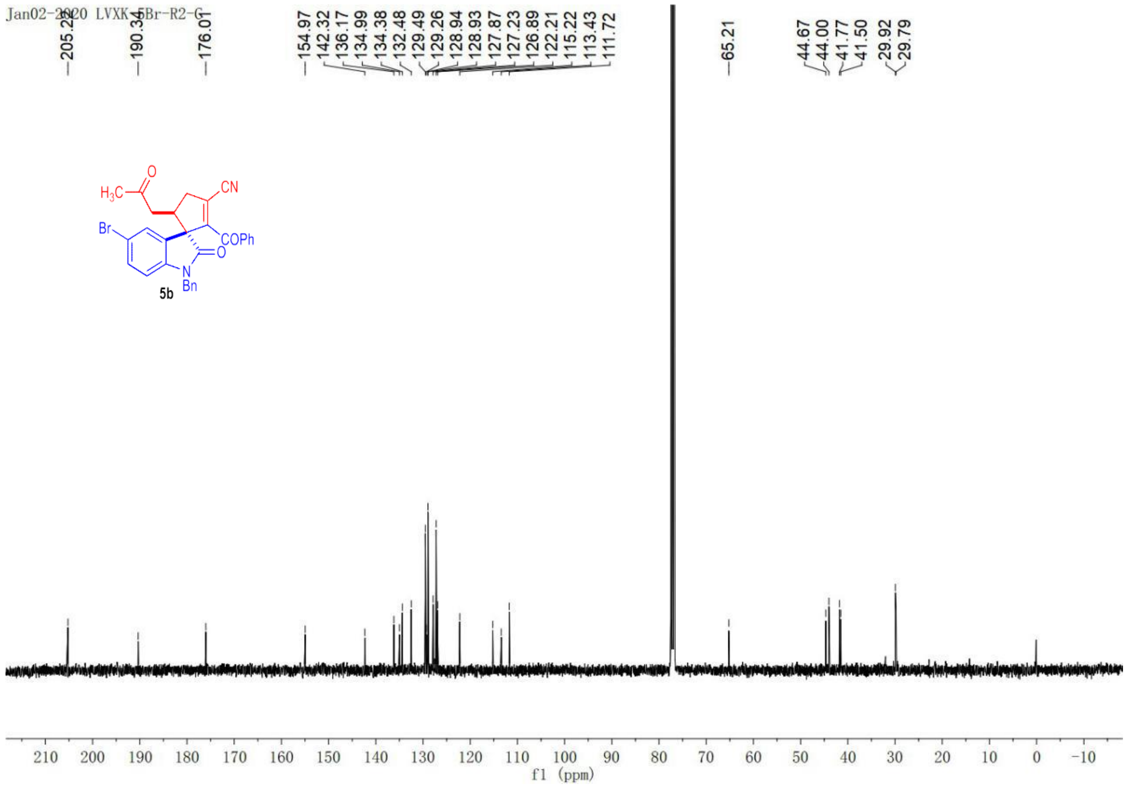

Supplementary Figure S69 ^{13}C NMR spectrum of **5b** (151 MHz, $CDCl_3$

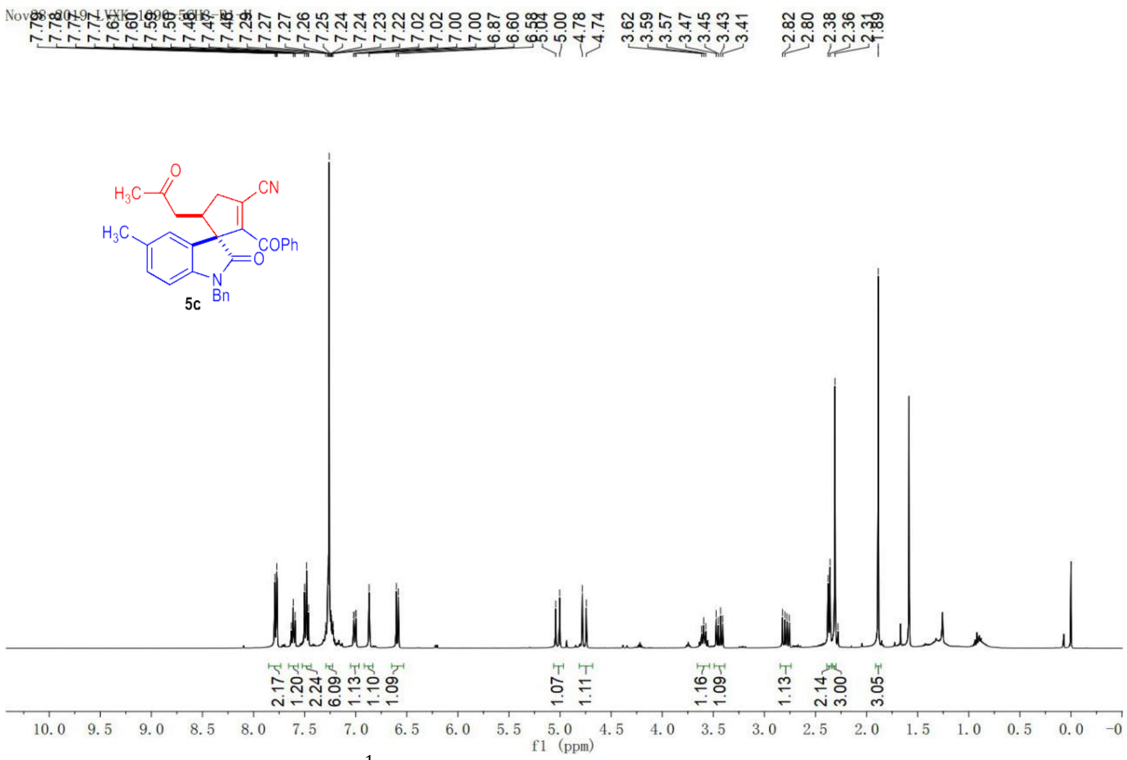

Supplementary Figure S70 ^{1}H NMR spectrum of **5c** (600 MHz, $CDCl_3$)

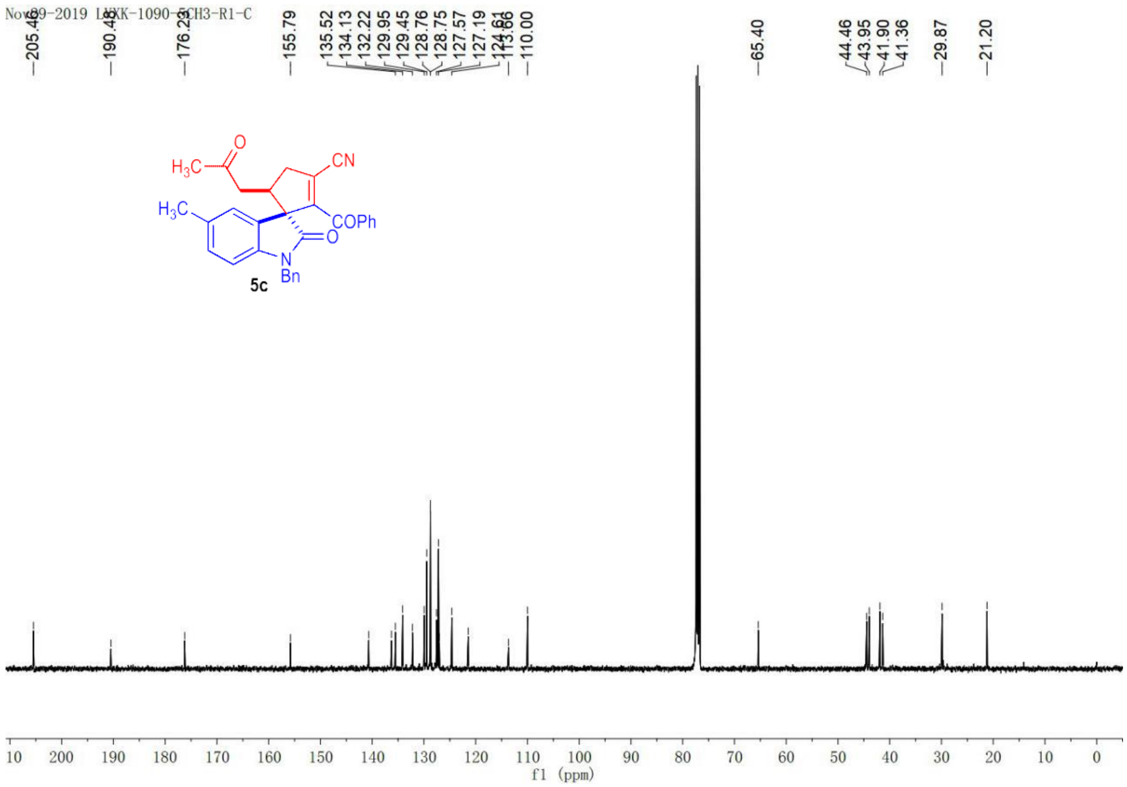

Supplementary Figure S71 ^{13}C NMR spectrum of **5c** (151 MHz, $CDCl_3$)

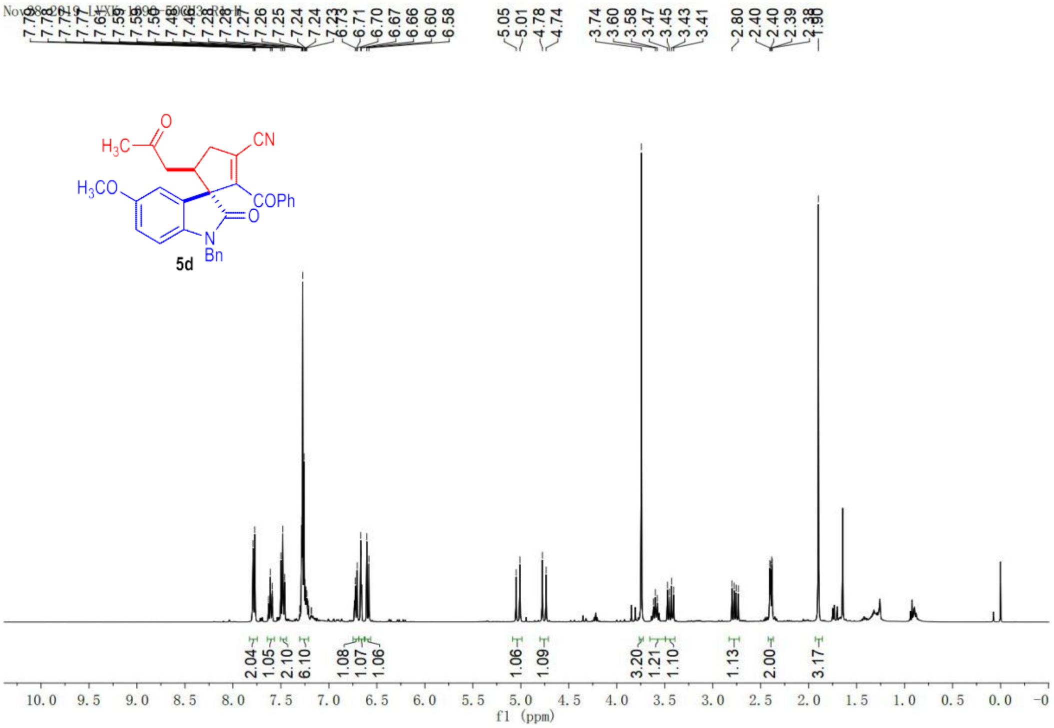

Supplementary Figure S72 ^{1}H NMR spectrum of **5d** (600 MHz, $CDCl_3$)

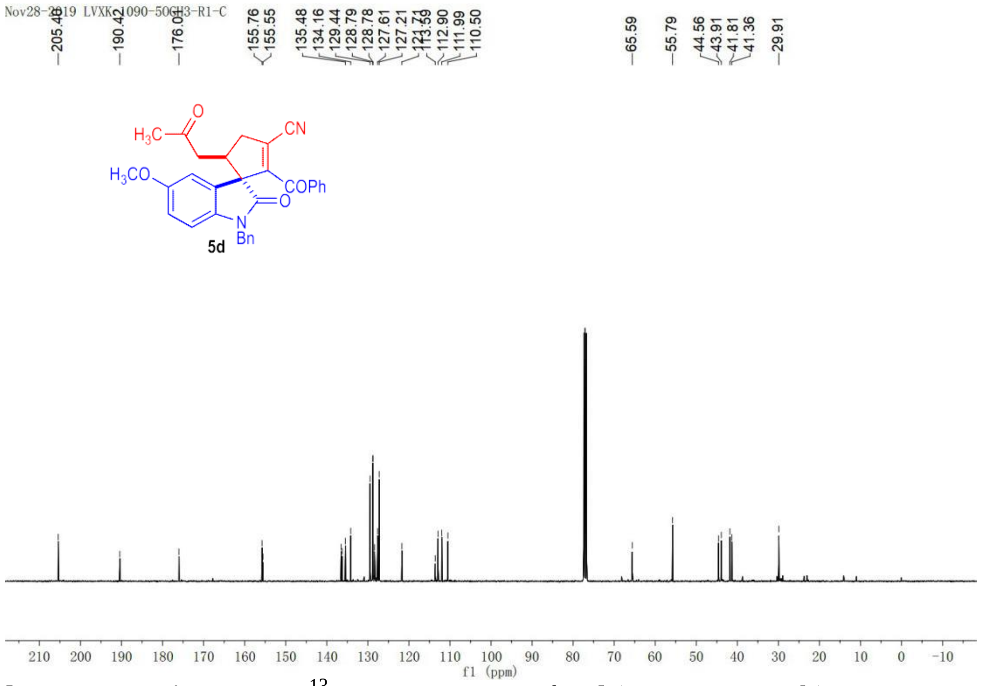

Supplementary Figure S73 ^{13}C NMR spectrum of **5d** (151 MHz, $CDCl_3$)

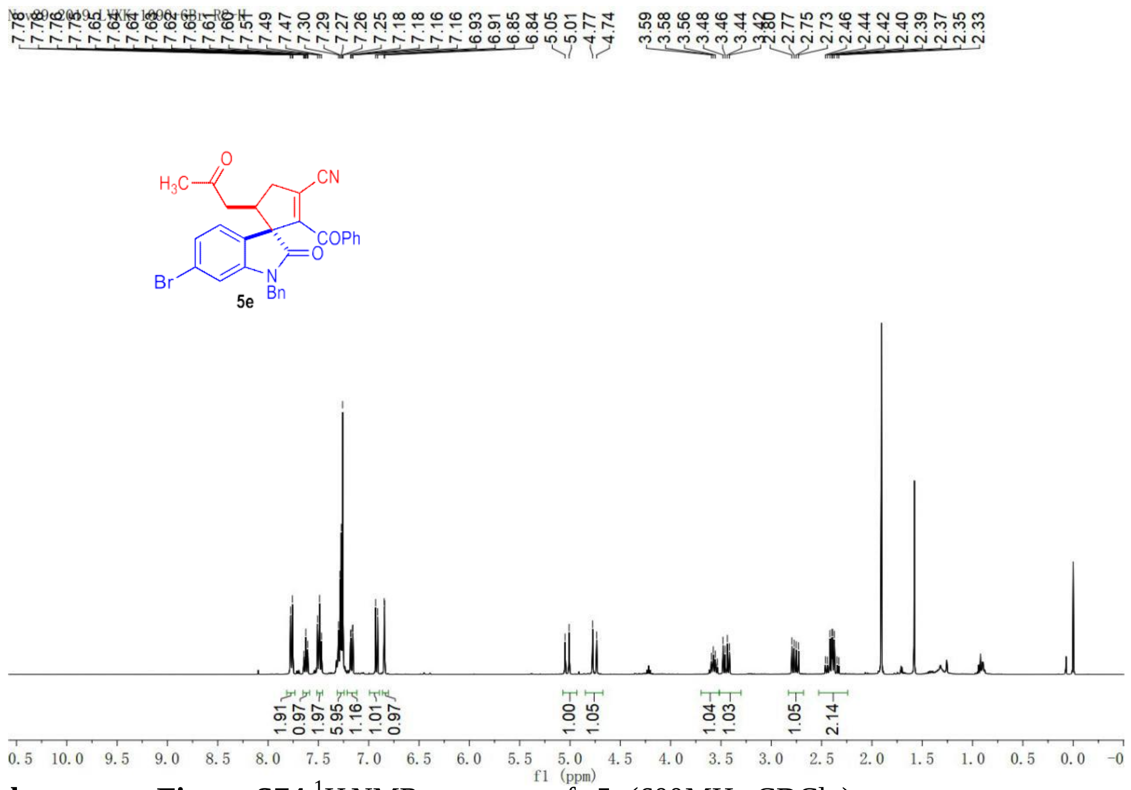

Supplementary Figure S74 ^{1}H NMR spectrum of **5e** (600 MHz, $CDCl_3$)

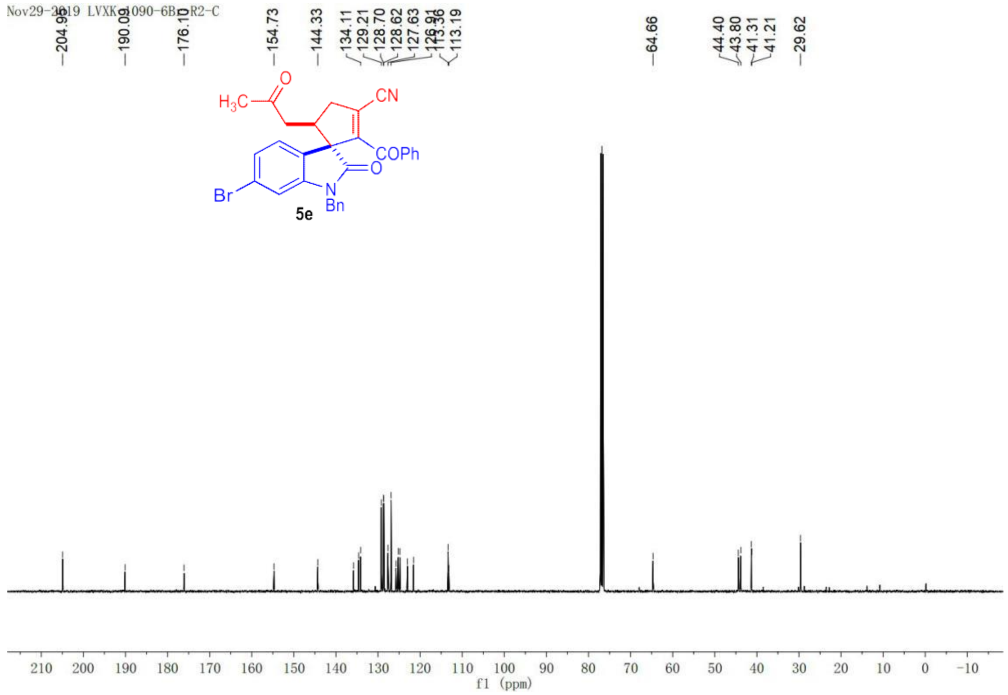

Supplementary Figure S75 ^{13}C NMR spectrum of **5e** (151 MHz, $CDCl_3$)

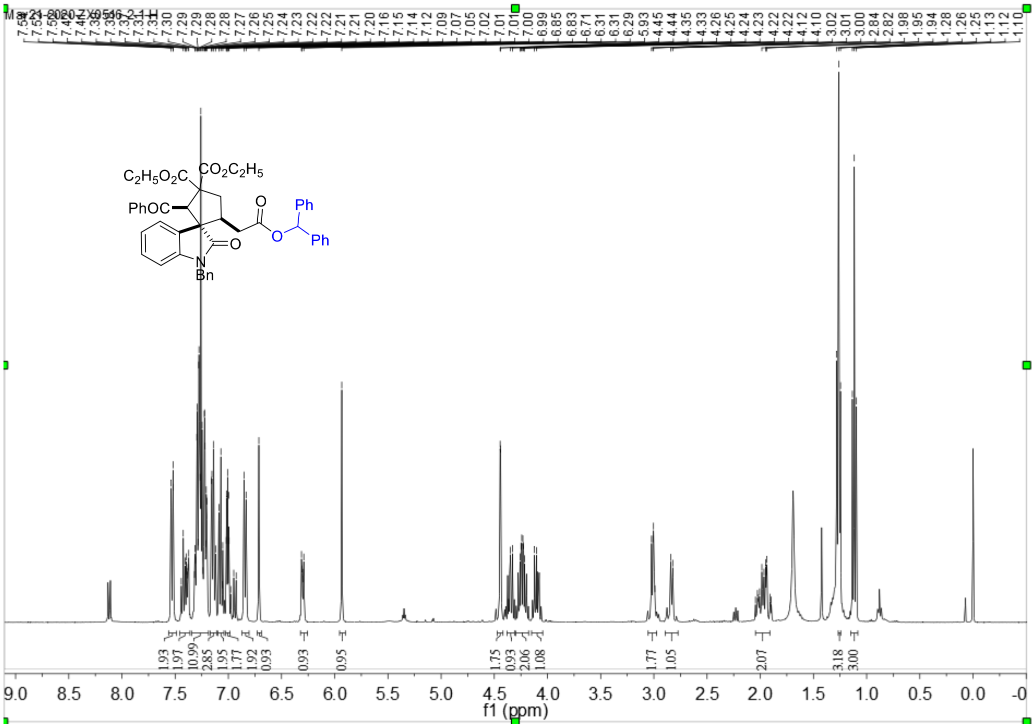

Supplementary Figure S76 ^{1}H NMR spectrum of 7 (400 MHz, CDCl3)

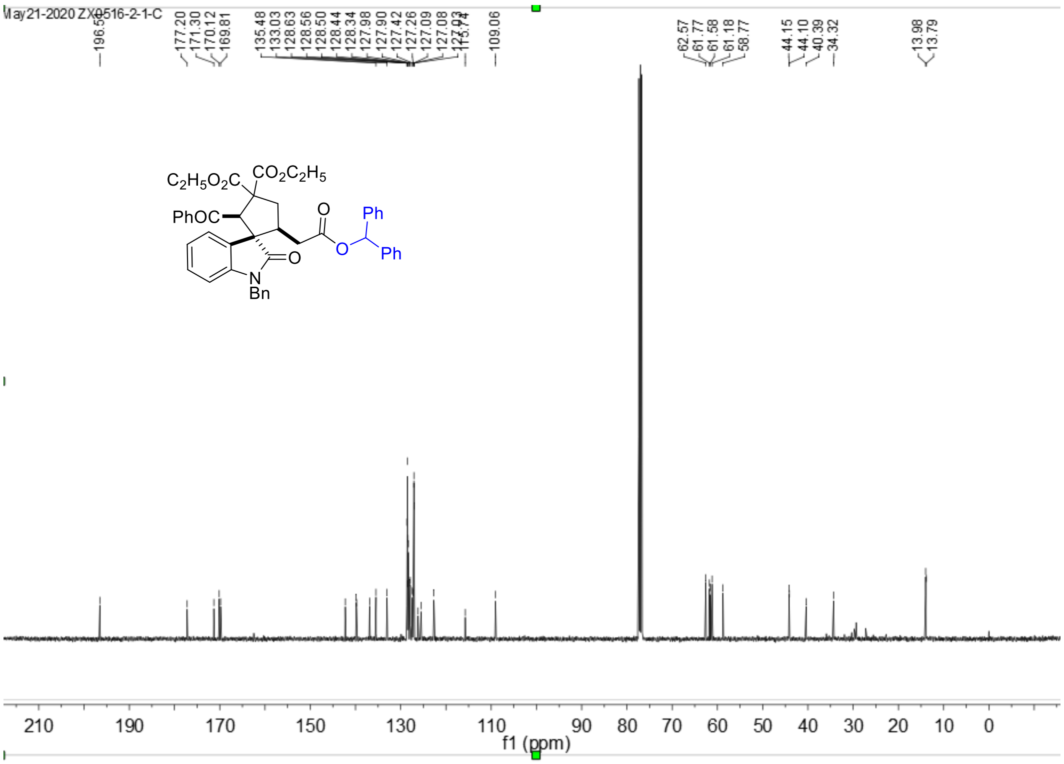

Supplementary Figure S77 ^{13}C NMR spectrum of 7 (101 MHz, CDCl3)
